# Food consumption and the actual statistics of cardiovascular diseases: an epidemiological comparison of 42 European countries

**DOI:** 10.3402/fnr.v60.31694

**Published:** 2016-09-27

**Authors:** Pavel Grasgruber, Martin Sebera, Eduard Hrazdira, Sylva Hrebickova, Jan Cacek

**Affiliations:** Faculty of Sports Studies, Masaryk University, Brno, Czech Republic

**Keywords:** prevention, BMI, smoking, food consumption

## Abstract

**Background:**

The aim of this ecological study was to identify the main nutritional factors related to the prevalence of cardiovascular diseases (CVDs) in Europe, based on a comparison of international statistics.

**Design:**

The mean consumption of 62 food items from the FAOSTAT database (1993–2008) was compared with the actual statistics of five CVD indicators in 42 European countries. Several other exogenous factors (health expenditure, smoking, body mass index) and the historical stability of results were also examined.

**Results:**

We found exceptionally strong relationships between some of the examined factors, the highest being a correlation between raised cholesterol in men and the combined consumption of animal fat and animal protein (*r*=0.92, *p*<0.001). The most significant dietary correlate of low CVD risk was high total fat and animal protein consumption. Additional statistical analyses further highlighted citrus fruits, high-fat dairy (cheese) and tree nuts. Among other non-dietary factors, health expenditure showed by far the highest correlation coefficients. The major correlate of high CVD risk was the proportion of energy from carbohydrates and alcohol, or from potato and cereal carbohydrates. Similar patterns were observed between food consumption and CVD statistics from the period 1980–2000, which shows that these relationships are stable over time. However, we found striking discrepancies in men's CVD statistics from 1980 and 1990, which can probably explain the origin of the ‘saturated fat hypothesis’ that influenced public health policies in the following decades.

**Conclusion:**

Our results do not support the association between CVDs and saturated fat, which is still contained in official dietary guidelines. Instead, they agree with data accumulated from recent studies that link CVD risk with the high glycaemic index/load of carbohydrate-based diets. In the absence of any scientific evidence connecting saturated fat with CVDs, these findings show that current dietary recommendations regarding CVDs should be seriously reconsidered.

The relationship between nutrition and the prevalence of diseases is a very controversial and hotly debated topic, and the research conducted during the last decades has often produced conflicting results. For example, recent meta-analyses seriously challenge the role of saturated fat as the fundamental trigger of cardiovascular diseases (CVDs) (1–6), which was a prevailing hypothesis for several decades. The most complex analysis of Mente et al. (1) identified ‘Mediterranean’ and ‘high-quality’ dietary patterns, vegetables, nuts, ‘prudent diets’ (including a lot of vegetables, fruit, legumes, whole grains and fish), and monounsaturated fatty acids (MUFAs) as the only dietary components, which are strongly and consistently related to low risk of coronary heart disease (CHD) in observational or clinical studies. Trans-fatty acids, high glycaemic index/load, and the ‘Western’ diet (including processed and red meat, butter, high-fat dairy products, eggs, and refined cereals) were strongly and consistently related to high CHD risk. However, there was only weak evidence for any connection between CHD and saturated fat, individual animal products (meat, eggs, milk), and fat as a whole.

The heterogeneity of results is not very surprising because long-term observational surveys routinely rely on self-reported consumption rates of selected food items and may be distorted by the existence of many hidden confounding factors. For change, controlled clinical trials are usually too short. Therefore, the fundamental weak point of all research related to health and nutrition is the lack of precise, long-term data on food intake.

One possible way to overcome this problem is an ecological approach based on the use of official national statistics of food intake, combined with the statistics of actual disease prevalence. We successfully used this methodology in our previous work dealing with the determinants of physical growth, using nutritional statistics from the FAOSTAT database (7). The results that we obtained were often impressive and remarkably agreed with the biological quality of consumed proteins. This positive experience shows that the data from the FAOSTAT database are reasonably reliable and could be used in ecological analyses examining the relationship between nutrition and human health.

Understandably, similar to other study designs, the ecological approach has its weaknesses. The most frequently cited objection is that relationships that are valid at the country level may not be valid at the individual level. And above all, the results may be distorted by unknown confounders. However, confounders appear in virtually all study designs and our experience with ecological analyses shows that their power is deeply underestimated. This sceptical view probably stems from the fact that health ecological studies conducted in the past routinely used only a few variables tested on small, disparate samples of culturally unrelated countries, which may be ascribed to the limited number of available statistics. Such restricted data, taken out of context, can hardly produce useful results. Indeed, to our knowledge, ecological studies dealing with CVDs have been limited to few indicators or few countries, and an extensive comparison of the FAOSTAT statistics with the prevalence of CVDs has never been carried out.

In contrast, the use of a very large number of meaningful variables enables a very comprehensive approach to the problem, and in our own ecological analyses, their list is virtually exhaustive. At the same time, we routinely observe that the most persuasive results are almost always connected with food items with the highest consumption rates, or with basic nutrients such as protein or fat intake. This is logical because the health effect of food consumption is usually associated with food ingredients that make up the largest proportion in the diet. The smaller the consumption, the higher the likelihood of a spurious result, and here, the ecological approach reaches its limits. Therefore, attention should be focused to basic foodstuffs.

Understandably, even strong ecological trends in the incidence of diseases cannot be regarded as definitive proof of causal relationships at the individual level. However, the validity of such observations can be supported by studies using different methodologies. Furthermore, these findings could also significantly contribute to the identification of dietary components, whose true health effects can subsequently be examined in controlled clinical trials.

Having all possible advantages and limitations in mind, we decided to examine whether FAOSTAT statistics could produce some meaningful results in relation to the prevalence/incidence of the main indicators of cardiovascular health in Europe. The choice of examined countries was purposely limited to Europe because data from the developing world could be far less reliable. In accordance with Bradford Hill criteria (8), we decided to view the problem in the broadest possible context, examining even historical changes in the relationship between food consumption and CVD indicators.

## Methods

### Sources

The actual statistics of CVD indicators were drawn from the European Cardiovascular Disease Statistics 2012 by Nichols et al. (9) (see Supplementary Dataset, Sheet 1), which were compiled mainly using World Health Organization databases. The data on the prevalence of raised blood cholesterol (>5 mmol/L), raised fasting blood glucose (>7 mmol/L, or on medication), and raised blood pressure (systolic ≥140 or diastolic ≥90 mmHg, or blood pressure medication use) came from 2008. Mean body mass index (BMI) values (kg/m^2^) were computed from the period 1990–2008 (years 1990, 1995, 2000, 2005–2008). The mean prevalence of smoking was based on all available data from the period 1990–2009 (years 1990–1994, 1995–1999, and annual data from 2000–2009). In addition, for another support of the results, we also included the statistics of mean cholesterol levels, mean systolic blood pressure, and mean blood glucose levels from 2008, but they were used only for simple Pearson linear correlations because their data were largely duplicate. Understandably, the risk of CVDs also depends on other factors (genetics, stress, physical activity), but they were not targeted in the present study because their role is much more difficult to assess. For example, the data on physical activity listed by Nichols et al. (9) were self-reported and available from only 34 countries.

Age-standardised total CVD mortality rates and CHD mortality rates (for all ages, per 100,000 people) were obtained from Nichols et al. (10). The data came from the most recent available year in the period 2004–2011, but the overwhelming majority was from 2009–2011. These age-standardised statistics were further supplemented by mortality rates below 65 years.

The information on food consumption was obtained from the database of FAOSTAT [food balance > food supply (7)]. The FAOSTAT website states that ‘The food balance sheet shows for each food item i.e. each primary commodity availability for human consumption which corresponds to the sources of supply and its utilisation. The total quantity of foodstuffs produced in a country added to the total quantity imported and adjusted to any change in stocks that may have occurred since the beginning of the reference period gives the supply available during that period’.

Using this database, we collected daily consumption rates (grams per capita) of 53 foodstuffs. We included all main food items and even some subcategories, whose annual per capita consumption was higher than 5 kg (13.7 g/day), concentrated sources of fat (olive oil, soybean oil, sunflower oil), and the general indicators of fat, protein, and energy intake. In addition to that, we computed nine additional food items (the energy from carbohydrates and alcohol, potato and cereal carbohydrates, and plant food; animal fat and animal protein; total fat and animal protein; total fat and total protein; pork and beef fat) in order to reveal more subtle relationships. The data of daily energy intake from potato and cereal carbohydrates (PC CARB energy) and carbohydrates and alcohol (CA energy) were derived from daily protein and fat intake, assuming 4.1 kcal per gram of protein and 9 kcal per gram of fat. The proportion of energy from plant food was computed from the total energy intake from plant products, minus alcoholic beverages. Food items showing no correlation with CVD indicators (tea, spices) were excluded. Because the negative influence of food on human health is a long-term process, an average for the period 1993–2008 was calculated for each item (see Supplementary Dataset, Sheets 2,3). Altogether, 62 food items were used for the subsequent analysis.

The total sample used in our study consisted of 42 European countries, including Armenia, Azerbaijan, and Georgia, but excluding Turkey and Montenegro (for which some statistics were lacking). As a possible confounding variable, we also included health expenditure (for 2008 and 1995–2008) (according to the World Bank (11)). Life expectancy at birth (for 2008, according to the World Bank (12)) was included as a supplementary variable as well because it is very closely associated with cardiovascular health.

### Statistical analysis

Using the software SPSS Statistics 24.0, the relationships between CVD indicators and various independent variables (food consumption, health expenditure, smoking, BMI, and raised cholesterol) were first investigated using simple Pearson linear correlations.

Furthermore, we conducted a factor analysis of all the examined variables. The factor analysis groups variables with similar characteristics and because it combines multiple factors that influence the grouping of variables, it already solves a lot of problems associated with multicollinearity, which was the key statistical problem in the present study. Indeed, it proved to be a very useful tool because it graphically separated specific food groups according to the geographical pattern of their consumption.

Other sophisticated procedures used to analyse a large number of independent variables are the ridge regression, LASSO regression, and elastic net regression. In our present study, we used these regression analyses in the case of raised blood pressure and total CVD mortality (in both sexes). All these three methods are based on the penalisation (artificial lowering) of *beta* regression coefficients, which also helps to solve the problem of multicollinearity among the examined variables. The changing size of the penalisation creates different models with different prediction errors, and a model with the lowest prediction error (ideally using low penalisation) is selected as optimal. The ridge regression works with all variables, while the LASSO regression is more selective and with the increasing penalisation, it shrinks the majority of *beta* coefficients to zero. The elastic net regression is basically a combination of these two methods and overcomes their shortcomings (13). To improve the quality of the regression models, we used the bootstrapping method, which works with random combinations of a different number of independent variables for each penalisation level, creates many additional models, and then calculates their mean result. Thus, this method takes into account the variability of results and reduces the impact of various anomalies. Because the general statistical goal is to achieve a good model with the lowest possible number of variables, we did not use the ‘optimal models’ with the lowest prediction errors, but more selective parsimonious (‘economical’) models that achieve the best ratio between the number of variables and the prediction error.

Subsequently, we used an analogy of the fixed effect models and studied temporal changes of correlations between CVD indicators and food consumption in single 16 years during the period 1993–2008. This procedure helped to identify long-term collinearity among the key food items, via the comparison of their trend lines. A standard statistical procedure used for the comparison of two trend lines is the regression slope test (14). A weakness of this method lies in its inability to objectively compare two trend lines that are markedly non-linear, which was often the case in this temporal comparison of *r* values. To overcome this limitation, we also used the dependent *t*-test and calculated a mean difference between 16 pairs of *r* values from the same year. A low standard deviation of such a mean difference indicates that the distance between *r* values of two trend lines remained basically constant in time. In other words, the *r* values of these two trend lines changed parallel to each other.

Finally, we performed a similar comparison of food consumption and the available CVD statistics from 1980, 1990, and 2000, again using the data of Nichols et al. (9).

## Results

### Pearson linear correlations

**Fig. 1:**
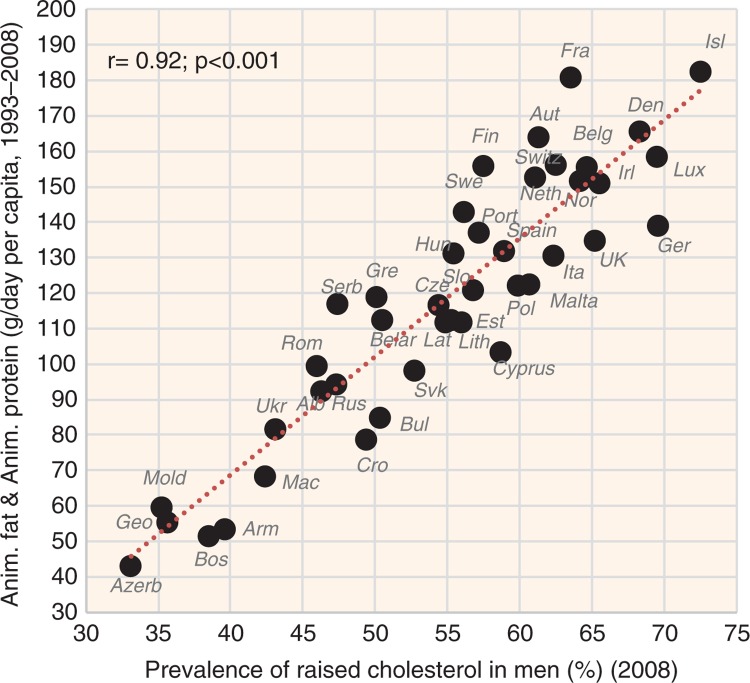
Correlation between the mean daily consumption of animal fat and animal protein and the prevalence of raised cholesterol levels in men (*r*=0.92; *p*<0.001).

Detailed results of the Pearson linear correlations are listed in the Supplementary Dataset, Sheet 5. A simplified version of these results is presented in Table 1. Our comparison shows that the strength of linear correlations in men's raised blood pressure, men's raised blood glucose, and CHD mortality in both sexes is moderate at best, but in other cases, it is exceptionally high. A particularly impressive finding is the relationship between raised cholesterol and animal fat (*r*=0.89 in men, *r*=0.87 in women; *p*<0.001). Interestingly, the *r* values further slightly increase, when animal fat is combined with animal protein: 0.92 in men (Fig. 1) and 0.88 in women. Evidently, a food most strongly contributing to these results is meat (*r*=0.83 in men, *r*=0.79 in women; *p*<0.001).

**Fig. 2:**
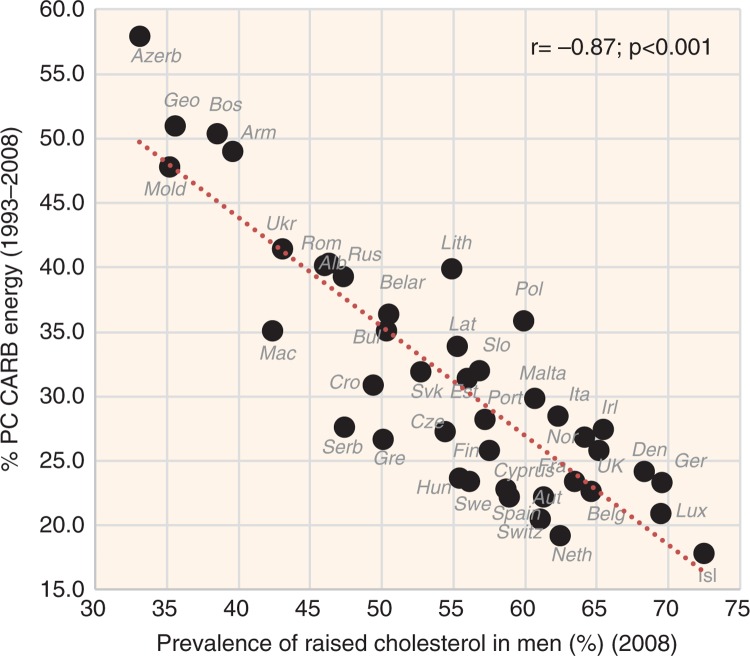
Correlation between the mean proportion of energy from potato and cereal carbohydrates (% PC CARB energy) and the prevalence of raised cholesterol levels in men (*r*=−0.87; *p*<0.001).

**Table 1:** Results of the Pearson linear correlations (selected variables)

	Raised blood cholesterol (%)	Raised blood cholesterol (%)	Raised blood pressure (%)	Raised blood pressure (%)	Raised fasting blood glucose (%)	Raised fasting blood glucose (%)	Actual CHD mortality (all ages)	Actual CHD mortality (all ages)	Actual total CVD mortality (all ages)	Actual total CVD mortality (all ages)

	Men	Women	Men	Women	Men	Women	Men	Women	Men	Women
Fruits total	0.58	0.52	−0.68	−0.69	−0.42	−0.64	−0.64	−0.64	−0.77	−0.71
Bananas	0.74	0.68	−0.42	−0.60	−0.24	−0.58	−0.44	−0.47	−0.68	−0.72
Oranges and mandarins	0.66	0.58	−0.66	−0.75	−0.38	−0.62	−0.55	−0.57	−0.74	−0.76
Alcoholic beverages	0.68	0.65	−0.30	−0.49	−0.44	−0.63	−0.36	−0.37	−0.52	−0.56
Beer	0.61	0.58	−0.19	−0.38	−0.31	−0.50	−0.27	−0.28	−0.41	−0.44
Dist. beverages	−0.08	−0.01	0.40	0.34	0.07	0.18	0.36	0.27	0.40	0.31
Wine	0.42	0.40	−0.48	−0.52	−0.45	−0.57	−0.45	−0.42	−0.56	−0.54
Coffee	0.70	0.66	−0.50	−0.67	−0.36	−0.68	−0.49	−0.51	−0.66	−0.69
Ref. sugar and sweeteners	0.67	0.65	−0.35	−0.56	−0.33	−0.53	−0.04	−0.08	−0.31	−0.44
Refined sugar	0.57	0.54	−0.33	−0.53	−0.39	−0.50	0.00	−0.06	−0.26	−0.40
Oil crops total	0.16	0.05	−0.50	−0.38	−0.18	−0.27	−0.36	−0.36	−0.37	−0.30
Tree nuts	0.27	0.18	−0.65	−0.58	−0.35	−0.46	−0.50	−0.50	−0.59	−0.54
Plant oils total	0.52	0.45	−0.56	−0.57	−0.48	−0.61	−0.44	−0.46	−0.55	−0.56
Olive oil	0.12	0.06	−0.45	−0.32	−0.11	−0.18	−0.28	−0.29	−0.35	−0.31
Sunflower oil	−0.35	−0.30	0.20	0.29	0.10	0.22	0.20	0.21	0.43	0.46
Cereals total	−0.74	−0.73	0.42	0.65	0.44	0.70	0.32	0.37	0.54	0.61
Potatoes	0.08	0.12	0.32	0.20	−0.15	0.08	0.56	0.45	0.35	0.15
Legumes total	−0.03	−0.02	−0.08	0.05	0.05	−0.05	−0.29	−0.27	−0.13	−0.02
Vegetables total	−0.29	−0.35	−0.21	0.09	0.09	0.15	−0.13	−0.11	0.01	0.13
Onions	−0.54	−0.58	0.20	0.43	0.18	0.47	0.26	0.30	0.42	0.50
Plant protein	−0.53	−0.54	0.19	0.47	0.32	0.49	0.12	0.14	0.32	0.39
Plant fat	0.58	0.50	−0.64	−0.65	−0.45	−0.63	−0.50	−0.53	−0.63	−0.63
Meat total	0.83	0.79	−0.54	−0.73	−0.50	−0.76	−0.43	−0.49	−0.65	−0.73
Meat protein	0.84	0.80	−0.57	−0.76	−0.55	−0.79	−0.44	−0.50	−0.66	−0.74
Meat fat	0.78	0.81	−0.47	−0.71	−0.49	−0.78	−0.40	−0.46	−0.61	−0.70
Beef and pork fat	0.70	0.74	−0.34	−0.59	−0.50	−0.74	−0.32	−0.38	−0.53	−0.62
Dairy total	0.62	0.56	−0.42	−0.62	−0.59	−0.75	−0.37	−0.42	−0.58	−0.66
Milk	−0.19	−0.16	0.26	0.22	0.03	0.13	0.19	0.17	0.21	0.19
Cheese	0.71	0.64	−0.70	−0.79	−0.52	−0.75	−0.51	−0.53	−0.69	−0.72
Dairy protein	0.63	0.57	−0.45	−0.64	−0.51	−0.68	−0.35	−0.40	−0.55	−0.62
Dairy fat	0.54	0.48	−0.50	−0.62	−0.47	−0.63	−0.40	−0.43	−0.54	−0.57
Animal protein	0.89	0.83	−0.60	−0.82	−0.53	−0.79	−0.41	−0.48	−0.68	−0.78
Animal fat	0.89	0.87	−0.51	−0.76	−0.57	−0.85	−0.40	−0.46	−0.64	−0.73
Animal fat and anim. protein	0.92	0.88	−0.57	−0.82	−0.57	−0.85	−0.42	−0.48	−0.68	−0.78
Total protein	0.76	0.70	−0.59	−0.72	−0.45	−0.67	−0.41	−0.47	−0.62	−0.71
Total fat	0.86	0.81	−0.66	−0.82	−0.59	−0.86	−0.51	−0.56	−0.73	−0.79
Total fat and anim. protein	0.90	0.84	−0.66	−0.85	−0.59	−0.86	−0.49	−0.55	−0.73	−0.81
Total fat and total protein	0.88	0.82	−0.67	−0.83	−0.58	−0.85	−0.51	−0.57	−0.73	−0.81
% CA energy	−0.85	−0.81	0.62	0.81	0.52	0.84	0.52	0.58	0.72	0.77
% PC carb energy	−0.87	−0.83	0.58	0.79	0.51	0.83	0.46	0.51	0.68	0.74
% Plant food energy	−0.87	−0.87	0.39	0.69	0.50	0.79	0.33	0.39	0.57	0.67
Total energy	0.77	0.72	−0.56	−0.68	−0.56	−0.74	−0.37	−0.44	−0.59	−0.68
Raised blood pressure (men)	−0.55				0.37		0.60		0.69	
Raised blood pressure (women)		−0.70				0.77		0.59		0.80
Smoking (men)	−0.62		0.48		0.34		0.53		0.67	
Smoking (women)		0.47		−0.37		−0.56		−0.59		−0.46
BMI (men)	0.59		−0.18		0.09		−0.40		−0.46	
BMI (women)		−0.37		0.47		0.68		0.49		0.43
Health expenditure (2008)	0.83	0.75	−0.69	−0.85	−0.50	−0.79	−0.56	−0.58	−0.79	−0.82
Health expenditure (1995–2008)	0.83	0.75	−0.72	−0.87	−0.49	−0.79	−0.57	−0.59	−0.79	−0.82

For more details, see Supplementary Dataset Sheet 5.
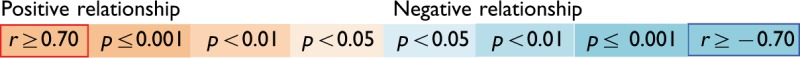

CA energy=energy from carbohydrates and alcohol; PC CARB energy=energy from potato and cereal carbohydrates.

**Fig. 3:**
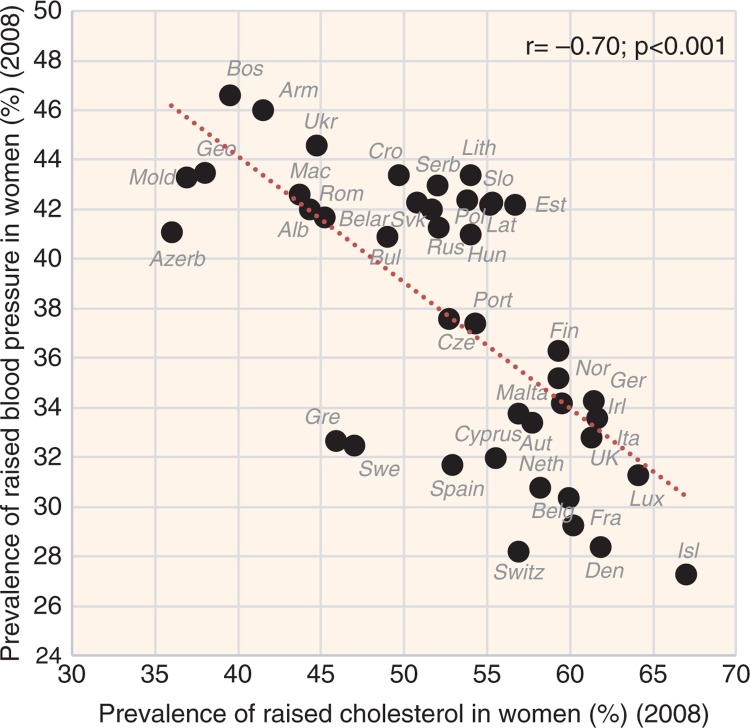
Correlation between the prevalence of raised blood pressure and the prevalence of raised cholesterol levels in women (*r*=−0.70; *p*<0.001).

**Fig. 4:**
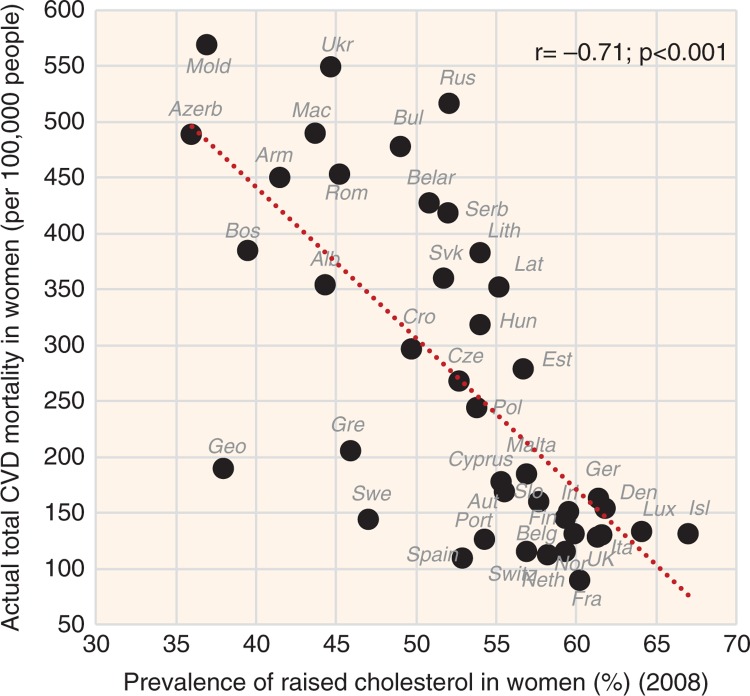
Correlation between the actual CVD mortality and the prevalence of raised cholesterol levels in women (*r*=−0.71; *p*<0.001).

Low cholesterol levels correlate most strongly with the proportion of plant food energy in the diet (*r*=−0.87, *p*<0.001 in both sexes) and with sources of plant carbohydrates represented by items such as % PC CARB energy (*r*=−0.87 in men, *r*=−0.83 in women; *p*<0.001) (Fig. 2), % CA energy (*r*=−0.85 in men, *r*=−0.81 in women; *p*<0.001), and cereals (*r*=−0.74 in men, *r*=−0.73 in women; *p*<0.001). Smoking correlates quite strongly with lower cholesterol as well, but in men only (*r*=−0.62, *p*<0.001). Remarkably, the relationship of raised cholesterol with CVD risk is always negative, especially in the case of total CVD mortality (*r*=−0.69 in men, *r*=−0.71 in women; *p*<0.001) (Figs. 3 and 4, Supplementary Figs. 1 and 2).

**Fig. 5:**
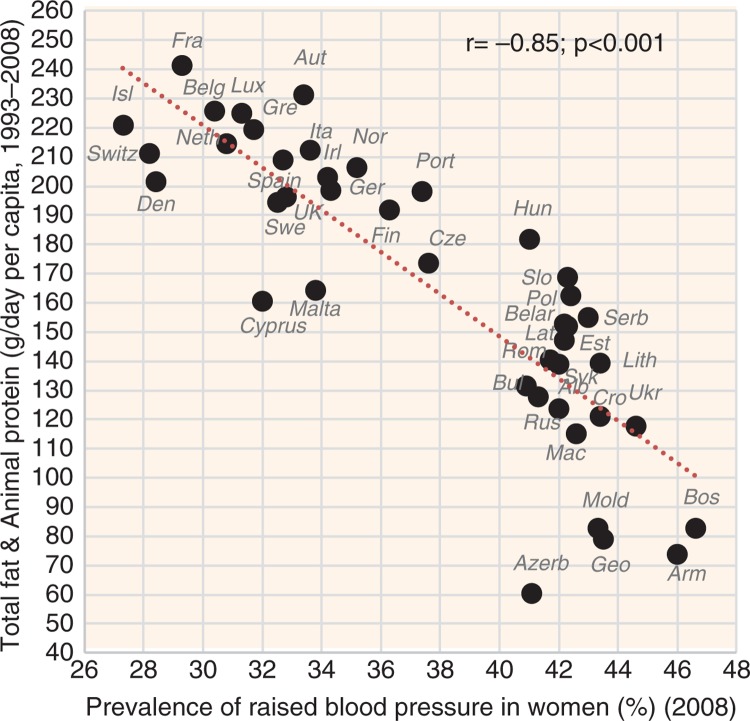
Correlation between the mean daily consumption of total fat and animal protein and the prevalence of raised blood pressure in women (*r*=−0.85; *p*<0.001).

**Fig. 6:**
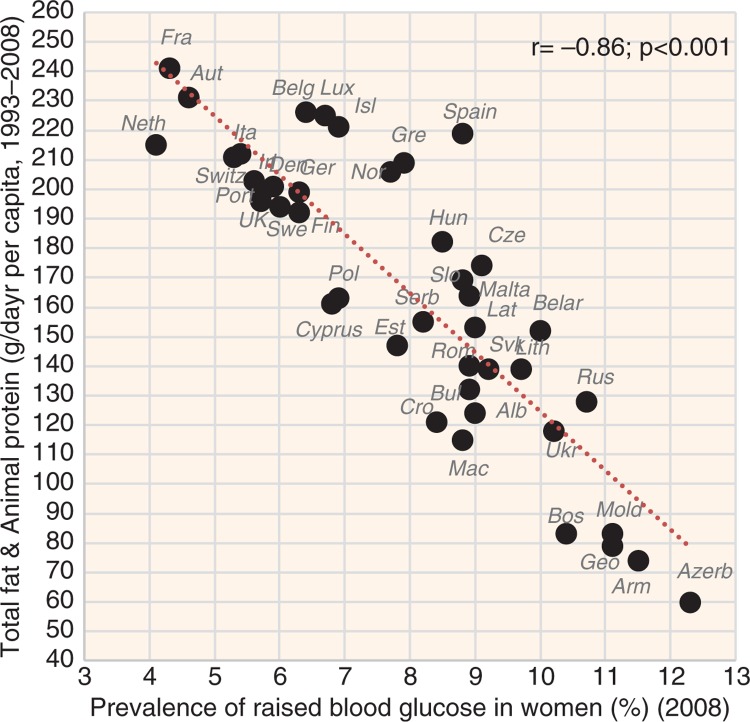
Correlation between the mean daily consumption of total fat and animal protein and the prevalence of raised blood glucose in women (*r*=−0.86; *p*<0.001).

**Fig. 7:**
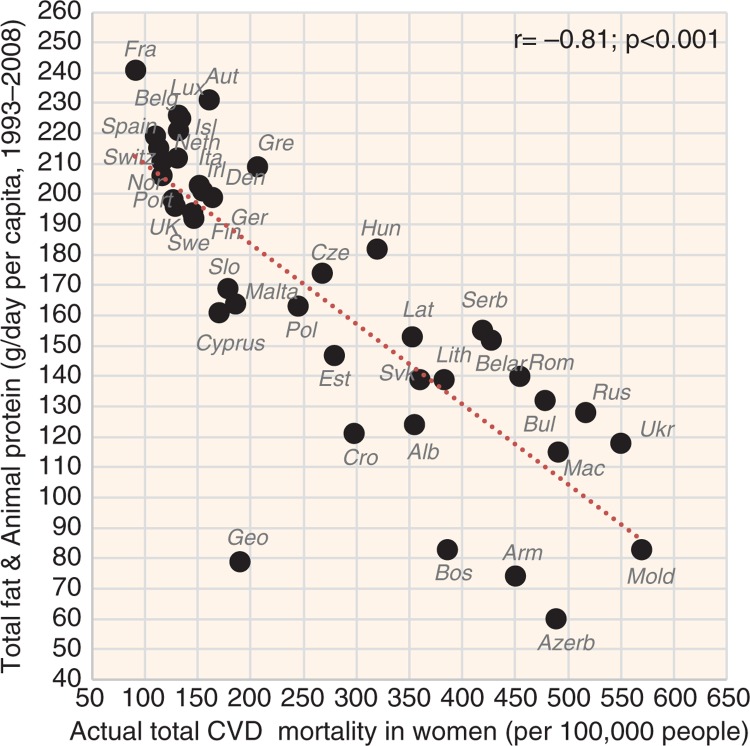
Correlation between the mean daily consumption of total fat and animal protein and the actual total CVD mortality in women (*r*=−0.81; *p*<0.001).

The indicators of CVD prevalence (raised blood pressure, raised fasting blood glucose) and CVD incidence (CHD mortality, total mortality) generally have the highest negative correlation with health expenditure and with total fat and animal protein (or total fat and total protein). Total fat and animal protein correlates particularly strongly with raised blood pressure (*r*=−0.85) (Fig. 5), raised blood glucose (*r*=−0.86) (Fig. 6), and total CVD mortality (*r*=−0.81, *p*<0.001) (Fig. 7) in women. Health expenditure (1993–2008) reaches similarly high correlations with raised blood pressure (*r*=−0.87, *p*<0.001 in women) and total CVD mortality (*r*=−0.82, *p*<0.001 in women), but not with raised blood glucose (*r*=−0.79, *p*<0.001 in women). These variables are also the most significant correlates of high life expectancy: total fat and animal/total protein in women (*r*=0.85, *p*<0.001) and health expenditure (1993–2008) in men (*r*=0.81, *p*<0.001). The list of individual food items with the highest negative *r* values includes meat (especially meat protein), dairy (cheese), and fruits (oranges and mandarins) (Supplementary Figs. 3–12). Fruits are the main negative correlate of CHD mortality.

**Fig. 8:**
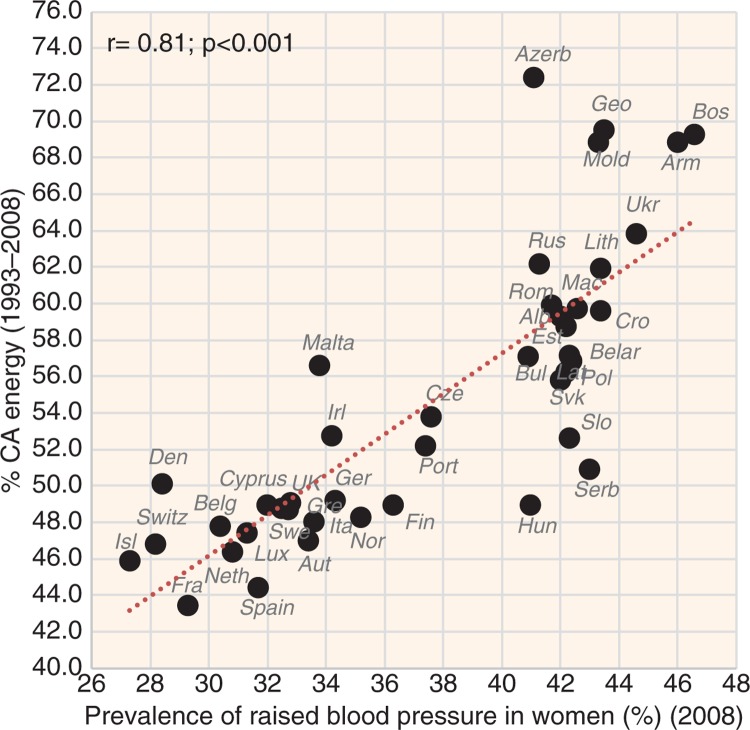
Correlation between the mean proportion of energy from carbohydrates and alcohol (% CA energy) and the prevalence of raised blood pressure in women (*r*=0.81; *p*<0.001).

**Fig. 9:**
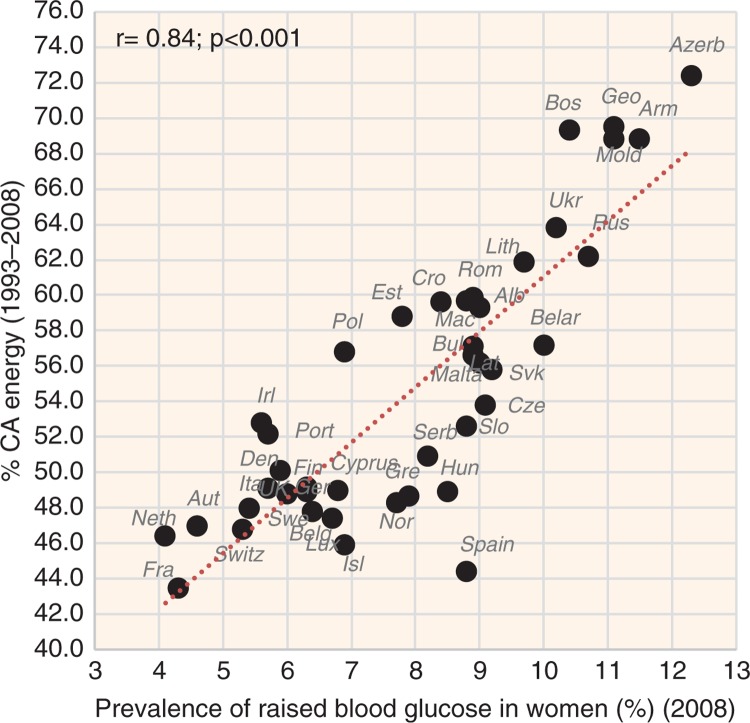
Correlation between the mean proportion of energy from carbohydrates and alcohol (% CA energy) and the prevalence of raised blood glucose in women (*r*=0.84; *p*<0.001).

**Fig. 10:**
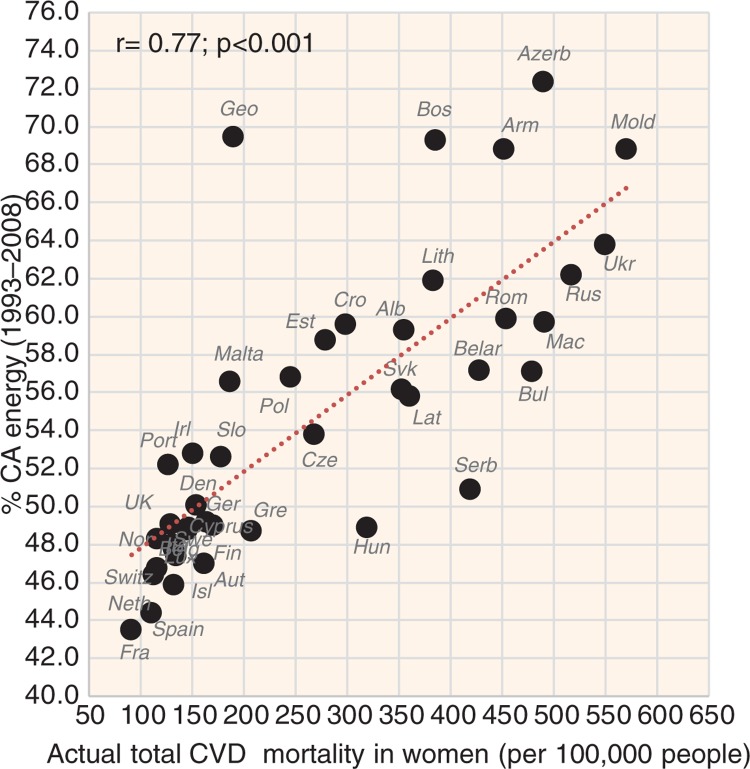
Correlation between the mean proportion of energy from carbohydrates and alcohol (% CA energy) and the actual total CVD mortality in women (*r*=0.77; *p*<0.001).

The most significant positive correlate of CVD risk is the proportion of CA energy, especially in the case of raised blood pressure (*r*=0.62 in men, *r*=0.81 in women; *p*<0.001) (Fig. 8), raised blood glucose (*r*=0.52 in men, *r*=0.84 in women; *p*<0.001) (Fig. 9), and total CVD mortality (*r*=0.72 in men, *r*=0.77 in women; *p*<0.001) (Fig. 10). This factor also strongly correlates with low life expectancy (*r*=−0.76 in men, *r*=−0.83 in women; *p*<0.001). The highest life expectancy is tied with ~45–50% CA energy intake. The proportion of PC CARB energy and plant food energy has a high positive correlation with CVD risk as well. The number of individual foodstuffs positively correlating with CVD risk is remarkably limited and consists of cereals, potatoes, distilled beverages, sunflower oil and onions (Supplementary Figs. 15–22). The most consistent relationship with CVD risk can be found in cereals, especially with raised blood glucose (*r*=0.44, *p*=0.004 in men; *r*=0.70, *p*<0.001 in women) and with total CVD mortality (*r*=0.54 in men, *r*=0.61 in women, *p*<0.001). On the other hand, sunflower oil correlates only with total CVD mortality and potatoes come to the foreground as a predictor of CHD mortality.

**Fig. 11:**
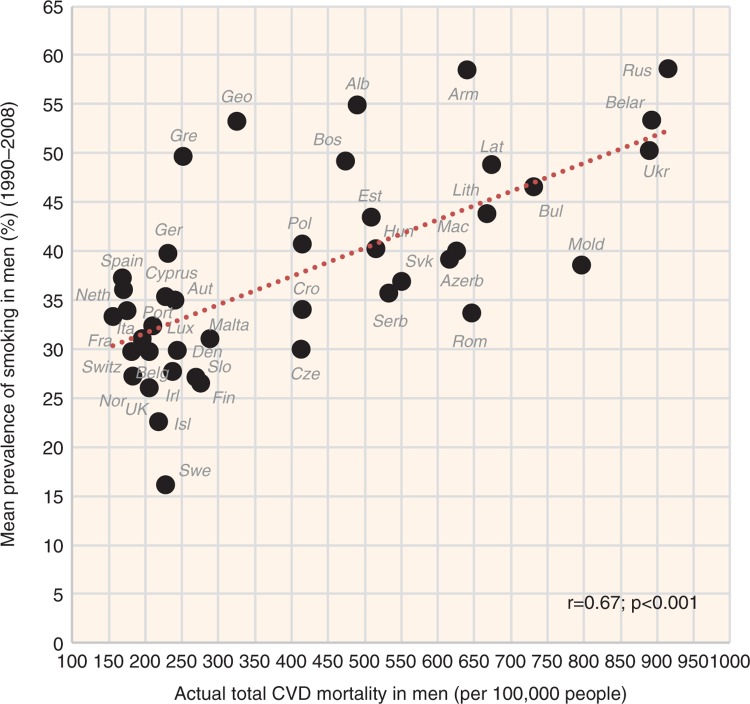
Correlation between the mean prevalence of smoking and the actual total CVD mortality in men (*r*=0.67; *p*<0.001).

**Fig. 12:**
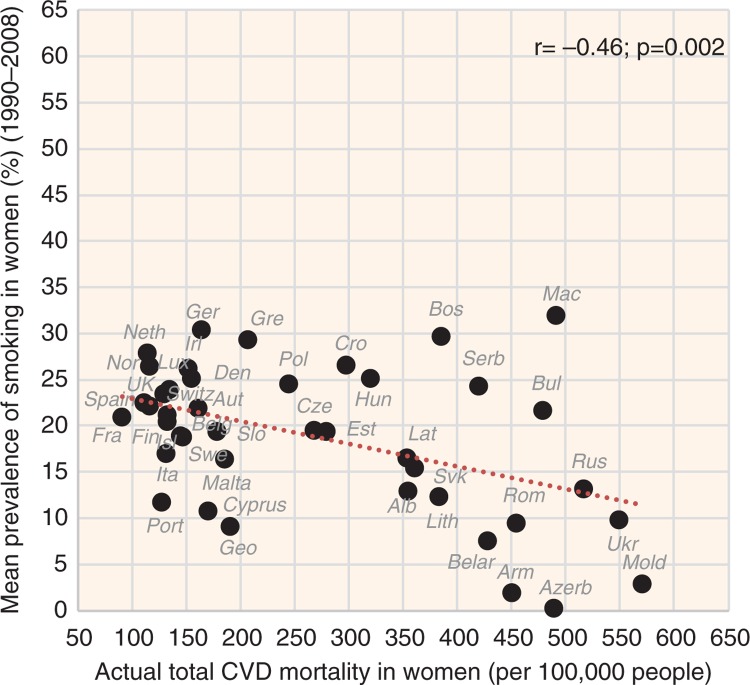
Correlation between the mean prevalence of smoking and the actual total CVD mortality in women (*r*=−0.46; *p*=0.002).

**Fig. 13:**
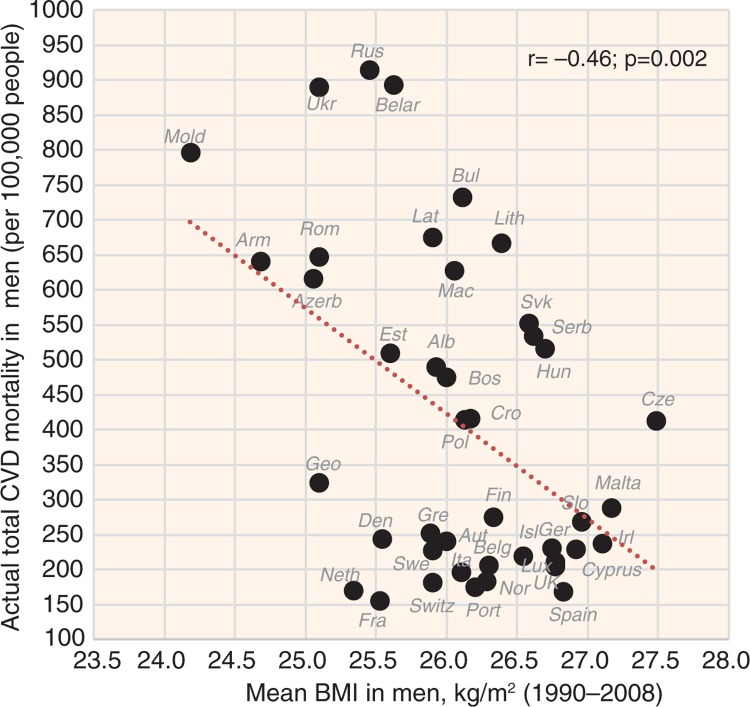
Correlation between the mean BMI and the actual CVD mortality in men (*r*=−0.46; *p*=0.002).

**Fig. 14:**
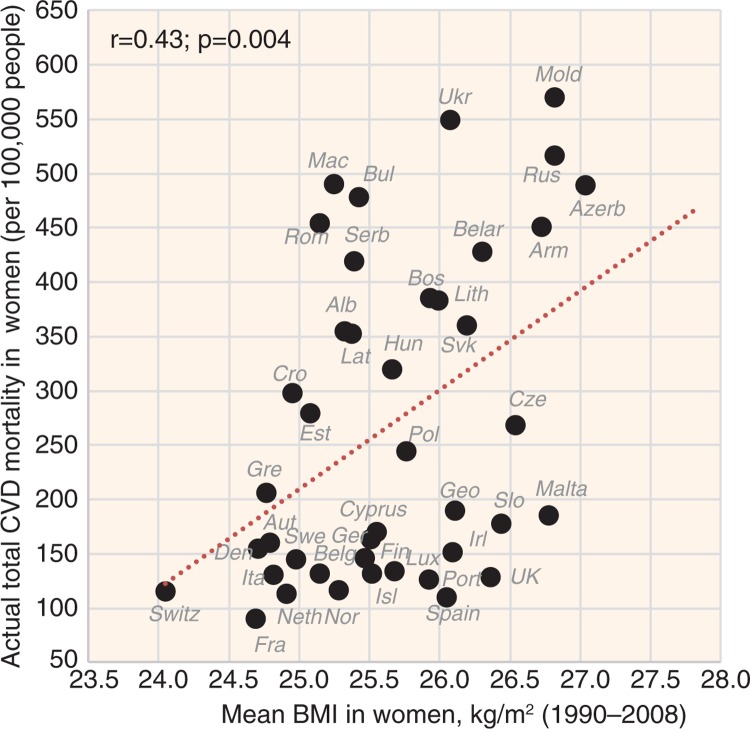
Correlation between the mean BMI and the actual CVD mortality in women (*r*=0.43; *p*=0.004).

A paradoxical relationship with CVD risk was found in smoking and BMI. Although smoking correlates positively with CVD risk in men (*r*=0.67, *p*<0.001 with total CVD mortality), it correlates negatively in women (Figs. 11 and 12). In contrast, BMI correlates positively with CVD risk in women and has the opposite relationship in men (Figs. 13 and 14).

### Factor analysis

**Fig. 15:**
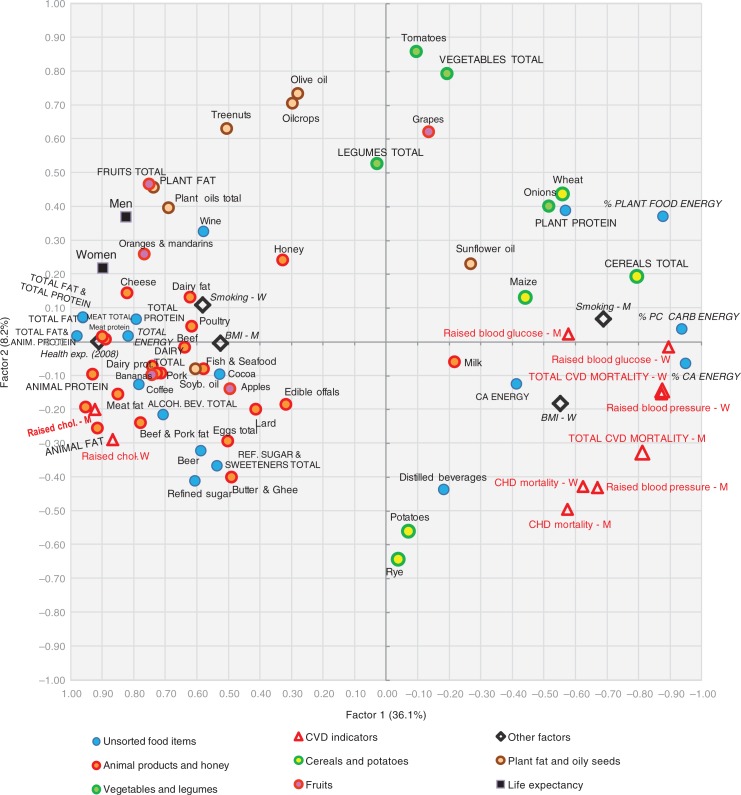
A plot of two principal components (Factor 1 and Factor 2) explaining 44.3% variability in the correlation between food consumption, BMI, smoking, health expenditure, and CVD indicators. For better clarity, some less important or too repetitive variables (fish and seafood fat, and potato and cereal energy) were omitted. CA energy=energy from carbohydrates and alcohol (kcal), PC CARB energy=energy from potato and cereal carbohydrates (kcal), BMI=body mass index, CVD=cardiovascular disease, CHD=coronary heart disease, Health exp. (2008)=health expenditure per capita for 2008, Raised chol.=raised cholesterol, M=men, W=women.

**Fig. 16:**
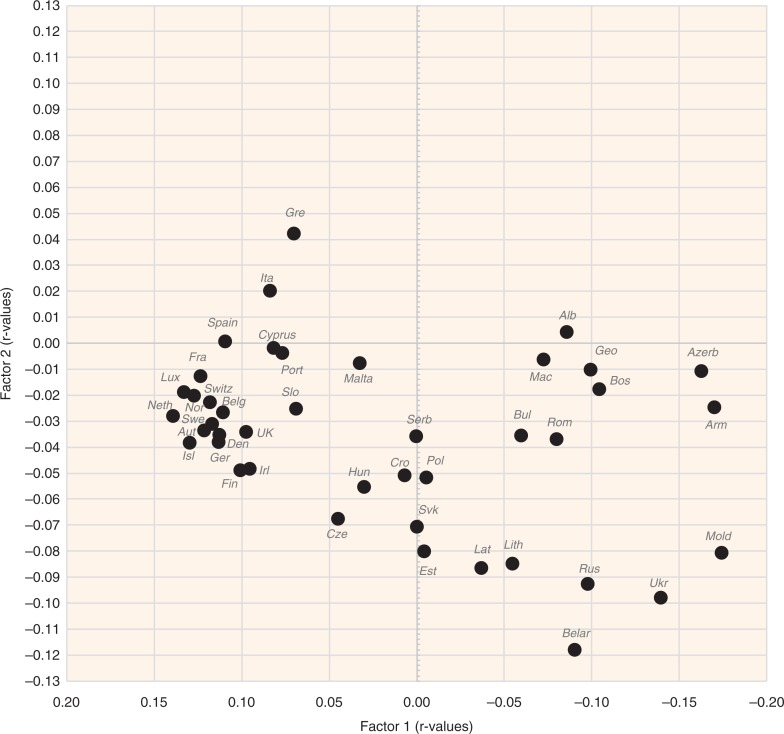
A summary correlation of Factor 1 and Factor 2 with the data of food consumption, BMI, smoking, health expenditure and CVD indicators in individual 42 European countries.

**Fig. 17:**
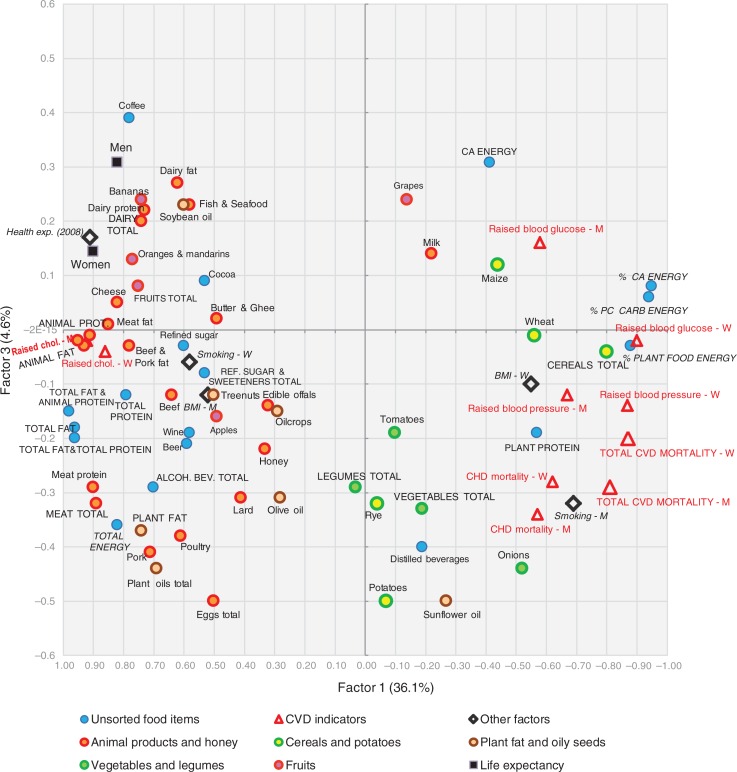
A plot of two principal components (Factor 1 and Factor 3) explaining 40.7% variability in the correlation between food consumption, BMI, smoking, health expenditure and CVD indicators. For better clarity, some less important or too repetitive variables (fish and seafood fat, and potato and cereal energy) were omitted. CA energy=energy from carbohydrates and alcohol (kcal), PC CARB energy=energy from potato and cereal carbohydrates (kcal), BMI=body mass index, CVD=cardiovascular diseases, CHD=coronary heart disease, Health exp. (2008)=health expenditure per capita for 2008, Raised chol.=raised cholesterol, M=men, W=women.

**Fig. 18:**
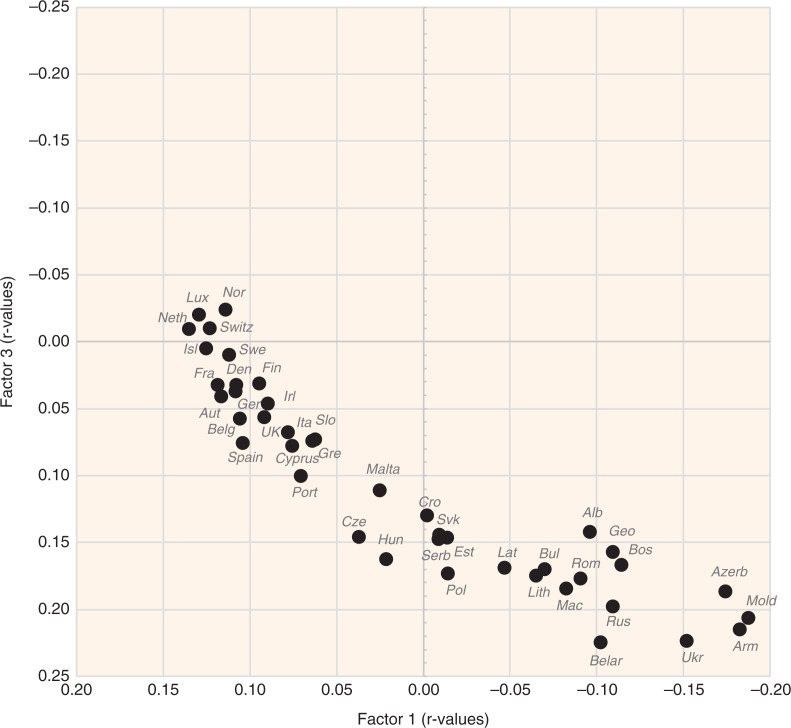
A summary correlation of Factor 1 and Factor 3 with the data of food consumption, BMI, smoking, health expenditure, and CVD indicators in individual 42 European countries.

A factor analysis of the examined variables is displayed in Figs. 15–18. The first two factors explain 36.1 and 8.2% variability, respectively (44.3% of total variability). The first factor separates variables that have the highest negative correlation with CVD risk (fat and protein consumption, health expenditure, animal products, alcoholic beverages, coffee, fruits) from those that are most closely associated with CVD risk (% CA energy, % PC CARB energy). This radical division corresponds with the dramatic differences between the living style and diet of Western and Northern Europe on the one hand (especially in the Netherlands, Luxembourg, and Iceland), and Eastern and Southeastern Europe on the other hand (Moldova, Armenia, Azerbaijan). Milk is the only animal product that deviates from this trend because in recent decades, it has become the main source of animal proteins in less developed countries, and on its own, it makes up only 8.7% of total fat and total protein intake.

The second factor separates the diets typical for the Mediterranean (mainly in Greece, Italy, and Albania) from the diets of Eastern Europe (Belarus, Ukraine, Russia). It is probably even more revealing because it highlights variables in the northwestern section of the plot that are in the strongest opposition against CVDs: tree nuts, fruits total, plant fat, plant oils, wine, oranges and mandarins, cheese, and dairy fat. This food composition also correlates with longevity and largely accords with the ‘Mediterranean’ dietary style (15). However, nowadays it can find close parallels even in many Western European countries, particularly in France and Luxembourg (Fig. 16).

The statistical power of Factor 3 is weak (4.6% variation), but it highlights the polarity between two other types of diet. Dairy products, coffee, fruits (bananas, oranges and mandarins), soybean oil, and fish and seafood are consumed in the wealthy countries of Northern and Northwestern Europe with high health expenses (Norway, Luxembourg, Switzerland, the Netherlands), whereas cereals, vegetables, potatoes, onions, distilled beverages, and sunflower oil are generally typical for the eastern half of Europe (Belarus, Ukraine, Armenia, Moldova) (Fig. 18). In this division, longevity and low CVD mortality are more tightly associated with health expenditure. Nevertheless, some of the variables that are highlighted by Factor 2 (fruits total, oranges and mandarins, cheese, dairy fat) appear in the strongest opposition against CVD risk again, which means that in a three-dimensional model, they would be the most distant from the indicators of CVDs.

The position of the potential CVD risk factors remains basically stable in both factor analyses, but onions and sunflower oil are notable exceptions. The consumption of onions is high in Mediterranean countries with low CVD mortality (Spain, Greece, Portugal) (Factor 2), but it is also high in Southeastern Europe (Romania, Armenia, Macedonia etc.) (Factor 3), where we find high CVD mortality. Similarly, the consumption of sunflower oil is high in Spain and France, but even in Bulgaria, Macedonia, and Romania.

### Regression analyses

Regression analyses were performed in raised blood pressure and total CVD mortality, in both sexes. The results of the best parsimonious models computed via the bootstrapping method are displayed in Supplementary Tables 3a, 4a, 5a, and 6a. Among top 10 variables with the highest *beta* coefficients in the ridge regression, health expenditure (both for 2008 and 1995–2008), cheese, and oranges and mandarins are selected three times, followed by tree nuts, which are selected twice. The LASSO regression highlighted only a few variables with non-zero *beta* coefficients. However, oranges and mandarins appeared three times in the best models. Total fat and animal protein and health expenditure (both for 2008 and 1995–2008) emerged in two models. In the elastic net regression, fruits, oranges and mandarins, and total fat appeared in all four models. Cheese, total fat and animal protein, and health expenditure (both for 2008 and 1995–2008) were identified three times. Total fat and total protein, tree nuts, total protein, % CA energy, and % PC CARB energy were identified twice. Therefore, these regression models produced results that are quite similar to the factor analysis. Health expenditure and oranges and mandarins were consistently highlighted by all three regression methods. Cheese, tree nuts, and the intake of fat and protein were selected by two regression methods.

The model of the elastic net regression computed without bootstrapping, based on the lowest prediction error (Supplementary Tables 3b, 4b, 5b and 6b), always included more variables and was less helpful, especially in the case of total CVD mortality in women, where 53 variables (albeit often with low *beta* coefficients) were selected. The only items that appeared in all four models included % CA energy, health expenditure, total fat and total protein, and total fat. Cheese, tree nuts, oranges and mandarins, and % PC CARB energy were selected three times.

### Temporal changes of correlation coefficients

**Fig. 19:**
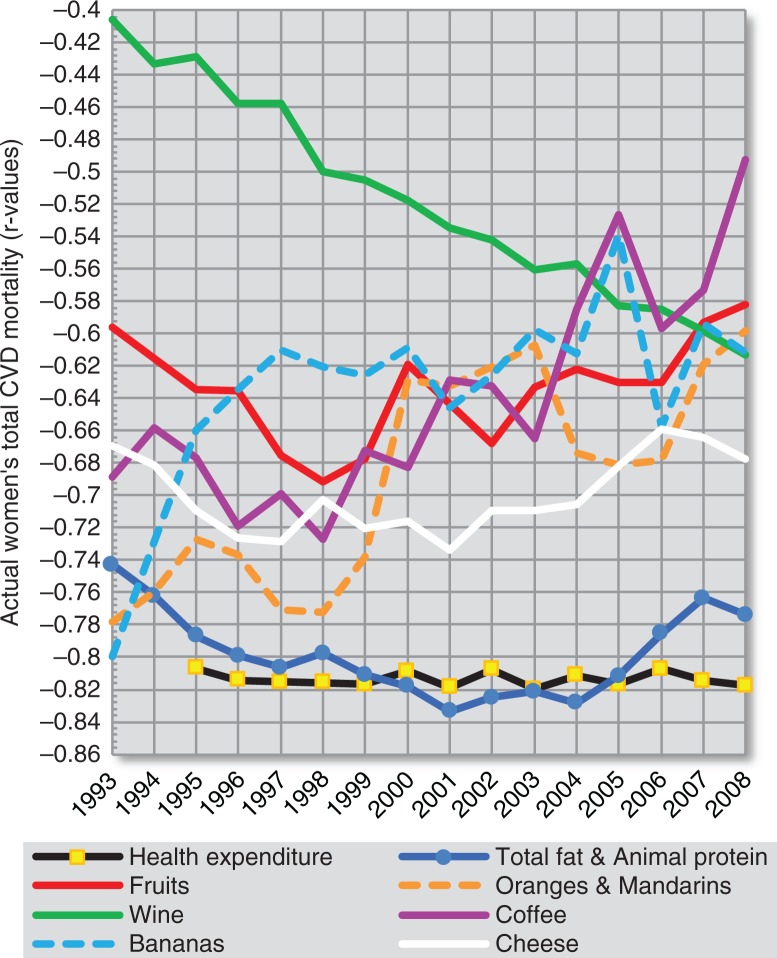
Temporal changes in the relationship among 8 negative correlates of total CVD mortality (women).

**Fig. 20:**
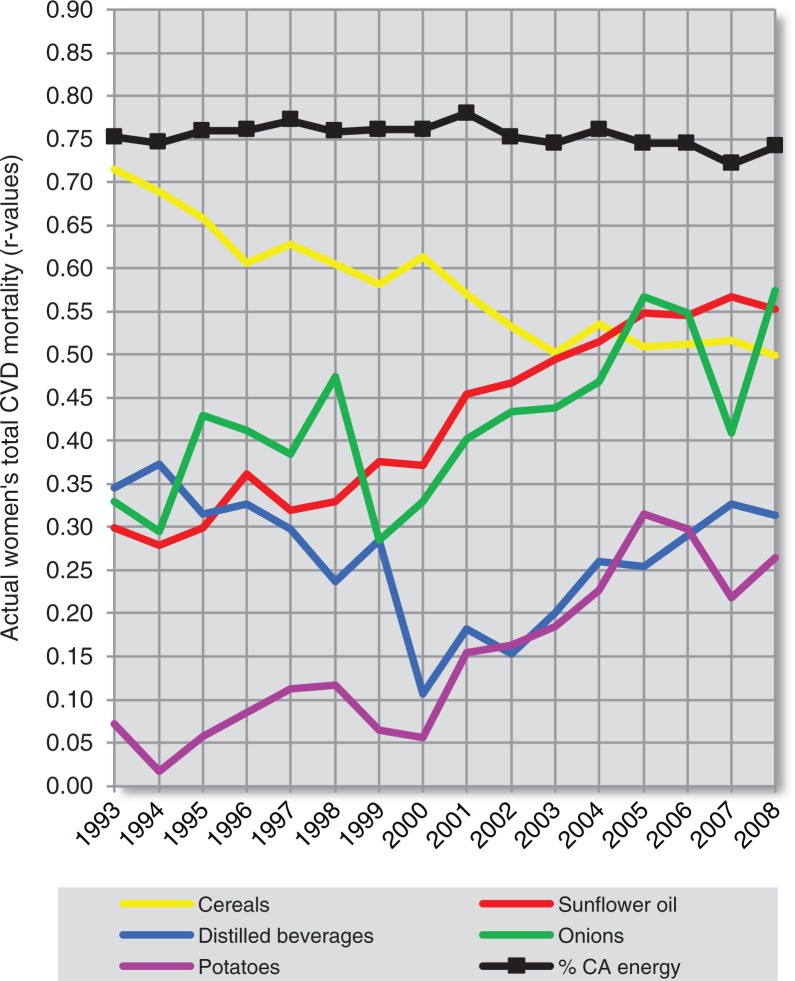
Temporal changes in the relationship among 6 positive correlates of total CVD mortality (women).

**Fig. 21:**
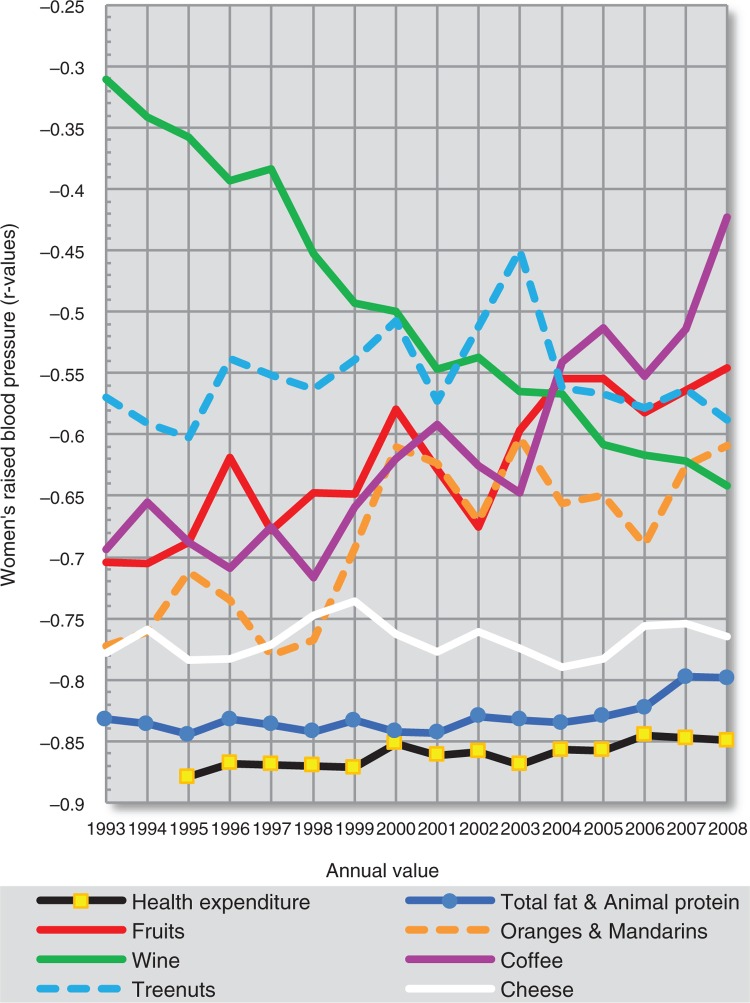
Temporal changes in the correlation among 8 negative correlates of raised blood pressure (women).

**Fig. 22:**
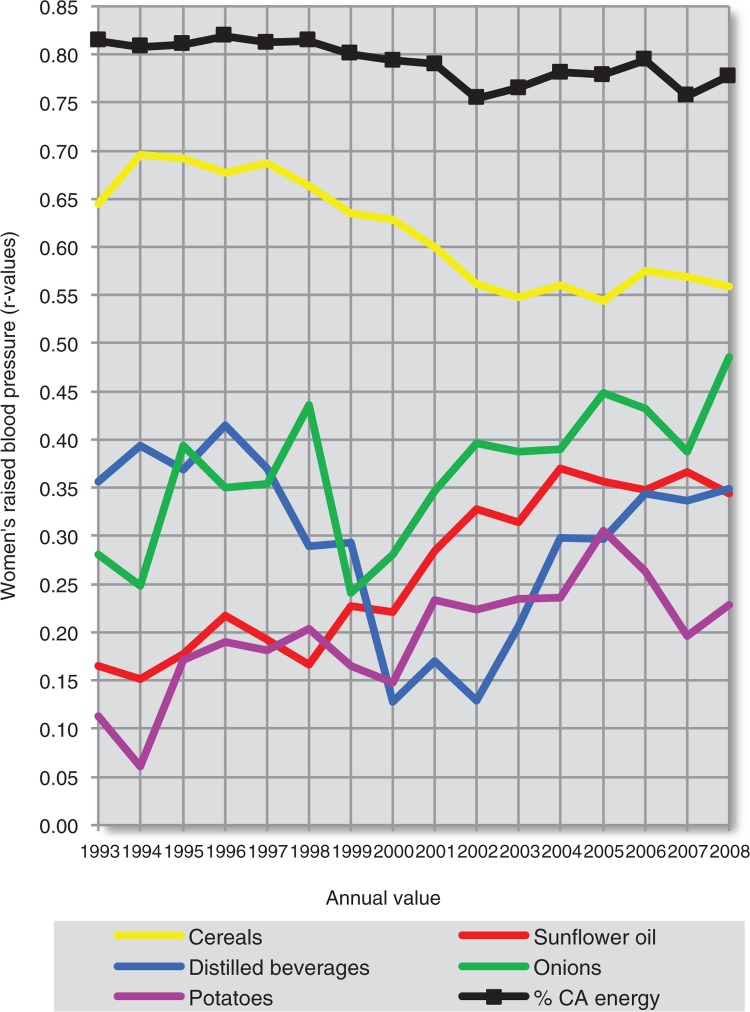
Temporal changes in the correlation among 6 positive correlates of raised blood pressure (women).

Considering that many examined variables are characterised by a strong degree of multicollinearity, it would be important to examine their temporal correlation with CVD indicators. This approach would be particularly important with regard to the possible influence of the major confounder – health expenditure. At the same time, it is possible that some of these temporal trends could reflect a long-term effect of a particular variable on CVD health (Figs. 19–22 and Supplementary Figs. 23–26).

Using the dependent *t*-test and comparing standard deviations of the mean differences in *r* values (Supplementary Tables 7–10), we can clearly see that out of all main negative correlates, health expenditure is most consistently related to total fat and animal protein, cheese, and fruits. It is also partly associated with bananas (in the case of total CVD mortality) and tree nuts (in the case of raised blood pressure). The linear slopes of these trends are mostly similar as well. Because bananas play a much weaker role among the correlates of raised blood pressure, they seem to be the most likely spurious correlate in the analysis. In contrast, tree nuts and cheese have a stronger correlation with CVD prevalence (raised blood pressure) than with CVD mortality (Supplementary Figs. 13 and 14), which suggests a relationship that is independent of healthcare. The same applies for total fat and animal protein and other indicators of fat and protein intake, but in women only. Cheese shows some temporal relationship with tree nuts in the comparison of raised blood pressure, but not with oranges and mandarins.

The relationship of health expenditure to other negatively correlated variables is less likely. Among them, we can clearly separate the highly independent trend line of wine. The trends in oranges and mandarins, and coffee tend to go in the opposite direction. The annual consumption of coffee has been significantly associated with fruits and oranges and mandarins in all 16 years, and especially with oranges and mandarins (*p*≤0.001). This can again indicate a spurious relationship to CVD indicators.

The plot of the positive correlates separates % CA energy and cereals from sunflower oil, onions and potatoes. The *r* values of % CA energy decreased throughout the 90s due to the unstable position of Georgia (compare Fig. 10 and Supplementary Figs. 17 and 21), but they otherwise follow those of cereals, which is understandable because cereals are the main source of carbohydrates in the diet. The *r* values of % CA energy and cereals generally rise with increasing time. This picture would accord with the chronic nature of CVDs, where the consequences of food intake manifest after several decades.

The trends of sunflower oil, onions, and potatoes are completely different and appear to be cumulative. They are also mutually similar, particularly in the plot of total CVD mortality. Despite that, sunflower oil intake significantly correlates only with onions – in 13 out of 16 years (*p*<0.05). It does not correlate with potatoes in a single year. Potatoes were associated with onions only weakly in 2 years (2004 and 2008). This suggests that the origin of this similarity is only spurious. Alternatively, all of these three variables are associated with some unknown confounder. Indeed, they cluster in the factor analysis (Fig. 17), together with distilled beverages, and are negative dietary correlates of some food items with potentially preventive effects such as fruits and dairy. The fact that sunflower oil and onions significantly correlate with mortality, but much less with raised blood pressure, also indicates that they may be casually associated with some factors related to mortality. Distilled beverages are significantly positively associated only with potatoes (in 12 years) and the trend line of *r* values is markedly U-shaped. In contrast with sunflower oil and onions, the correlation of distilled alcohol and potatoes with raised blood pressure and total CVD mortality is not very different.

### Historical stability

The last tool that we used for the assessment of the validity of our results was a historical comparison of CVD statistics (mean blood pressure, CHD mortality, stroke mortality, and total CHD and stroke mortality) from 1980, 1990, and 2000, with the mean food consumption in the preceding 16 years. (For example, CVD statistics from 1980 were compared with the mean food consumption from the period 1965–1980) (see Supplementary Dataset, Sheet 4). A limiting factor of this analysis was a smaller number of useable countries. The data of mean blood pressure were available for all 42 countries, but the less complete statistics of CHD and stroke mortality, and food consumption reduced this number to 16 for 1980, 21 for 1990, and 24 for 2000 (Tables 2 and 3).

**Table 2:** Historical comparison: correlations between food consumption (a mean of 16 preceding years) and men's statistics of cardiovascular diseases between 1980 and 2000

Countries (*n*)	Mean blood pressure			CHD mortality			Stroke mortality			CHD and stroke mortality total		
			
	1980	1990	2000	1980	1990	2000	1980	1990	2000	1980	1990	2000

	16	21	24	16	21	24	16	21	24	16	21	24
Fruits total	−0.20	−0.26	−0.43	−0.40	−0.36	−0.57	−0.06	−0.32	−0.51	−0.39	−0.40	−0.57
Bananas	0.62	−0.05	0.09	−0.02	−0.31	−0.52	−0.63	−0.70	−0.69	−0.26	−0.52	−0.62
Oranges and mandarins	0.03	−0.49	−0.51	−0.41	−0.49	−0.67	−0.61	−0.70	−0.67	−0.62	−0.65	−0.70
Distilled beverages	−0.04	0.47	0.52	−0.03	0.46	0.50	0.74	0.69	0.55	0.26	0.62	0.55
Wine	0.10	−0.03	−0.15	−0.70	−0.55	−0.33	0.12	0.04	−0.06	−0.60	−0.38	−0.23
Coffee	0.33	0.05	−0.08	0.06	−0.06	−0.34	−0.55	−0.46	−0.50	−0.17	−0.24	−0.42
Cereals total	−0.59	−0.03	0.02	−0.13	0.13	0.39	0.68	0.64	0.60	0.15	0.36	0.50
Sunflower oil	−0.39	0.07	0.03	−0.27	−0.08	0.40	0.62	0.74	0.70	0.00	0.25	0.55
Potatoes	0.26	0.21	0.30	0.00	0.19	0.13	−0.24	−0.21	−0.08	−0.10	0.05	0.05
Vegetables total	−0.56	−0.41	−0.40	−0.69	−0.29	−0.02	0.12	0.19	0.22	−0.59	−0.13	0.09
Onions	−0.50	−0.22	−0.24	−0.50	−0.01	0.35	0.20	0.29	0.46	−0.38	0.11	0.41
Plant fat	−0.39	−0.50	−0.35	−0.60	−0.61	−0.42	−0.19	−0.27	−0.31	−0.63	−0.55	−0.39
Dairy total	0.52	0.04	−0.01	0.44	0.18	−0.18	−0.67	−0.48	−0.46	0.14	−0.07	−0.31
Meat total	0.49	0.23	−0.04	0.34	0.16	−0.12	−0.20	−0.03	−0.25	0.23	0.10	−0.19
Animal protein	0.55	−0.07	−0.19	0.36	0.04	−0.35	−0.66	−0.49	−0.58	0.08	−0.18	−0.47
Animal fat	0.57	0.45	0.19	0.63	0.44	−0.06	−0.38	−0.22	−0.36	0.43	0.23	−0.19
Anim. fat and anim. protein	0.59	0.26	0.05	0.56	0.30	−0.18	−0.51	−0.34	−0.48	0.31	0.07	−0.32
Total protein	0.15	−0.20	−0.30	0.31	0.08	−0.23	−0.36	−0.18	−0.38	0.14	−0.02	−0.30
Total fat	0.47	0.13	−0.09	0.37	0.05	−0.34	−0.69	−0.43	−0.52	0.07	−0.15	−0.44
Total fat and anim. protein	0.54	0.05	−0.13	0.39	0.05	−0.37	−0.73	−0.48	−0.58	0.08	−0.17	−0.48
Total fat and total protein	0.43	0.03	−0.16	0.41	0.06	−0.34	−0.68	−0.39	−0.53	0.11	−0.12	−0.44
CA energy	−0.35	0.14	0.24	−0.10	0.26	0.44	0.70	0.69	0.47	0.19	0.48	0.47
% CA energy	−0.44	−0.01	0.19	−0.29	0.05	0.45	0.78	0.60	0.63	0.04	0.29	0.55
Stroke mortality	−0.24	0.44	0.26	−0.01	0.49	0.81				0.38	0.77	0.93
Mean blood pressure				0.32	0.64	0.41	−0.24	0.44	0.26	0.20	0.65	0.36
Life expectancy (men)	0.16	−0.53	−0.37	0.01	−0.57	−0.82	−0.72	−0.86	−0.93	−0.28	−0.77	−0.91
GDP per capita by PPP		−0.08	−0.08		−0.29	−0.58		−0.74	−0.80		−0.52	−0.70

**Table 3:** Historical comparison: correlations between food consumption (a mean of 16 preceding years) and women's statistics of cardiovascular diseases between 1980 and 2000

Countries (*n*)	Mean blood pressure			CHD mortality			Stroke mortality			CHD and stroke mortality total		
			
	1980	1990	2000	1980	1990	2000	1980	1990	2000	1980	1990	2000

	16	21	24	16	21	24	16	21	24	16	21	24
Fruits total	0.00	−0.27	−0.46	−0.36	−0.45	−0.64	−0.16	−0.44	−0.64	−0.34	−0.50	−0.66
Bananas	−0.50	−0.38	−0.25	−0.11	−0.35	−0.51	−0.70	−0.76	−0.70	−0.45	−0.63	−0.63
Oranges and mandarins	−0.35	−0.59	−0.57	−0.48	−0.59	−0.70	−0.67	−0.75	−0.72	−0.70	−0.76	−0.74
Distilled beverages	0.48	0.51	0.49	0.05	0.38	0.36	0.73	0.62	0.45	0.42	0.56	0.42
Wine	0.20	−0.08	−0.26	−0.56	−0.50	−0.36	−0.02	−0.06	−0.20	−0.41	−0.32	−0.28
Coffee	−0.44	−0.27	−0.40	−0.31	−0.25	−0.38	−0.58	−0.55	−0.51	−0.53	−0.45	−0.46
Cereals total	0.71	0.41	0.47	0.15	0.21	0.45	0.69	0.70	0.65	0.47	0.51	0.57
Sunflower oil	0.33	0.27	0.26	−0.03	0.01	0.42	0.65	0.71	0.62	0.32	0.40	0.54
Potatoes	−0.02	0.04	0.16	−0.13	−0.01	−0.05	−0.17	−0.30	−0.13	−0.18	−0.17	−0.09
Vegetables total	0.25	−0.03	0.03	−0.42	−0.22	−0.06	0.04	0.20	0.12	−0.28	−0.01	0.03
Onions	0.19	0.00	0.17	−0.36	0.04	0.31	0.25	0.28	0.42	−0.12	0.18	0.38
Plant fat	−0.06	−0.32	−0.30	−0.44	−0.56	−0.53	−0.24	−0.32	−0.46	−0.44	−0.50	−0.51
Dairy total	−0.41	−0.37	−0.32	0.04	−0.03	−0.31	−0.56	−0.54	−0.43	−0.27	−0.32	−0.38
Meat total	−0.34	−0.19	−0.41	0.12	0.13	−0.29	−0.23	−0.16	−0.40	−0.04	−0.02	−0.36
Animal protein	−0.50	−0.59	−0.67	−0.09	−0.04	−0.46	−0.59	−0.51	−0.60	−0.38	−0.31	−0.55
Animal fat	−0.41	−0.08	−0.30	0.30	0.28	−0.20	−0.33	−0.32	−0.41	0.04	−0.02	−0.32
Anim. fat and anim. protein	−0.47	−0.30	−0.48	0.16	0.16	−0.32	−0.45	−0.42	−0.52	−0.12	−0.14	−0.44
Total protein	−0.12	−0.54	−0.56	−0.02	0.05	−0.33	−0.27	−0.17	−0.39	−0.16	−0.07	−0.37
Total fat	−0.62	−0.32	−0.47	0.05	−0.10	−0.54	−0.66	−0.58	−0.67	−0.32	−0.38	−0.63
Total fat and anim. protein	−0.61	−0.46	−0.57	−0.01	−0.08	−0.55	−0.68	−0.58	−0.69	−0.37	−0.37	−0.64
Total fat and total protein	−0.53	−0.43	−0.54	0.03	−0.05	−0.53	−0.63	−0.49	−0.65	−0.31	−0.31	−0.61
CA energy	0.73	0.46	0.47	0.19	0.33	0.39	0.69	0.72	0.46	0.50	0.59	0.44
% CA energy	0.73	0.47	0.61	0.08	0.17	0.60	0.75	0.70	0.74	0.46	0.49	0.70
Stroke mortality	0.40	0.53	0.55	0.28	0.57	0.87				0.73	0.88	0.97
Mean blood pressure				−0.04	0.42	0.55	0.40	0.53	0.55	0.19	0.54	0.57
Life expectancy (women)	−0.48	−0.64	−0.60	−0.44	−0.63	−0.85	−0.81	−0.89	−0.92	−0.74	−0.86	−0.92
GDP per capita by PPP		−0.47	−0.55		−0.38	−0.64		−0.80	−0.82		−0.67	−0.76

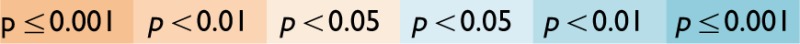

Some missing data of CVD statistics were supplemented by values from the previous or following year. The data of GDP per capita (by purchasing power parity) and life expectancy were taken from the World Bank (www.worldbank.org/). The value of GDP for Hungary (for 1990) was taken from the year 1991.

We found that stroke mortality showed a stable historical relationship with many food items in both sexes, and these relationships are in accord with the analysis of the present-day statistics. The common denominators of high stroke mortality were cereals, sunflower oil, distilled beverages, and CA energy. The common predictors of low stroke mortality in all the examined years were bananas, oranges and mandarins, coffee, dairy, animal protein, and total fat and animal protein. Stroke mortality correlated highly negatively with life expectancy and GDP per capita.

The trends in mean blood pressure and CHD mortality were different. Although the results in men from 2000, and in women from 1990 and 2000, were basically very similar such as in stroke mortality, the *r* values were mostly much lower. In other years we observed either very few significant correlations or very eccentric results, which went in the opposite direction than in stroke mortality and in the contemporary statistics. More concretely, men's mean blood pressure and CHD mortality from 1980 correlated *positively* with animal fat and animal protein, and showed no negative relationship with life expectancy. In addition, lower blood pressure in men was most strongly predicted by cereals, which again contradicts the contemporary statistics, in which cereals correlate positively with raised blood pressure. These tendencies largely persisted even in 1990. At the same time, the trends in women's mean blood pressure from 1980 were completely opposite than in men, and in the case of women's CHD mortality, only one significant *r* value (in wine) could be found. No variable correlated with CHD mortality consistently in both sexes, but plant fat did so five times, and wine and oranges and mandarins four times.

Apparently, the men's statistics from 1980 and 1990 pose a fundamental problem of the historical analysis. The key problem is why the *r* coefficients were different from those of women and why they began to reverse in the following years. The statistics of total CHD and stroke mortality, which should better represent total CVD mortality, remained roughly in between. In 1980, they still correlated weakly positively with animal fat in men (*r*=0.43, *p*=0.10), but in 2000, the trend reversed in both sexes and resembled the results of the actual statistics. In both sexes, total CHD and stroke mortality always correlated negatively with oranges and mandarins.

## Discussion

### Raised cholesterol correlates negatively with CVD risk

The results of our study show that animal fat (and especially its combination with animal protein) is a very strong predictor of raised cholesterol levels. This is in accordance with the meta-analyses of clinical trials, which show that saturated animal fat is the major trigger of raised cholesterol (6, 16). Interestingly, the relationship between raised cholesterol and CVD indicators in the present study is always negative. As shown in Figs. 3 and 4 and Supplementary Figs. 1 and 2, this finding is visually less persuasive in the case of CVD mortality, where factors such as the quality of healthcare come to the foreground, but it is quite unambiguous in the case of women's raised blood pressure.

The negative relationship between raised cholesterol and CVD may seem counterintuitive, but it is not at variance with the available evidence. The largest of the recent worldwide meta-analyses dealing with cholesterol and CVD risk (17) observed a positive relationship between raised cholesterol and CVD mortality at younger ages, but this association gradually started to reverse in seniors, where the number of deaths is the highest. In fact, the relationship between raised cholesterol and stroke mortality in seniors was slightly negative. Both this study and other studies dealing with blood profiles of patients hospitalised with CVD events (18–22) demonstrate that low HDL (high-density lipoprotein associated) cholesterol (around ~1.0 mmol/L), or high total cholesterol: HDL-cholesterol ratio are the best indicators of CVD risk. Total cholesterol is usually normal or slightly elevated (4.5–5.5 mmol/L), and hence it cannot serve as a predictor of CVD events. Some other authors also point to high plasma triglycerides (which correlate with low HDL-cholesterol levels) (23), or to the ratio between triglycerides and HDL-cholesterol (24) as another useful risk indicators.

In this context it is important to note that saturated fat is not only the key trigger of high total cholesterol, but even high HDL-cholesterol and LDL (low-density lipoprotein associated)-cholesterol (16). Saturated fat also decreases triglyceride levels, but the total cholesterol: HDL-cholesterol ratio remains stable. The main sources of saturated fatty acids are red meat and milk products (whole fat milk, cheese, butter) (see Supplementary Table 1). Therefore, in Europe, where the consumption of animal products is the highest in the world, we can assume a strong connection between total cholesterol and HDL-cholesterol. Understandably, this relationship may not be so strong outside Europe and it may also vary depending on the individual diet. This could explain regional and individual differences in the relationship between total cholesterol and CVD risk.

Although the concurrent increase of LDL-cholesterol levels is often taken out of context and used as an argument against the intake of saturated fats in dietary recommendations (25), saturated fat is primarily tied to the less dense, large LDL particles (26), whereas cardiovascular risk is connected with the denser, small LDL particles (27), which accompany carbohydrate-based diets. There is also no evidence that the reduction of saturated fat intake (on its own) would decrease CVD risk (28). On the other hand, it is true, that so far, there is no clear evidence that saturated fat would be beneficial for the prevention of CVD. The only possible exception among the sources of saturated fat is dairy (29–31).

### Major correlates of high CVD risk

#### Carbohydrates

The results of our study show that high-glycaemic carbohydrates or a high overall proportion of carbohydrates in the diet are the key ecological correlates of CVD risk. These findings strikingly contradict the traditional ‘saturated fat hypothesis’, but in reality, they are compatible with the evidence accumulated from observational studies that points to both high glycaemic index and high glycaemic load (the amount of consumed carbohydrates × their glycaemic index) as important triggers of CVDs (1, 32–34). The highest glycaemic indices (GI) out of all basic food sources can be found in potatoes and cereal products (Supplementary Table 2), which also have one of the highest food insulin indices (FII) that betray their ability to increase insulin levels.

The role of the high glycaemic index/load can be explained by the hypothesis linking CVD risk to inflammation resulting from the excessive spikes of blood glucose (‘post-prandial hyperglycaemia’) (35). Furthermore, multiple clinical trials have demonstrated that when compared with low-carbohydrate diets, a low-fat diet increases plasma triglyceride levels and decreases total cholesterol and HDL-cholesterol, which generally indicates a higher CVD risk (36, 37). Simultaneously, LDL-cholesterol decreases as well and the number of dense, small LDL particles increases at the expense of less dense, large LDL particles, which also indicates increased CVD risk (27). These findings are mirrored even in the present study because cereals and carbohydrates in general emerge as the strongest correlates of low cholesterol levels.

In light of these findings, the negative correlation of refined sugar with CVD risk may seem surprising, but the mean daily consumption of refined sugar in Europe is quite low (~84 g/day), when compared with potato and cereal carbohydrates (~235 g/day), and makes up only ~20% of CA energy. Refined sugar is also positively tied to many animal products such as animal fat (*r*=0.57; *p*<0.001) and total fat and animal protein (*r*=0.52; *p*<0.001), and negatively to % PC CARB energy (*r*=−0.58; *p*<0.001) and % CA energy (*r*=−0.47; *p*=0.001). Therefore, a high consumption of refined sugar is accompanied by a high consumption of animal products and lower intakes of other carbohydrates. Furthermore, the glycaemic index of refined sugar (sucrose) is rather moderate (~65) (38).

#### Distilled beverages

Although alcohol (fermented carbohydrate) has almost zero values of GI and FII, its highly concentrated sources (distilled beverages) correlate moderately positively with raised blood pressure and CVD mortality in the present study, especially in men, in the absence of any relationship with blood cholesterol and blood glucose. Although distilled beverages played no important role in the regression models, they occupy a stable position in both factor analyses (Figs. 15 and 17) and are very loosely associated with other variables. They correlate significantly positively only with potatoes (*r*=0.52, *p*<0.001) and rye (*r*=0.49, *p*<0.001), which are the usual substrates for their production.

In contrast, the total consumption of alcoholic beverages, as well as the consumption of beer and wine, generally has a moderately negative relationship with CVD risk, although it may not necessarily be causal. In both factor analyses, alcoholic beverages and beer are never highlighted in the opposition against CVDs. In fact, the proportion of CA energy (carbohydrates and alcohol) correlates more strongly with CVD risk than the proportion of energy from carbohydrates alone, alcoholic beverages excluded (data not showed). This observation would suggest that the role of alcoholic beverages and beer in the Pearson linear correlations may be influenced by other factors. Indeed, alcoholic beverages correlate most negatively with % PC CARB energy (*r*=−0.68, *p*<0.001) and most positively with meat, particularly pork (both *r*=0.76, *p*<0.001).

In observational studies, alcohol generally appears as protective (1), but in reality, its relationship with CVD risk is J-shaped (39). Although it may be beneficial at low/moderate daily intake, this does not necessarily apply for excessive consumption above 5–10 g alcohol/day. Recently, Fillmore et al. (40) discussed an interesting hypothesis that this seemingly protective effect is due to methodological errors because the category ‘abstainers’ often includes former drinkers, who stopped drinking because of poor health. Still, our study offers some support to the protective role of wine, which is explained by the content of specific flavonoids in red wine (see below). Therefore, it is possible that the relationship of alcohol to CVD risk depends on the amount consumed, or on the content of specific plant extracts. Beer (GI 66, but a very low FII 20) contains both alcohol (3.9%) and carbohydrates (3.6%). Wine (GI 0, FII 3) has a much higher proportion of alcohol (10.4%) than carbohydrates (2.7%) (41). Distilled beverages (such as gin, rum, vodka, whiskey) contain zero carbohydrates, but ~40% alcohol. As a result, distilled beverages constitute only 6.1% of the total consumption of alcoholic beverages, but 26.4% of the total energy from alcoholic beverages. This fact can explain their negative health impact, relative to the small volume consumed. The dramatic increase of CVD mortality in the former USSR after 1989 (9) could also be linked with the heavy binge drinking of alcohol (vodka).

#### Sunflower oil

Sunflower oil belonged to the most consistent correlates of stroke mortality in the historical comparison. Its linear correlation with actual total CVD mortality is rather vague, but it markedly increases in the period 2000–2008. Because plant oil is generally associated with low CVD risk and sunflower oil is consumed mainly in the eastern half of Europe, where we find the highest intake of the supposed risk factors such as carbohydrates and distilled alcohol, its role could be disregarded as purely spurious. However, sunflower oil is only very loosely correlated with other variables in our dataset (*r*=0.41, *p*=0.007 with legumes; *r*=0.40, *p*=0.008 with onions; *r*=0.34, *p*=0.028 with smoking in men; *r*=0.31; *p*=0.045 with vegetables). Similar to distilled alcohol, sunflower oil was not highlighted by the penalised regression methods, but it creates very productive regression models (adj. R^2^) with some highly significant correlates of total CVD mortality, especially with % CA energy (63.9% of total variance in men, 75.9% in women) and total fat and total protein (62.3% in men, 76.3% in women). In contrast, onions do not improve these models virtually at all. For example, the combination of onions with % CA energy explains only 50.4 and 61.9% of total variability in men's and women's total CVD mortality, respectively.

At present, we do not have any reliable explanation for the peculiar role of sunflower oil in our analysis, but we think that there are several possibilities. First, sunflower oil has been the main component of solidified margarines, which were industrially produced from hydrogenated plant oils (trans-fatty acids). Trans-fatty acids (such as elaidic acid) are already recognised as an important risk of CVDs (42, 43). A weak point of this explanation is the fact that sunflower oil is consumed mainly in countries of Southeastern and Eastern Europe, where it is used in its unhydrogenated form in the local cuisine.

Second, some authors maintain that highly concentrated sources of linoleic acid [n-6 essential polyunsaturated fatty acid (PUFA)], containing only small amounts of alpha-linolenic acid (n-3 essential PUFA) (e.g. sunflower oil, corn oil), may have proinflammatory properties, but other data indicate the opposite (44). Our present study cannot illuminate this problem because corn oil correlates negatively with CVD risk (data not showed) and with regard to the low mean daily intake (2>g/day), its inclusion did not seem to be meaningful. Therefore, we must also work with a hypothesis that sunflower oil expresses some unknown confounder that is related to its culinary use. Perhaps even more likely, both sunflower oil and onions symbolise a diet in Southeastern and Eastern Europe, which is characterised by a low consumption of fruits, dairy, and animal products in general, and low health expenses. In any case, the significance (*p*<0.05) of sunflower oil as a correlate of total CVD mortality disappears when controlled for smoking (in men only) and health expenditure.

#### Onions

The role of onions as another potential risk factor is unclear and unexpected because *Allium* vegetables (onions and garlic) are often propagated as a prevention of CVDs (45). All we can say is that the role of onions is generally the weakest out of all positive correlates of CVD risk, which might indicate a spurious relationship. Similar to sunflower oil, onions are used as a food additive and they show the strongest positive correlation with vegetables (*r*=0.63; *p*<0.001) and % plant food energy in general (*r*=0.56; *p*<0.001). Onions do not correlate with men's raised blood pressure (*r*=0.20; *p*=0.21) and rather weakly with women's raised blood pressure (*r*=0.43; *p*=0.004). They also do not show any notable correlation with CVD indicators in the historical comparison and although they do show significant associations with the actual total CVD mortality, they do not contribute much to the regression models.

#### Smoking and BMI

Perhaps the most surprising finding of our study relates to CVDs and smoking because it differs by sex. Although smoking is the third strongest correlate of total CVD mortality in men (*r*=0.67; *p*<0.001), it has the opposite relationship in women (*r*=−0.46; *p*=0.002). The possibility that there would exist some sex-specific differences related to CVDs and smoking is very unlikely because the risky nature of smoking in women has been demonstrated quite persuasively (46). Therefore, we are apparently dealing with a mere statistical artefact. Furthermore, data from our study examining the determinants of cancer in 39 European countries (in preparation) demonstrate an expectable, positive link between smoking and the prevalence of lung cancer (*r*=0.41, *p*=0.01 in men; *r*=0.55, *p*<0.001 in women) and larynx cancer (*r*=0.49, *p*=0.002 in men; *r*=0.30, *p*=0.07 in women). These results indicate that the used statistics of smoking prevalence cannot be far from reality.

A closer examination of Figs. 11 and 12 reveals that an answer to this problem is not difficult. First, the mean prevalence of smoking in women (18.7%) is much lower than in men (38.0%). Second, the geographical pattern of smoking differs by sex (Supplementary Fig. 27). Although men smoke mainly in the former Soviet republics, the highest prevalence of smoking in women occurs in the Balkans and Northern and Central Europe. In general, smoking in women correlates with variables that appear as protective: total fat (*r*=0.58), total fat and animal protein (*r*=0.54), and high health expenditure (*r*=0.54; *p*<0.001).

Another problem related to smoking lies in its effect on BMI values. Smoking emerges as the only common denominator of low BMI values in both sexes (*r*=−0.47, *p*=0.002 in men; *r*=−0.53, *p*<0.001 in women) (Supplementary Figs. 29 and 30). This finding has been consistently documented even in observational studies because smoking increases energy expenditure and reduces appetite (47). As a result, the relationship between men's and women's BMI is curvilinear because smoking markedly decreases BMI values in men from the former USSR, when compared with women (Supplementary Fig. 28). This can explain, why the correlation between BMI and CVD indicators differs by sex (Figs. 13 and 14).

### Major correlates of low CVD risk

#### Fat and protein intake

Our finding that total fat and animal protein (or total fat and total protein) is the strongest correlate of low CVD risk is again in accordance with the hypothesis linking CVD risk to postprandial hyperglycaemia because a high consumption of fat and protein indicates a low dietary glycaemic load. Naturally, this observation also raises the question of whether our study can illuminate the unclear role of saturated fat. It is true that in clinical trials, the replacement of saturated fat with PUFAs decreases CVD risk (28, 48), but this evidence is not necessarily a proof of the harmful effect of saturated fat because PUFAs most effectively decrease total cholesterol: HDL ratio, LDL-cholesterol, and triglyceride levels (16). The richest natural sources of PUFAs are walnuts (47% weight) (41) and they also have a very good ratio between linoleic acid and alpha-linolenic acid (see Supplementary Table 1), not to mention further possible benefits on oxidative stress and inflammatory markers (49). This evidence supports the causal role of tree nuts in our study.

The factor and regression analyses indicate that the effect of dairy fat (and particularly its main dietary source – cheese) may be beneficial as well. This finding is in accordance with the growing evidence pointing to the preventive role of dairy (29–31). Although many observational studies still connect this effect with low-fat dairy (31), the positive role of dairy fat could be explained by the observations of clinical trials showing that lauric acid (12:0) and myristic acid (14:0), which are most abundant in dairy fat and particularly in coconut oil (Supplementary Table 1), most strongly increase HDL-cholesterol (16). Lauric acid also quite strongly decreases the total cholesterol: HDL-cholesterol ratio, whereas the effect of other saturated fatty acids is more or less neutral. However, some other factors unrelated to fat content may be comparably important. For example, cheese does not increase total and LDL-cholesterol, when compared with butter (50).

**Fig. 23:**
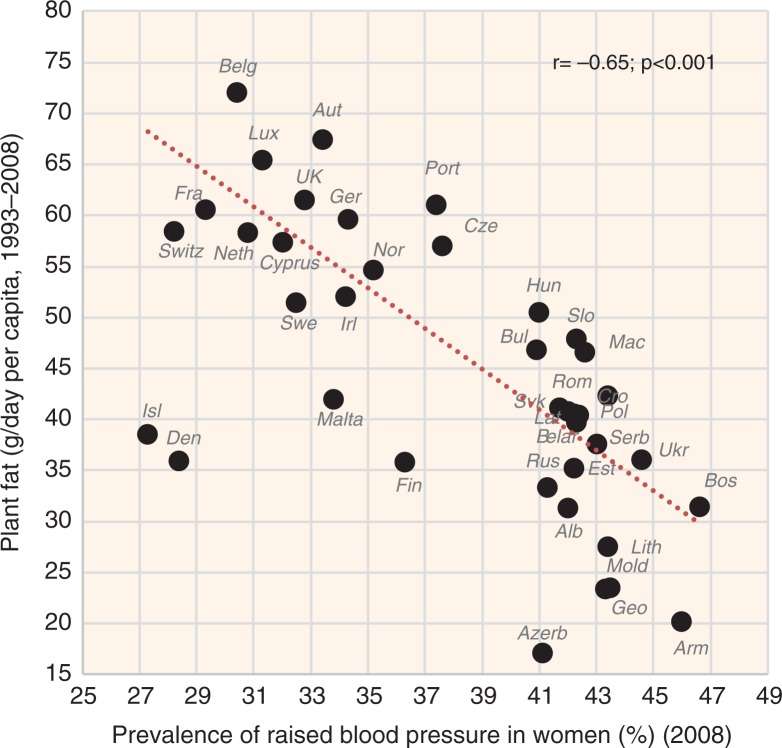
Correlation between the mean daily consumption of plant fat and the prevalence of raised blood pressure in women (*r*=−0.65; *p*=0.001).

**Fig. 24:**
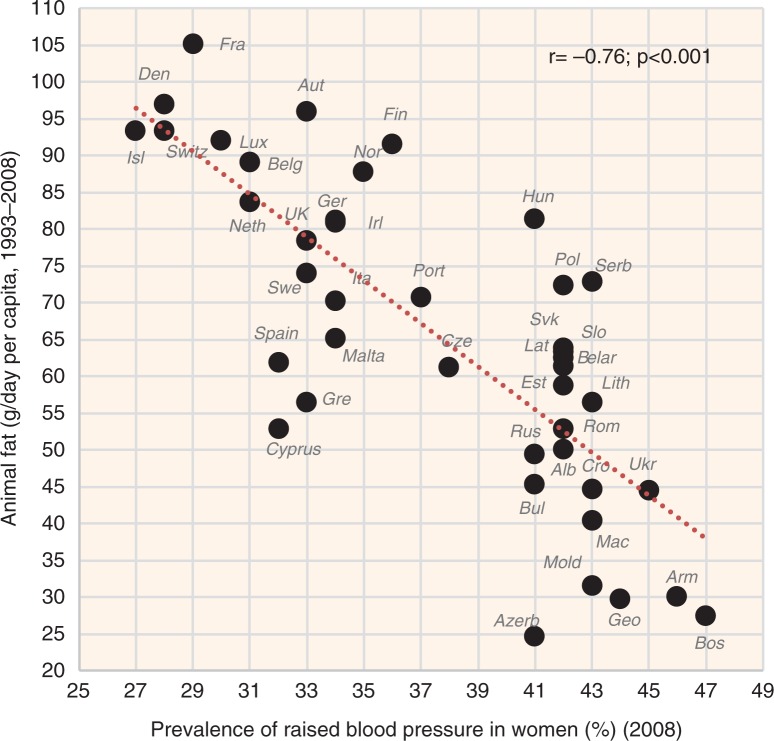
Correlation between the mean daily consumption of animal fat and the prevalence of raised blood pressure in women (*r*=−0.76; *p*=0.001).

**Fig. 25:**
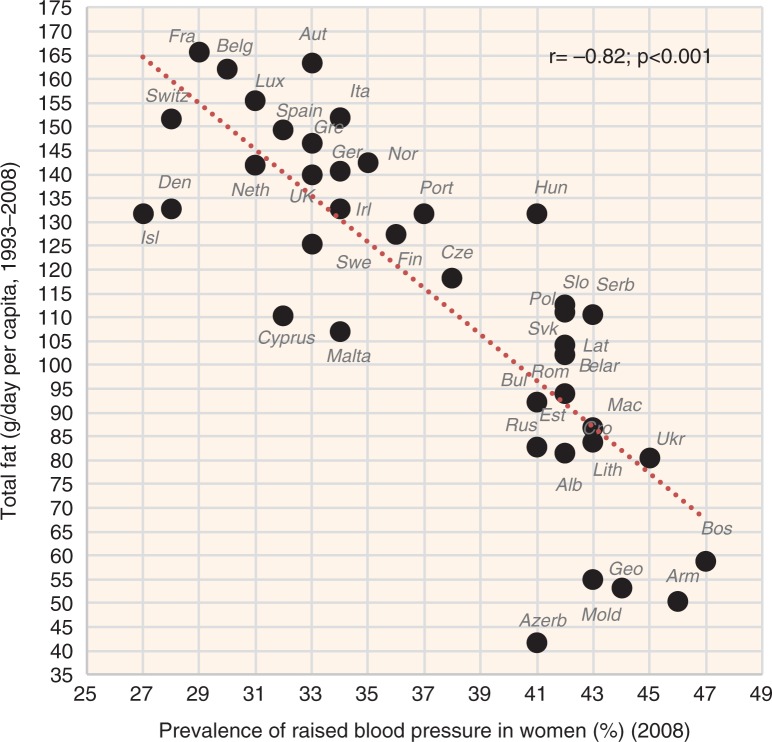
Correlation between the mean daily consumption of total fat and the prevalence of raised blood pressure in women (*r*=−0.82; *p*=0.001).

Fish & seafood (the source of long-chain PUFAs) is another animal item highlighted by the factor analysis, although its significance is rather secondary (Factor 3). This could also be attributed to small consumption rates constituting only 1.3% of total fat intake. In contrast, the role of meat fat (and meat in general) in the factor and regression analyses is rather marginal, which would indicate that it works rather neutrally and passively, via the decrease of the glycaemic load. This conclusion is complicated by the strong collinearity between meat and alcoholic beverages (*r*=0.76, *p*<0.001), which usually applies even at the individual level and it may effectively blur any health role of these two food items. In any case, both plant fat and animal fat correlate with low CVD risk and their combination further increases *r* values (see Figs. 23–25).

#### Fruits and wine

Both fruits (as a whole) and wine do not figure among the consistent correlates of CVD indicators in the historical comparison, but this can be ascribed to the fact that this comparison did not include the former Soviet republics, where their consumption is the lowest. Still, the only fruits that came to the foreground in all analyses were oranges and mandarins. Wine is remarkably independent of other food items and health expenditure during the period 1993–2008.

The protective effect of fruits and fruit products such as wine is mostly explained by the content of flavonoids (51). Flavonoids specific for citrus fruits are flavanones (52). Another promising group of flavonoids are anthocyanins (53) contained in berries, currants, and red wine (52). The position of ‘fruits total’ in the factor analysis indeed indicates that some non-tropical fruits may be both independent of healthcare and simultaneously related to low CVD risk.

#### Vegetables

Vegetables, which are frequently recommended as a precaution against CVDs because of their low glycaemic index, in the context of the ‘Mediterranean diet’, and emerge as a consistent protective factor even in observational studies (1), did not figure among the negative correlates of CVD risk. A closer examination of the graphic comparisons shows that vegetables have a basically curvilinear relationship to CVD risk, and they may work as a sort of prevention only when very high amounts (>300 g/day) are consumed (Supplementary Figs. 31 and 33), and especially when they substitute cereal and potato carbohydrates (Supplementary Figs. 32 and 34).

#### Health expenditure

Health expenditure (both for 2008 and 1995–2008) is the most negative correlate of total CVD mortality in both sexes and health expenditure for 1995–2008 is even the strongest negative correlate of raised blood pressure. It is also the main confounding factor in the whole study. The mean health expenditure for 1995–2008 correlates very strongly positively especially with total fat and animal protein (*r*=0.86, *p*<0.001), and even with some individual foodstuffs such as coffee (*r*=0.84, *p*<0.001), oranges and mandarins (*r*=0.80, *p*<0.001), and fruits (*r*=0.77, *p*<0.001). On the other hand, it correlates most negatively with % CA energy (*r*=−0.81, *p*<0.001).

**Fig. 26:**
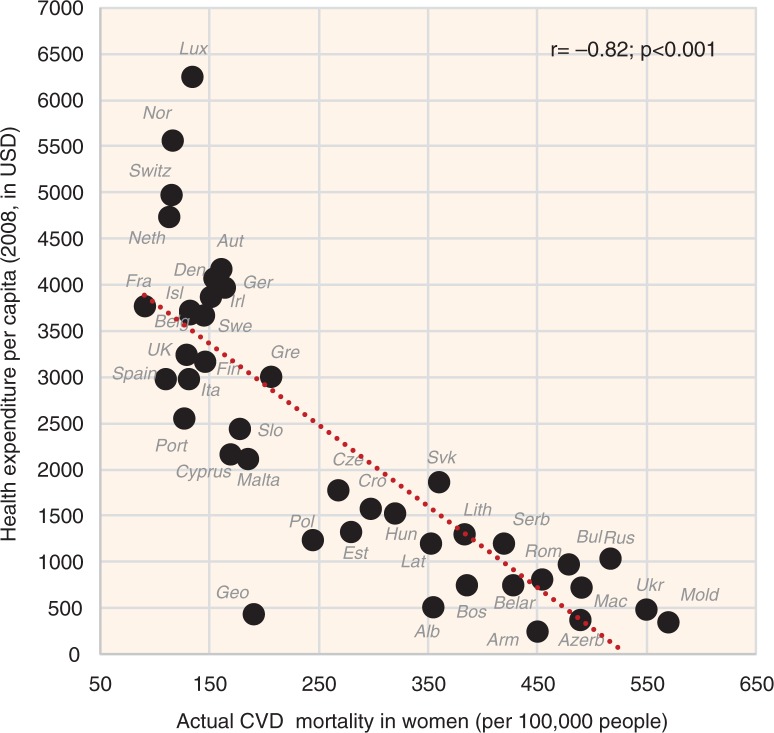
Correlation between health expenditure (2008) and the actual total CVD mortality in women (*r*=−0.82; *p*<0.001).

When comparing the relationship of health expenditure (2008) with CHD and total CVD mortality, we can see that it tends to be curvilinear; health expenses below 2000 USD per capita have no marked influence on the total CVD mortality in 21 countries (*r*=−0.34, *p*=0.14 in men; *r*=−0.50, *p*=0.021 in women), but mortality decreases dramatically in 21 countries above this value (Fig. 26). Higher health expenses do not bring any additional benefits. We can also observe that health expenditure markedly influences the relationship of certain variables with total CVD mortality, as evidenced by a similar curvilinear relationship found in fruits (Supplementary Fig. 5), oranges and mandarins (Supplementary Fig. 6), tree nuts (Supplementary Fig. 14), coffee, and even in raised blood pressure (Supplementary Figs. 35 and 36). Provided that some of these variables have a causal relationship to CVD risk, their ability to influence mortality must be substantially lower, when compared with health expenditure.

**Fig. 27:**
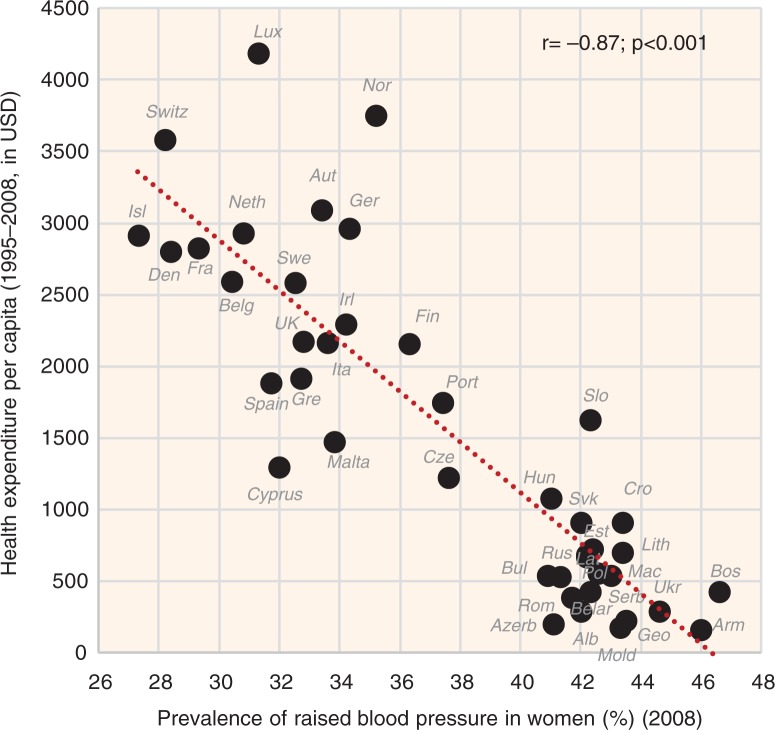
Correlation between health expenditure (1995–2008) and the prevalence of raised blood pressure in women (*r*=−0.87; *p*<0.001).

Remarkably, we do not observe such a curvilinear trend in the basic components of diet – fat, protein, or carbohydrate intake (Figs. 7 and 10), despite the fact that these items are very strongly correlated with health expenditure. We also do not observe it between health expenditure and raised blood pressure (Fig. 27), not to mention raised blood glucose. Because raised blood pressure includes both people with raised blood pressure and taking medications, it reflects total CVD prevalence in the population that results from the individual lifestyle. Hence it should be independent of healthcare. The fact that health expenditure (1995–2008) is the strongest negative correlate of raised blood pressure is thus unexpected. However, its relationship with raised blood pressure in the wealthier half of Europe is quite vague (compare Figs. 26 and 27), which is the exact opposite of what we would expect, provided that high health expenses would decrease the prevalence of this indicator. Therefore, it is more likely that the significant role of health expenditure results from the multiple collinearity with other protective variables.

### Discrepancy in the old statistics: The root of the ‘saturated fat hypothesis’?

The paradoxical results of our historical comparison (men's statistics from 1980 and 1990) have an interesting analogy in the ‘Seven Countries Study’, which stood behind the current ‘saturated fat paradigm’. The authors of this longitudinal ecological research, finished in the early 1980s, concluded that men's CHD mortality in seven countries correlated positively with high blood pressure, high cholesterol, and high saturated fat intake, but the relationship of high blood pressure and high cholesterol with men's stroke mortality (in 12 cohorts from six countries) was strongly negative (54). Because CHD mortality was the central CVD indicator in this study, we think that the authors did not pay sufficient attention to this discrepancy and they contented themselves with the fact that stroke mortality was positively associated with high blood pressure at the individual level. Ironically, in the following decades, the ecological relationship of CHD with risk factors completely reversed.

Using the historical European statistics of CHD mortality (men and women aged 35–74 years) (55), we found that in 21 countries, the same positive correlation existed between CHD mortality in 1970 and animal fat consumption between 1961 and 1970, *in both sexes* (*r*=0.71, *p*<0.001 in men; *r*=0.53, *p*=0.013 in women). In 1980, CHD mortality in 24 countries still correlated with animal fat intake (1965–1980) in men (*r*=0.60, *p*=0.002), but it already lost significance in women (*r*=0.29, *p*=0.16), which is in accordance with the data in Tables 2 and 3.

Although we are not familiar with the period methodology of data collection, we can get some clue about the root of this discrepancy. A closer look at the period statistics (Supplementary Dataset, Sheet S4) shows that the mean ratio between CHD/stroke mortality between 1980 and 2000 did not change much, even if we consider the same limited sample of 16 countries from 1980 (2.5–2.8:1 for men, 1.2–1.4:1 for women), but in some developed countries such as the UK, the Netherlands, Malta, Ireland, and Iceland, it was always eccentrically high (as much as 10:1 for men in Iceland and 2.4:1 for women in Malta). This can explain why the rates of CHD mortality in these countries were increased in the past, why the rates of stroke mortality were disproportionately low, and why raised blood pressure did not correlate consistently with CVD risk. In contrast, the combined CHD and stroke mortality, which better reflects total CVD mortality, tended to correlate positively with blood pressure, as expected (compare Supplementary Figs. 37–39). Despite the fact that the CHD/stroke mortality ratio in these countries has remained high, the rates of total CVD mortality have decreased very dramatically in recent decades (by as much as two-thirds). At the same time, total CVD mortality rates in some Eastern European countries (e.g. Romania, Belarus, Ukraine, Russia) have increased by as much as one-half between 1980 and 2000 (Supplementary Table 11). This was enough to reverse the correlation between CHD mortality and food consumption.

These findings offer two possible explanations. First, there are persistent problems with misdiagnosis in some European countries. Although the current rates of CHD mortality strongly correlate with total CVD mortality (*r*=0.85 in men, *r*=0.76 in women; *p*<0.001) (Supplementary Figs. 40 and 41), there are notable outliers such as Bulgaria, Macedonia, Azerbaijan, Serbia, and Bosnia and Herzegovina, where CHD mortality is much lower than this correlation predicts. For example, the actual total CVD mortality in men from Armenia (640.4 cases per 100,000 people) and Azerbaijan (616.8 cases) is nearly the same, but the actual CHD mortality in Armenia (407.4 cases) is almost three times higher than in Azerbaijan (149.3 cases). Such a huge difference between two neighbouring countries with a similar diet is very unlikely. Naturally, this also shows that CHD statistics may not be an optimal tool for ecological studies and total CVD mortality should always be preferred.

Second, the effect of increased longevity in highly developed countries, after the eradication of serious infectious diseases after World War II, led to a temporary epidemic of CVDs, which also coincided with rapidly improving living standards and the increasing consumption of animal food. Because of the chronic nature of CVDs, this dietary change may have brought health benefits only after several decades and as a result, the relationship of CVD indicators to animal products has reversed with a certain delay (Supplementary Figs. 42–47). In the eastern half of Europe, this phenomenon started to fully manifest only very recently (possibly in combination with heavy alcohol drinking), which led to the increase of CVD rates.

**Table 4:** Correlations between the mean food consumption (1961–2008) and the actual statistics of cardiovascular diseases in 24 countries

	Raised cholesterol (2008)		Raised blood pressure (2008)		Raised fasting blood glucose (2008)		Actual CHD mortality		Actual total CVD mortality		Life expectancy (2008)	
					
	Men	Women	Men	Women	Men	Women	Men	Women	Men	Women	Men	Women
Fruits	0.13	0.12	−0.60	−0.54	−0.14	−0.35	−0.51	−0.51	−0.50	−0.42	0.56	0.49
Bananas	0.68	0.59	−0.48	−0.69	−0.09	−0.46	−0.44	−0.47	−0.77	−0.79	0.79	0.72
Oranges and mandarins	0.34	0.29	−0.67	−0.69	−0.22	−0.41	−0.67	−0.69	−0.79	−0.74	0.80	0.74
Distilled beverages	−0.16	−0.15	0.55	0.46	−0.10	0.05	0.20	0.18	0.37	0.32	−0.47	−0.41
Wine	0.00	0.09	−0.21	−0.18	−0.29	−0.32	−0.37	−0.29	−0.25	−0.23	0.13	0.31
Coffee	0.49	0.46	−0.36	−0.56	−0.11	−0.43	−0.29	−0.31	−0.47	−0.53	0.53	0.47
Cereals	−0.70	−0.61	0.54	0.77	0.29	0.61	0.46	0.50	0.78	0.80	−0.72	−0.63
Sunflower oil	−0.62	−0.53	0.35	0.48	0.22	0.46	0.34	0.39	0.71	0.73	−0.65	−0.59
Potatoes	0.35	0.23	0.12	0.04	−0.41	−0.29	−0.17	−0.20	−0.25	−0.31	−0.01	0.10
Vegetables	−0.52	−0.49	−0.16	0.18	0.08	0.32	−0.12	−0.08	0.11	0.20	−0.18	−0.08
Onions	−0.45	−0.50	0.04	0.31	0.10	0.45	0.14	0.18	0.28	0.33	−0.40	−0.33
Plant fat	−0.02	−0.08	−0.60	−0.40	−0.17	−0.17	−0.60	−0.57	−0.45	−0.36	0.45	0.44
Dairy total	0.41	0.37	−0.20	−0.44	−0.36	−0.58	−0.22	−0.27	−0.41	−0.47	0.54	0.50
Meat total	0.60	0.56	−0.34	−0.48	−0.33	−0.51	−0.11	−0.13	−0.38	−0.43	0.27	0.34
Animal protein	0.75	0.68	−0.44	−0.69	−0.26	−0.55	−0.34	−0.39	−0.59	−0.65	0.63	0.61
Animal fat	0.70	0.71	−0.14	−0.44	−0.28	−0.57	−0.05	−0.10	−0.37	−0.47	0.32	0.36
Animal fat and animal protein	0.77	0.75	−0.27	−0.56	−0.29	−0.60	−0.17	−0.22	−0.48	−0.58	0.46	0.49
Total protein	0.45	0.43	−0.29	−0.38	−0.12	−0.25	−0.18	−0.22	−0.28	−0.33	0.34	0.40
Total fat	0.70	0.67	−0.54	−0.71	−0.40	−0.69	−0.45	−0.48	−0.67	−0.72	0.63	0.66
Total fat and animal protein	0.77	0.72	−0.54	−0.75	−0.37	−0.68	−0.44	−0.48	−0.68	−0.74	0.67	0.69
Total fat and total protein	0.71	0.68	−0.52	−0.69	−0.36	−0.63	−0.42	−0.46	−0.63	−0.68	0.61	0.66
CA energy	−0.38	−0.32	0.50	0.63	−0.01	0.27	0.33	0.35	0.57	0.56	−0.61	−0.49
% CA energy	−0.73	−0.67	0.63	0.82	0.28	0.63	0.48	0.52	0.76	0.80	−0.75	−0.72
Raised blood pressure (men)	−0.43				0.15		0.61		0.65		−0.73	
Raised blood press. (women)		−0.60				0.56		0.64		0.77		−0.75
Health expenditure (2008)	0.70	0.62	−0.54	−0.76	−0.34	−0.66	−0.59	−0.60	−0.78	−0.79	0.80	0.73
Health exp. (1995–2008)	0.73	0.65	−0.56	−0.78	−0.31	−0.66	−0.58	−0.58	−0.78	−0.79	0.80	0.76

Note: The mean food consumption in Belgium was based on the combination of data from Belgium and Luxembourg (1961–1999) and Belgium (2000–2008).
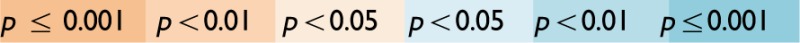

The obvious fallacy of the ‘saturated fat hypothesis’ can be demonstrated by the example of France – a country with the highest intake of animal fat in the world and the second lowest CVD mortality (after Japan) (56). In fact, if we use a limited sample of 24 countries (without the former republics of USSR, Czechoslovakia and Yugoslavia, and Luxembourg), a summary mean of food consumption from the last half-century (1961–2008) produces very similar results like the mean for the period 1993–2008 (Table 4), reaching *r*=0.82 between % CA energy and raised blood pressure in women. Remarkably, onions do not play any substantial role in this comparison and sunflower oil is again strongly associated only with CVD mortality.

### Strengths and limitations of this study

The results of the present study confirmed our previous experience and showed that an ecological approach based on statistics from renowned international sources can produce very useful findings. We can reasonably assume that correlations as high as *r*=0.92 would find very few parallels in the epidemiological literature. Although the FAOSTAT database cannot provide maximally precise data of consumed food because a certain amount is wasted, the proportion of this wasted food must be very similar in all countries and has no influence on the practical usability of these statistics. Furthermore, we have already verified the usefulness of this database in our previous studies dealing with the determinants of body height. Therefore, we think that we can have a legitimate confidence in the accuracy of these longitudinal food statistics, and this can be regarded as the main advantage of this study.

The extremely high correlations also indirectly support the high accuracy of the statistics of raised blood cholesterol and raised blood glucose. The statistics of total CHD mortality cannot be regarded as perfectly reliable, but total CVD mortality should sufficiently compensate for this shortcoming because the pathophysiology of CVDs is similar. However, mortality statistics are influenced by the quality of healthcare and the conclusions of our study thus primarily depend on the statistics of CVD prevalence, represented by raised blood pressure. We cannot be responsible for the accuracy of these statistics, but if they were influenced by any bias, we would expect under-reporting of raised blood pressure in less developed countries with low health expenditure. Apparently, this is not the case – at least in women – and the statistics of total CVD mortality and CVD prevalence (including raised blood glucose) correlate strongly positively with each other.

The reasonably high accuracy of the input data, combined with some extremely high correlations, together substantially increase the likelihood of true causal relationships, especially when the results concern principal components of food with high consumption rates, and when they can be supported by other sources. In items of smaller importance (e.g. distilled beverages, sunflower oil, onions), the results are less persuasive and their interpretation is not always easy and straightforward. Similar to observational studies, our ecological study reflects ‘real-world data’ and cannot always separate mutual interactions among the examined variables. Therefore, the reliance on bivariate correlations could lead to misleading conclusions. However, some of these findings can be used as a starting point of medical hypotheses, whose validity can be investigated in controlled clinical trials.

Understandably, our list of variables potentially contributing to the development of CVDs cannot be regarded as exhaustive. For example, the information on physical activity was self-reported and incomplete, and the FAOSTAT database lists no data on salt intake. The available statistics of sodium intake (for 2010), based on 24-h urinary sodium excretion (57), correlate moderately with raised blood pressure (*r*=0.39, *p*=0.01 in men; *r*=0.48, *p*=0.001 in women), but only weakly with total CVD mortality (*r*=0.32, *p*=0.039 in men; *r*=0.33, *p*=0.031 in women).

## Conclusion

Irrespective of the possible limitations of the ecological study design, the undisputable finding of our paper is the fact that the highest CVD prevalence can be found in countries with the highest carbohydrate consumption, whereas the lowest CVD prevalence is typical of countries with the highest intake of fat and protein. The polarity between these geographical patterns is striking. At the same time, it is important to emphasise that we are dealing with the most essential components of the everyday diet.

Health expenditure – the main confounder in this study – is clearly related to CVD mortality, but its influence is not apparent in the case of raised blood pressure or blood glucose, which depend on the individual lifestyle. It is also difficult to imagine that health expenditure would be able to completely reverse the connection between nutrition and all the selected CVD indicators. Therefore, the strong ecological relationship between CVD prevalence and carbohydrate consumption is a serious challenge to the current concepts of the aetiology of CVD.

The positive effect of low-carbohydrate diets on CVD risk factors (obesity, blood lipids, blood glucose, insulin, blood pressure) is already apparent in short-term clinical trials lasting 3–36 months (58) and low-carbohydrate diets also appear superior to low-fat diets in this regard (36, 37). However, these findings are still not reflected by official dietary recommendations that continue to perpetuate the unproven connection between saturated fat and CVDs (25). Understandably, because of the chronic nature of CVDs, the evidence for the connection between carbohydrates and CVD events/mortality comes mainly from longitudinal observational studies and there is a lack of long-term clinical trials that would provide definitive proof of such a connection. Therefore, our data based on long-term statistics of food consumption can be important for the direction of future research.

**Fig. 28:**
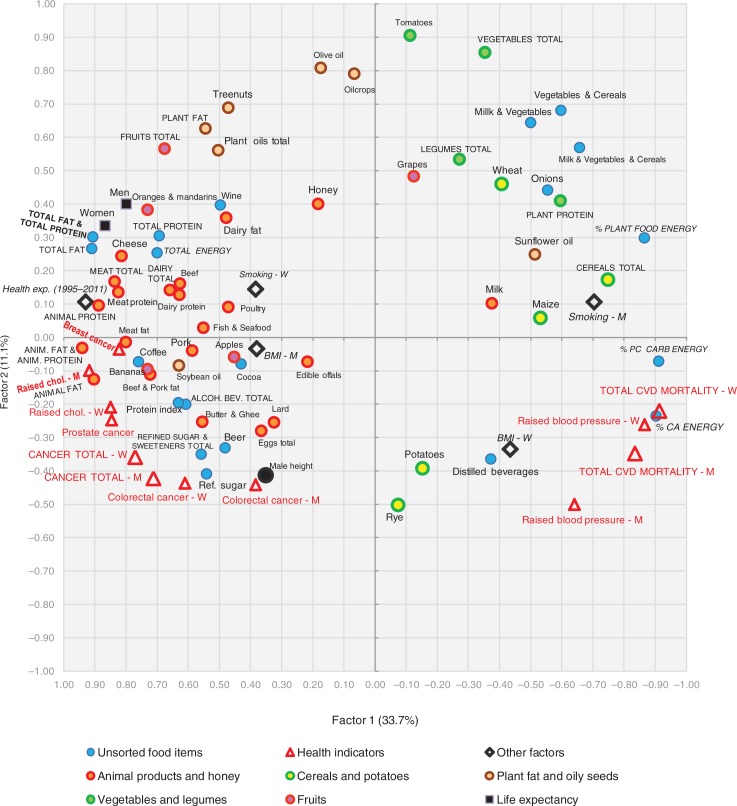
A plot of two principal components (Factor 1 and Factor 2) explaining 44.8% variability in the correlation between food consumption (1993–2011), BMI, smoking, health expenditure, cancer incidence (2012), and actual CVD indicators in 39 European countries. For better clarity, some less important or too repetitive variables (e.g. fish and seafood fat, and potato and cereal energy) were omitted. % CA energy=energy from carbohydrates and alcohol (as % of total energy), % PC CARB energy=energy from potato and cereal carbohydrates (as % of total energy), BMI=body mass index, CVD=cardiovascular diseases, Health exp. (1995–2011)=mean health expenditure per capita for 1995–2011, Raised chol.=raised cholesterol, M=men, W=women. The ‘Protein Index’ is the strongest correlate of male height and expresses a high dietary protein quality (the ratio between dairy and wheat proteins).

In fact, our ecological comparison of cancer incidence in 39 European countries (for 2012; (59)) can bring another important argument. Current rates of cancer incidence in Europe are namely the exact geographical opposite of CVDs (see Fig. 28). In sharp contrast to CVDs, cancer correlates with the consumption of animal food (particularly animal fat), alcohol, a high dietary protein quality, high cholesterol levels, high health expenditure, and above average height. These contrasting patterns mirror physiological mechanisms underlying physical growth and the development of cancer and CVDs (60). The best example of this health paradox is again that of French men, who have the lowest rates of CVD mortality in Europe, but the highest rates of cancer incidence. In other words, cancer and CVDs appear to express two extremes of a fundamental metabolic disbalance that is related to factors such as cholesterol and IGF-1 (insulin-like growth factor).

Besides total fat and protein consumption, the most likely preventive factors emerging in our study include fruits (particularly citrus fruits), wine, high-fat dairy products (especially cheese), sources of plant fat (tree nuts, olives), and potentially even vegetables and other low-glycaemic plant sources, provided that they substitute high-glycaemic foods. Many of these foodstuffs are the traditional components of the ‘Mediterranean diet’, which again strengthens the meaningfulness of our results. The factor analysis (Factor 3) also highlighted coffee, soybean oil and fish & seafood, but except for the fish & seafood, the rationale of this finding is less clear, because coffee is strongly associated with fruit consumption and soybean oil is used for various culinary purposes. Still, some support for the preventive role of coffee does exist (61) and hence, this observation should not be disregarded.

Similar to the “Mediterranean diet”, the Dietary Approaches to Stop Hypertension (DASH) diet, which is based mainly on fruits, vegetables, and low-fat dairy, also proved to be quite effective (62). However, our data indicate that the consumption of low-fat dairy may not be an optimal strategy. Considering the unreliability of observational studies highlighting low-fat dairy and the existence of strong bias regarding the intake of saturated fat, the health effect of various dairy products should be carefully tested in controlled clinical studies. In any case, our findings indicate that citrus fruits, high-fat dairy (such as cheese) and tree nuts (walnuts) constitute the most promising components of a prevention diet.

Among other potential triggers of CVDs, we should especially stress distilled beverages, which consistently correlate with CVD risk, in the absence of any relationship with health expenditure. The possible role of sunflower oil and onions is much less clear. Although sunflower oil consistently correlates with stroke mortality in the historical comparison and creates very productive regression models with some correlates of the actual CVD mortality, it is possible that both these food items mirror an environment that is deficient in some important factors correlating negatively with CVD risk.

A very important case is that of cereals because whole grain cereals are often propagated as CVD prevention. It is true that whole grain cereals are usually characterised by lower GI and FII values than refined cereals, and their benefits have been documented in numerous observational studies (63), but their consumption is also tied with a healthy lifestyle. All the available clinical trials have been of short duration and have produced inconsistent results indicating that the possible benefits are related to the substitution of refined cereals for whole grain cereals, and not because of whole grain cereals *per se* (64, 65). Our study cannot differentiate between refined and unrefined cereals, but both are highly concentrated sources of carbohydrates (~70–75% weight, ~80–90% energy) and cereals also make up ~50% of CA energy intake in general. To use an analogy with smoking, a switch from unfiltered to filtered cigarettes can reduce health risks, but this fact does not mean that filtered cigarettes should be propagated as part of a healthy lifestyle. In fact, even some unrefined cereals [such as the ‘whole-meal bread’ tested by Bao et al. (32)] have high glycaemic and insulin indices, and the values are often unpredictable. Therefore, in the light of the growing evidence pointing to the negative role of carbohydrates, and considering the lack of any association between saturated fat and CVDs, we are convinced that the current recommendations regarding diet and CVDs should be seriously reconsidered.

## Supplementary Material

Food consumption and the actual statistics of cardiovascular diseases: an epidemiological comparison of 42 European countries

Food consumption and the actual statistics of cardiovascular diseases: an epidemiological comparison of 42 European countries
