# Supplementary material for: Food consumption and the actual statistics of cardiovascular diseases: an epidemiological comparison of 42 European countries
Source: Food Nutr Res. 2016 Sep 27;60:10.3402/fnr.v60.31694. doi: 10.3402/fnr.v60.31694 (PMC5040825; doi:10.3402/fnr.v60.31694)
Supplement: Food consumption and the actual statistics of cardiovascular diseases: an epidemiological comparison of 42 European countries [file FNR-60-31694-s002.pdf]

Supplementary tables and figures

Supplementary Table 1. Composition of fat in various food items. (Fatty acids in %)

	Saturated fatty acids					MUFA (monounsatur.)			PUFA (polyunsaturated)					
	Total	12:0 Lauric	14:0 Myristic	16:0 Palmitic	18:0 Stearic	Total	18:1 (Oleic)*	18:1t Elaidic or Vaccenic	Total	18:2 (Linoleic)*	18:3 (ALA)*	20:4 ARA**	20:5 EPA	22:6 DHA
**DAIRY & EGGS**														
**Butter, w/o salt**	**62,25**	3,19	9,17	26,75	12,33	**28,89**	20,93	3,68	**3,71**	2,67	0,39	0,00	0,00	0,00
**Cheese, Mozzarella (22,4% fat)**	**58,84**	3,09	9,79	23,85	10,93	**29,41**	25,27[1]		**3,42**	1,76[2]	1,66[3]	0,00	0,00	0,00
**Cheese, cheddar (20,4% fat)**	**61,75**	3,14	10,05	28,22	10,58	**25,99**	20,46	2,52	**4,38**	2,43	0,30	0,17	0,03	0,00
**Goat milk (4,1% fat)**	**64,42**	3,00	7,85	22,01	10,65	**26,79**	23,60[1]		**3,60**	2,63[2]	0,97[3]	0,00	0,00	0,00
**Low -fat milk (1.0% fat)**	**65,26**	2,99	9,38	29,59	12,99	**28,56**	21,96	3,81	**3,61**	2,78	0,41	0,00	0,00	0,00
**Whole milk (3.3% fat)**	**57,03**	2,35	9,08	25,35	11,16	**24,83**	24,83[1]		**5,96**	3,67[2]	2,29[3]	0,00	0,00	0,00
**Eggs, hard-boiled**	**30,79**	0,03	0,33	22,14	7,80	**38,43**	35,11[1]		**13,33**	11,20[2]	0,33[3]	1,40	0,05	0,36
**MEAT**														
**Beef brisket, cooked**	**37,53**	0,07	3,26	23,11	10,94	**46,81**	42,03[1]		**2,61**	2,17[2]	0,22[3]	0,22		
**Chicken breast, cooked**	**31,29**	0,16	0,60	22,21	7,44	**38,23**	32,52	0,28	**24,10**	18,27	0,76	2,68	0,13	0,22
**Lard**	**39,20**	0,20	1,30	23,80	13,50	**45,10**	41,20[1]		**11,20**	10,20[2]	1,00[3]	0,00	0,00	0,00
**Mutton tallow**	**47,30**	0,00	3,80	21,50	19,50	**40,60**	37,60[1]		**7,80[2]**	5,50[3]	2,30[4]	0,00	0,00	0,00
**Pork bacon, baked**	**32,79**	0,00	1,27	20,86	10,19	**44,06**	40,30[1]		**11,23**	9,45	0,49	0,35	0,00	0,00
**FISH**														
**Carp, raw**	**19,34**	0,00	2,04	11,73	3,30	**41,57**	20,54[1]		**25,55**	9,23[2]	4,82[3]	2,71	4,25	2,04
**Herring (Atlantic), raw**	**22,57**	0,13	6,13	14,97	1,21	**41,33**	16,77[1]		**23,60**	1,44[2]	1,14[3]	0,66	7,84	9,54
**Salmon (Atlantic), farmed, raw**	**22,73**	0,00	4,14	13,99	3,69	**28,09**	20,23[1]		**28,96**	6,71[2]	1,10	0,69	6,42	8,23
**Sardine (Atlantic) & plant oil**	**13,35**	0,00	1,68	8,67	3,00	**33,79**	18,73[1]		**44,96**	30,94[2]	4,35[3]	0,00	4,13	4,45
**Sardine (Pacific)&tomato sauce**	**25,34**	0,00	6,72	16,63	2,33	**46,10**	17,70[1]		**20,20**	1,18[2]	2,25[3]	2,84	5,09	8,27
**Mackerel (Atlantic), cooked**	**23,45**	0,12	4,85	15,30	3,05	**39,28**	16,44		**24,12**	1,58[2]	1,14[3]	1,32	6,46	10,09
**Tuna, yellow fin, raw**	**35,10**	0,20	1,84	21,63	9,18	**23,67**	16,33	0,61	**30,00**	1,22	0,41	3,06	2,45	17,96
**PLANT SEEDS &OILS**														
**Almonds**	**7,62**	0,00	0,01	6,18	1,41	**63,20**	62,68	0,00	**24,69**	24,68	0,01	0,00	0,00	0,00
**Cocoa butter oil**	**59,70**	0,00	0,10	25,40	33,20	**32,90**	32,60[1]		**3,00**	2,80[2]	0,10[3]	0,00	0,00	0,00
**Coconut oil**	**86,50**	44,60	16,80	8,20	2,80	**5,80**	5,80[1]		**1,80**	1,80[2]	0,00	0,00	0,00	0,00
**Corn oil, all-purpose**	**12,95**	0,00	0,02	10,58	1,85	**27,58**	27,33	0,00	**54,68**	53,23	1,16	0,00	0,00	0,00
**Hazelnuts**	**7,35**	0,00	0,00	5,10	2,08	**75,14**	74,74[1]	0,00	**13,04**	12,89[1]	0,14[3]	0,00	0,00	0,00
**Margarine (corn & soybean oil)**	**18,82**	0,00	0,06	10,45	7,65	**48,17**	30,34	17,58	**30,11**	26,67	2,42	0,00	0,00	0,00
**Mustard oil**	**11,58**		1,39	3,75	1,12	**59,19**	11,61[1]		**21,23**	15,33[2]	5,90[3]			
**Olive oil (salad or cooking)**	**13,81**	0,00	0,00	11,29	1,95	**72,96**	71,27[1]		**10,52**	9,76[2]	0,76[3]	0,00	0,00	0,00
**Palm oil**	**49,30**	0,10	1,00	43,50	4,30	**37,00**	36,60[1]		**9,30**	9,10	0,20[3]	0,00	0,00	0,00
**Poppy seeds**	**10,87**	0,00	0,19	8,62	1,88	**14,39**	14,11	0,00	**68,74**	68,08[2]	0,66	0,00	0,00	0,00
**Rape (canola) oil**	**7,37**	0,00	0,00	4,30	2,09	**63,28**	61,71	0,03	**28,14**	18,64	9,14	0,00	0,00	0,00
**Soybean oil**	**15,65**	0,00	0,00	10,46	4,44	**22,78**	22,55	0,00	**57,74**	50,42	6,79	0,00	0,00	0,00
**Sunflower oil, high-oleic**	**9,86**	0,00	0,06	3,68	4,32	**83,69**	82,63[1]		**3,80**	3,61[2]	0,19[3]	0,00	0,00	0,00
**Sunflower oil, linoleic**	**10,30**	0,00	0,00	5,90	4,50	**19,50**	19,50[1]		**65,70**	65,70[2]	0,00	0,00	0,00	0,00
**Walnuts, English**	**9,39**	0,00	0,00	6,75	2,54	**13,70**	13,49[1]		**72,34**	58,42[2]	13,92[3]	0,00	0,00	0,00

Notes: ALA = α-linolenic acid, EPA = eicosapentaenoic acid, DHA = docosahexaenoic acid.
*Includes this fatty acid, unless stated otherwise.
**Undifferentiated 20:4. Equals to arachidonic acid (ARA) (20:4, n-6) in animal fat.
[1]Undifferentiated 18:1. May include vaccenic acid (18:1t, n-7) found in animal fat, or elaidic acid (18:1t, n-9) found in hydrogenated plant oils (margarines).
[2]Undifferentiated 18:2. May include rumenic acid (18:2t, n-7) found in animal fat.
[3]Undifferentiated 18:3. May include γ-linolenic acid (18:3, n-6) and some conjugated 18:3 fatty acids.

Source: After the USDA.gov database [41].

Supplementary Table 2. Glycaemic index and insulin index in selected foods. A comparison of 1000 kJ-portions, relative to glucose.

GLYCAEMIC INDEX		INSULIN INDEX	
Glucose	100	Glucose	100
Potatoes (boiled)	78	Potatoes (boiled)	88
„Whole-meal bread“ (Australia)	74	Baked beans	88
White rice	72	White bread	73
Brown rice	72	„Whole-meal bread“ (Australia)	70
White bread	70	Ice tea (Narkena Ltd.)	69
Beer (4.9% alcohol)	66	Black grapes	60
Pizza	60	Skim-fat milk (0.1%)	60
Ice tea (Narkena Ltd.)	59	Bananas	59
Coca-Cola	53	White rice	58
Bananas	52	Low-fat cottage cheese (2.3% fat)	52
Black grapes	50	Pizza	47
Spiral white pasta	46	Brown rice	45
„Grain bread“ (Australia)	36-50	Coca-Cola	44
Baked beans	44	Oranges	44
Oranges	42	Apples (Red delicious)	43
Lentils served with tomato sauce	37	Lentils served with tomato sauce	42
Apples (Red delicious)	36	„Grain bread“ (Australia)	41-52
Whole-fat milk (3.8%)	31	Beef steak	37
Low-fat milk (1.0%)	29	Low-fat milk (1.0%)	34
Skim-fat milk (0.1%)	29	Cheddar cheese (35.6% fat)	33
Hot-dog (sausage)	28	Spiral white pasta	29
Low-fat cottage cheese (2.3% fat)	0	Whole-fat milk (3.8%)	24
Beef steak	0	Eggs (poached)	23
Cheddar cheese (35.6% fat)	0	Beer (4.9% alcohol)	20
Eggs (poached)	0	Chicken (fried in olive oil)	19
Chicken (fried in olive oil)	0	Cream cheese (35.3% fat)	18
Cream cheese (35.3% fat)	0	Hot-dog (sausage)	16
Tuna (canned in oil)	0	Tuna (canned in oil)	16
Walnuts	0	Walnuts	5
Avocado	0	Avocado	4
Olive oil	0	Olive oil	3
White wine (11% alcohol)	0	White wine (11% alcohol)	3
Butter	0	Butter	2
Gin (40% alcohol)	0	Gin (40% alcohol)	1

Source: After Bao et al. [32].

Supplementary Table 3a. Raised blood pressure (Men): Parsimonious models of the ridge regression, LASSO regression and elastic net regression, computed by the bootstrapping method and sorted according to the absolute *beta* coefficients of the ridge regression and elastic net regression. In the ridge regression, all food items that reach a non-zero value in the elastic net regression are listed.

VARIABLES	Ridge		LASSO		VARIABLES	Elastic net	
	Beta	absolute	*Beta*	absolute		*Beta*	absolute
**Vegetables total**	-0,079	0,079	0	0	**Total fat & Animal protein**	-0,141	0,141
**Raised cholesterol**	-0,071	0,071	0	0	**Total fat & Total protein**	-0,114	0,114
**% PC CARB energy**	0,066	0,066	0	0	**% CA energy**	0,084	0,084
**Cheese**	-0,065	0,065	0	0	**% PC CARB energy**	0,068	0,068
**Oilcrops total**	-0,063	0,063	0	0	**Tree nuts**	-0,061	0,061
**Health expenditure (1995-2008)**	-0,058	0,058	0	0	**Health expenditure (2008)**	-0,060	0,060
**Refined sugar**	-0,057	0,057	0	0	**Health expenditure (1995-2008)**	-0,050	0,050
**Plant fat**	-0,053	0,053	0	0	**Cheese**	-0,035	0,035
**Tree nuts**	-0,052	0,052	0	0	**Total fat**	-0,027	0,027
**Health expenditure (2008)**	-0,052	0,052	0	0	**Fruits total**	-0,026	0,026
**Rye**	0,050	0,050	0	0	**Plant fat**	-0,020	0,020
**Total fat & Animal protein**	-0,049	0,049	-0,376	0,376	**Oranges & Mandarins**	-0,004	0,004
**% CA energy**	0,049	0,049	0	0			
**Fruits total**	-0,048	0,048	0	0			
**Honey**	-0,048	0,048	0	0			
**Total fat & Total protein**	-0,048	0,048	0	0			
**Oranges & Mandarins**	-0,046	0,046	0	0			
**Cocoa beans**	-0,044	0,044	0	0			
**Distilled alcohol**	0,043	0,043	0	0			
**Lard**	0,041	0,041	0	0			
**Ref. sugar & Sweeteners total**	-0,039	0,039	0	0			
**Fish & Seafood**	0,039	0,039	0	0			
**Beer**	0,038	0,038	0	0			
**PC CARB energy**	0,038	0,038	0	0			
**Milk**	0,037	0,037	0	0			
**Sunflower oil**	0,036	0,036	0	0			
**Total energy**	-0,036	0,036	0	0			
**Poultry**	-0,035	0,035	0	0			
**Plant protein**	-0,033	0,033	0	0			
**Milk products (dairy) total**	0,031	0,031	0	0			
**CA energy**	0,031	0,031	0	0			
**Pork**	0,030	0,030	0	0			
**Total fat**	-0,030	0,030	0	0			

Supplementary Table 3b. Raised blood pressure (Men): Results of the elastic net regression (a model with the lowest prediction error).

Model	Ridge Penalty	Lasso Penalty	Regularization "R Square" (1-Error)	Number of selected predictors	Standardized sum of beta-coefficients	Apparent prediction error	Expected prediction error		
							Estimate	Std. Error	*N*
**548**	1,000	0,740	0,796	14	0,216	0,204	0,383	0,116	41

VARIABLES	*Beta*	absolute
**Total fat & Animal protein**	-0,149	0,149
**Total fat & Total protein**	-0,126	0,126
**% CA energy**	0,098	0,098
**Tree nuts**	-0,096	0,096
**% PC CARB energy**	0,095	0,095
**Health expenditure (2008)**	-0,077	0,077
**Cheese**	-0,070	0,070
**Health expenditure (1995-2008)**	-0,064	0,064
**Plant fat**	-0,050	0,050
**Fruits total**	-0,048	0,048
**Total fat**	-0,034	0,034
**Oranges & Mandarins**	-0,033	0,033
**Raised cholesterol**	-0,013	0,013
**Olive oil**	-0,003	0,003

Supplementary Table 4a. Raised blood pressure (Women): Parsimonious models of the ridge regression, LASSO regression and elastic net regression, computed by the bootstrapping method and sorted according to the absolute *beta* coefficients of the ridge regression and elastic net regression. In the ridge regression, all food items that reach a non-zero value in the elastic net regression are listed.

VARIABLES	Ridge		LASSO		VARIABLES	Elastic net	
	Beta	absolute	*Beta*	absolute		*Beta*	absolute
**Oranges & Mandarins**	-0,055	0,055	-0,069	0,069	**Health expenditure (1995-2008)**	-0,184	0,184
**Fish & Seafood fat**	-0,049	0,049	0	0	**Health expenditure (2008)**	-0,164	0,164
**Health expenditure (1995-2008)**	-0,048	0,048	-0,470	0,470	**Oranges & Mandarins**	-0,136	0,136
**Total protein**	-0,048	0,048	-0,014	0,014	**Total fat & Total protein**	-0,103	0,103
**Health expenditure (2008)**	-0,041	0,041	-0,176	0,176	**Total fat & Animal protein**	-0,080	0,080
**Meat protein**	-0,040	0,040	0	0	**% PC CARB energy**	0,079	0,079
**Animal protein**	-0,038	0,038	0	0	**Animal protein**	-0,071	0,071
**Total fat & Total protein**	-0,037	0,037	0	0	**Total fat**	-0,069	0,069
**Cheese**	-0,036	0,036	0	0	**% CA energy**	0,049	0,049
**% CA energy**	0,034	0,034	0	0	**Cheese**	-0,044	0,044
**% PC CARB energy**	0,031	0,031	0,034	0,034	**Meat protein**	-0,037	0,037
**Total fat & Animal protein**	-0,029	0,029	-0,003	0,003	**Total protein**	-0,016	0,016
**Total fat**	-0,023	0,023	0	0	**Fish & Seafood fat**	-0,013	0,013
**Fruits total**	-0,020	0,020	0	0	**Fruits total**	-0,002	0,002

Supplementary Table 4b. Raised blood pressure (Women): Results of the elastic net regression (a model with the lowest prediction error).

Model	Ridge Penalty	Lasso Penalty	Regularization "R Square" (1-Error)	Number of selected predictors	Standardized sum of beta-coefficients	Apparent prediction error	Expected prediction error		
							Estimate	Std. Error	*N*
**172**	0,300	0,360	0,910	16	0,088	0,090	0,185	0,060	42

VARIABLES	*Beta*	absolute
**Health expenditure (1995-2008)**	-0,213	0,213
**Health expenditure (2008)**	-0,163	0,163
**Oranges & Mandarins**	-0,162	0,162
**% PC CARB energy**	0,083	0,083
**Total fat & Total protein**	-0,066	0,066
**Total fat & Animal protein**	-0,056	0,056
**Cheese**	-0,048	0,048
**Animal protein**	-0,045	0,045
**Meat protein**	-0,043	0,043
**Total protein**	-0,037	0,037
**Fish & Seafood fat**	-0,035	0,035
**% CA energy**	0,034	0,034
**Total fat**	-0,020	0,020
**Oilcrops total**	-0,016	0,016
**Plant fat**	-0,004	0,004
**Refined sugar**	-0,004	0,004

Supplementary Table 5a. Actual total CVD mortality (Men): Parsimonious models of the ridge regression, LASSO regression and elastic net regression, computed by the bootstrapping method and sorted according to the absolute *beta* coefficients of the ridge regression and elastic net regression. In the ridge regression, all food items that reach a non-zero value in the elastic net regression are listed.

VARIABLES	Ridge		LASSO		VARIABLES	Elastic net	
	Beta	absolute	*Beta*	absolute		*Beta*	absolute
**Health expenditure (1995-2008)**	-0,056	0,056	-0,064	0,064	**Oranges & Mandarins**	-0,150	0,150
**Tree nuts**	-0,056	0,056	0	0	**Health expenditure (2008)**	-0,144	0,144
**Distilled beverages**	0,054	0,054	0	0	**Health expenditure (1995-2008)**	-0,142	0,142
**Olive oil**	-0,054	0,054	0	0	**Fruits total**	-0,063	0,063
**Health expenditure (2008)**	-0,053	0,053	-0,293	0,293	**Total fat**	-0,055	0,055
**Sunflower oil**	0,051	0,051	0	0	**Total fat & Animal protein**	-0,035	0,035
**Poultry**	-0,051	0,051	0	0			
**Oranges & Mandarins**	-0,050	0,050	-0,235	0,235			
**PC CARB energy**	-0,049	0,049	0	0			
**Cereals total**	-0,048	0,048	0	0			
**Raised cholesterol**	-0,048	0,048	0	0			
**% Plant food energy**	-0,047	0,047	0	0			
**Bananas**	-0,046	0,046	0	0			
**Total fat**	-0,043	0,043	0	0			
**Animal fat**	0,042	0,042	0	0			
**Soybean oil**	-0,041	0,041	0	0			
**Total fat & Animal protein**	-0,040	0,040	0	0			
**Fruits total**	-0,039	0,039	0	0			

Supplementary Table 5b. Actual total CVD mortality (Men): Results of the elastic net regression (a model with the lowest prediction error).

Model	Ridge Penalty	Lasso Penalty	Regularization "R Square" (1-Error)	Number of selected predictors	Standardized sum of beta-coefficients	Apparent prediction error	Expected prediction error		
							Estimate	Std. Error	*N*
**281**	0,500	0,500	0,869	10	0,179	0,131	0,230	0,092	41

VARIABLES	*Beta*	absolute
**Oranges & Mandarins**	-0,193	0,193
**Health expenditure (2008)**	-0,173	0,173
**Health expenditure (1995-2008)**	-0,169	0,169
**Total fat & Total protein**	-0,120	0,120
**Total fat**	-0,085	0,085
**Olive oil**	-0,078	0,078
**Fruits total**	-0,076	0,076
**Tree nuts**	-0,069	0,069
**% CA energy**	0,026	0,026
**Total energy**	-0,012	0,012

Supplementary Table 6a. Actual total CVD mortality (Women): Parsimonious models of the ridge regression, LASSO regression and elastic net regression, computed by the bootstrapping method and sorted according to the absolute *beta* coefficients of the ridge regression and elastic net regression. In the ridge regression, all food items that reach a non-zero value in the elastic net regression are listed.

VARIABLES	Ridge		LASSO		VARIABLES	Elastic net	
	Beta	absolute	*Beta*	absolute		*Beta*	absolute
**BMI (1990-2008)**	0,101	0,101	0	0	**Oranges & Mandarins**	-0,143	0,143
**Smoking (1990-2009)**	-0,087	0,087	-0,079	0,079	**Fruits total**	-0,133	0,133
**Grapes**	-0,080	0,080	0	0	**Smoking (1990-2009)**	-0,127	0,127
**Alcoholic beverages total**	-0,078	0,078	0	0	**BMI (1990-2008)**	0,123	0,123
**Eggs total**	0,073	0,073	0	0	**Alcoholic beverages total**	-0,116	0,116
**Coffee**	-0,072	0,072	-0,027	0,027	**Coffee**	-0,114	0,114
**Fruits total**	-0,066	0,066	-0,044	0,044	**Cheese**	-0,092	0,092
**Cheese**	-0,064	0,064	0	0	**Potatoes**	0,072	0,072
**Oranges & Mandarins**	-0,063	0,063	-0,077	0,077	**Total protein**	-0,069	0,069
**Wine**	-0,063	0,063	0	0	**Wine**	-0,028	0,028
**Cereals total**	-0,061	0,061	0	0	**Tree nuts**	-0,006	0,006
**Potatoes**	0,059	0,059	0	0	**Total fat**	-0,006	0,006
**Total protein**	-0,059	0,059	0	0			
**Fish & Seafood**	0,054	0,054	0	0			
**PC CARB energy**	-0,052	0,052	0	0			
**Tree nuts**	-0,051	0,051	0	0			
**Total energy**	-0,051	0,051	0	0			
**Honey**	0,050	0,050	0	0			
**Health expenditure (1995-2008)**	-0,050	0,050	0	0			
**Total fat**	-0,049	0,049	0	0			

Supplementary Table 6b. Actual total CVD mortality (Women): Results of the elastic net regression (a model with the lowest prediction error).

Model	Ridge Penalty	Lasso Penalty	Regularization "R Square" (1-Error)	Number of selected predictors	Standardized sum of beta-coefficients	Apparent prediction error	Expected prediction error		
							Estimate	Std. Error	*N*
**259**	0,500	0,060	0,905	53	0,637	0,095	0,523	0,176	36

VARIABLES	*Beta*	absolute	VARIABLES	*Beta*	absolute
**Smoking (1990-2009)**	-0,174	0,174	**Soybean oil**	-0,044	0,044
**BMI (1990-2008)**	0,161	0,161	**Rye**	0,044	0,044
**Alcoholic beverages total**	-0,159	0,159	**% CA energy**	0,039	0,039
**Cheese**	-0,153	0,153	**Onions**	0,036	0,036
**Coffee**	-0,145	0,145	**Beef & pork fat**	0,036	0,036
**Eggs total**	0,144	0,144	**Maize**	-0,036	0,036
**Potatoes**	0,139	0,139	**Edible offals**	0,033	0,033
**Grapes**	-0,137	0,137	**Plant protein**	-0,031	0,031
**Wine**	-0,135	0,135	**Refined sugar**	0,030	0,030
**Honey**	0,114	0,114	**% PC CARB energy**	0,028	0,028
**Total protein**	-0,112	0,112	**Distilled alcohol**	0,027	0,027
**Cereals total**	-0,102	0,102	**% Plant food energy**	0,025	0,025
**Total energy**	-0,098	0,098	**Dairy protein**	0,025	0,025
**Health expenditure (1995-2008)**	-0,097	0,097	**CA energy**	0,024	0,024
**Total fat**	-0,096	0,096	**Butter & Ghee**	0,023	0,023
**Fish & Seafood**	0,094	0,094	**Poultry**	-0,019	0,019
**Tree nuts**	-0,090	0,090	**Plant oils total**	0,016	0,016
**Fish & Seafood fat**	0,077	0,077	**Total fat & Animal protein**	-0,014	0,014
**Sunflower oil**	0,071	0,071	**Total fat & Total protein**	-0,013	0,013
**Health expenditure (2008)**	-0,069	0,069	**Wheat**	-0,012	0,012
**Legumes total**	-0,064	0,064	**Refined sugar&Sweeteners total**	0,005	0,005
**Beer**	-0,062	0,062	**Meat fat**	0,004	0,004
**Oilcrops total**	0,059	0,059	**Tomatoes**	0,001	0,001
**PC CARB energy**	-0,057	0,057	**Meat protein**	-0,001	0,001
**Pork**	-0,056	0,056	**Meat total**	-0,001	0,001
**Milk**	0,048	0,048	**Dairy fat**	0,001	0,001
**Lard**	0,045	0,045			

Supplementary Table 7. Actual total CVD mortality in men: The first row displays results of the dependent t-test (standard deviations of the mean difference between pairs of r-values). The lower the number, the closer the temporal relationship between two trend lines. The second row displays results of the regression slope test (probability p-values expressing the similarity between linear slopes of two trend lines). The higher the p-value, the higher the probability that two linear trend lines are running parallel to each other. (See Figs. S23-S24)

	**Health expenditure**	**TF & AP**	**Fruits**	**Bananas**	**Oranges & Mandarins**	**Wine**	**Coffee**	**Cheese**	**% CA energy**	**Cereals**	**Potatoes**	**Distilled beverages**	**Onions**
**Health exp.**	-												
**TF & AP**	0.027<br>p=0.38	-											
**Fruits**	0.038<br>p=0.05	0.038<br>p=0.05	-										
**Bananas**	0.031<br>p=0.01	0.087<br>p=0.01	0.079<br>p=0.27	-									
**Or & Mand.**	0.071	0.090	0.060	0.070<br>p=0.14	-								
**Wine**	0.045	0.056	0.075	0.102	0.122	-							
**Coffee**	0.067	0.068	0.051<br>p=0.01	0.074<br>p=0.29	0.059<br>p=0.54	0.111	-						
**Cheese**	0.025	0.026<br>p=0.05	0.027<br>p=0.05	0.073	0.072	0.067	0.052	-					
**%CA energy**	0.021<br>p=0.12	0.049<br>p=0.87	0.052<br>p=0.01	0.060<br>p=0.01	0.084	0.047	0.075	0.043	-				
**Cereals**	0.065	0.078	0.100	0.129	0.148	0.033	0.135	0.093	0.076	-			
**Potatoes**	0.078	0.088	0.075	0.080<br>p=0.05	0.074<br>p=0.61	0.127	0.049<br>p=0.24	0.071	0.091	0.156	-		
**Dist. bever.**	0.092<br>p=0.48	0.072<br>p=0.75	0.100<br>p=0.18	0.135<br>p=0.06	0.150	0.096<br>p=0.21	0.109<br>p=0.01	0.089<br>p=0.12	0.104<br>r=0.69	0.107<br>p=0.03	0.122	-	
**Onions**	0.107	0.112	0.101	0.108<br>p=0.04	0.094<br>p=0.32	0.151	0.075<br>p=0.14	0.099	0.119	0.178	0.050<br>p=0.51	0.138	-
**Sunfl. oil**	0.097	0.109	0.084	0.096	0.060<br>p=0.03	0.151	0.063	0.091	0.113	0.179	0.048<br>p=0.09	0.148	0.069<br>p=0.60

Abbreviations: TF & AP = Total fat & Animal protein, Or & Mand = Oranges & Mandarins, % CA energy = % energy from carbohydrates and alcohol in the diet.

Supplementary Table 8. Actual total CVD mortality in women: The first row displays results of the dependent t-test (standard deviations of the mean difference between pairs of r-values). The lower the number, the closer the temporal relationship between two trend lines. The second row displays results of the regression slope test (probability p-values expressing the similarity between linear slopes of two trend lines). The higher the p-value, the higher the probability that two linear trend lines are running parallel to each other. (See Figs. 19-20)

	**Health expenditure**	**TF & AP**	**Fruits**	**Bananas**	**Oranges & Mandarins**	**Wine**	**Coffee**	**Cheese**	**% CA energy**	**Cereals**	**Potatoes**	**Distilled beverages**	**Onions**
**Health exp.**	-												
**TF & AP**	0.021<br>p=0.52	-											
**Fruits**	0.031<br>p=0.20	0.028<br>p=0.16	-										
**Bananas**	0.032	0.078	0.073<br>p=0.05	-									
**Or & Mand.**	0.062	0.079	0.061	0.062<br>p=0.41	-								
**Wine**	0.056	0.062	0.078	0.114	0.124	-							
**Coffee**	0.072	0.072	0.057	0.071<br>p=0.29	0.058<br>p=0.81	0.125	-						
**Cheese**	0.024	0.019	0.026<br>p=0.98	0.069	0.068	0.076	0.059	-					
**% CA energy**	0.015<br>p=0.18	0.036<br>p=0.99	0.040<br>p=0.09	0.062<br>p=0.09	0.071	0.060	0.076	0.037	-				
**Cereals**	0.053	0.063	0.078	0.120	0.127	0.020<br>p=0.90	0.126	0.077	0.065	-			
**Potatoes**	0.089	0.100	0.090	0.079<br>p=0.01	0.079<br>p=0.06	0.152	0.055<br>p=0.10	0.085	0.099	0.156	-		
**Dist. bev.**	0.070<br>p=0.37	0.056<br>p=0.53	0.070<br>p=0.18	0.113<br>p=0.02	0.122	0.080<br>p=0.03	0.097	0.066<br>p=0.08	0.079<br>p=0.51	0.082<br>p=0.03	0.124	-	
**Onions**	0.084	0.096	0.090	0.079<br>p=0.28	0.088<br>p=0.64	0.141	0.069<br>p=0.77	0.085	0.094	0.146	0.047<br>p=0.33	0.119	-
**Sunflower oil**	0.098	0.114	0.098	0.085	0.067	0.168	0.061	0.098	0.112	0.171	0.042<br>p=0.16	0.140	0.078<br>p=0.04

Abbreviations: TF & AP = Total fat & Animal protein, Or & Mand = Oranges & Mandarins, % CA energy = % energy from carbohydrates and alcohol in the diet.

Supplementary Table 9. Raised blood pressure in men: The first row displays results of the dependent t-test (standard deviations of the mean difference between pairs of r-values). The lower the number, the closer the temporal relationship between two trend lines. The second row displays results of the regression slope test (probability p-values expressing the similarity between linear slopes of two trend lines). The higher the p-value, the higher the probability that two linear trend lines are running parallel to each other. (see Figs. S25-S26)

	**Health exp.**	**TF & AP**	**Fruits**	**Or & Mand.**	**Wine**	**Coffee**	**Cheese**	**Tree nuts**	**Cereals**	**Sunfl. oil**	**Distilled beverages**	**Onions**	**Potatoes**
**Health exp.**	-												
**TF & AP**	0.011<br>p=0.04	-											
**Fruits**	0.044	0.069	-										
**Or & Mand.**	0.077	0.092	0.043<br>p=0.10	-									
**Wine**	0.093	0.098	0.158	0.184	-								
**Coffee**	0.057	0.061	0.063<br>p=0.35	0.070<br>p=0.02	0.147	-							
**Cheese**	0.025<br>p=0.93	0.028<br>p=0.20	0.064	0.085	0.110	0.060	-						
**Tree nuts**	0.044<br>p=0.33	0.045<br>p=0.76	0.065	0.093	0.104	0.086<br>p=0.01	0.040<br>p=0.29	-					
**Cereals**	0.070	0.070	0.127	0.155	0.048	0.113	0.080	0.083	-				
**Sunfl. oil**	0.048<br>p=0.09	0.044	0.071<br>p=0.05	0.080	0.129	0.061<br>p=0.36	0.058	0.075<br>p=0.06	0.102	-			
**Dist. bev.**	0.114<br>p=0.58	0.104<br>p=0.76	0.144<br>p=0.04	0.166<br>p=0.01	0.132	0.125<br>p=0.12	0.124<br>p=0.49	0.135<br>p=0.86	0.104<br>p=0.10	0.106<br>p=0.26	-		
**Onions**	0.070	0.077	0.068<br>p=0.87	0.075<br>p=0.15	0.159	0.076<br>p=0.56	0.085	0.091	0.128	0.063<br>p=0.17	0.124<br>p=0.07	-	
**Potatoes**	0.033<br>p=0.90	0.046<br>p=0.43	0.062	0.090	0.115	0.077<br>p=0.05	0.048<br>p=0.85	0.046<br>p=0.43	0.087	0.064<br>p=0.24	0.121<br>p=0.57	0.075<br>p=0.02	-
**% CA energy**	0.036	0.035<br>p=0.01	0.085	0.115	0.083	0.076	0.046	0.055<br>p=0.21	0.045	0.070	0.097<br>p=0.73	0.093	0.051<br>p=0.03

Abbreviations: TF & AP = Total fat & Animal protein, Or & Mand = Oranges & Mandarins, % CA energy = % energy from carbohydrates and alcohol in the diet.

Supplementary Table 10. Raised blood pressure in women: The first row displays results of the dependent t-test (standard deviations of the mean difference between pairs of r-values). The lower the number, the closer the temporal relationship between two trend lines. The second row displays results of the regression slope test (probability p-values expressing the similarity between linear slopes of two trend lines). The higher the p-value, the higher the probability that two linear trend lines are running parallel to each other. (see Figs. 21-22)

	**Health exp.**	**TF & AP**	**Fruits**	**Or & Mand.**	**Wine**	**Coffee**	**Cheese**	**Tree nuts**	**Cereals**	**Sunfl. oil**	**Distilled beverages**	**Onions**	**Potatoes**
**Health exp.**	-												
**TF & AP**	0.011<br>p=0.82	-											
**Fruits**	0.043	0.051	-										
**Or & Mand.**	0.054	0.060	0.043<br>p=0.99	-									
**Wine**	0.102	0.119	0.161	0.166	-								
**Coffee**	0.079	0.075	0.053<br>p=0.07	0.061<br>p=0.11	0.186	-							
**Cheese**	0.018	0.018	0.060	0.066	0.113	0.086	-						
**Tree nuts**	0.042<br>p=0.56	0.042<br>p=0.62	0.064	0.061	0.122	0.100	0.040<br>p=0.75	-					
**Cereals**	0.063	0.065	0.105	0.113	0.064	0.132	0.057	0.077	-				
**Sunfl. oil**	0.069	0.074	0.050<br>p=0.02	0.055<br>p=0.05	0.188	0.049<br>p=0.84	0.085	0.085	0.136	-			
**Dist. bev.**	0.094<br>p=0.15	0.089<br>p=0.16	0.115	0.138<br>p=0.01	0.113	0.129	0.094<br>p=0.16	0.118<br>p=0.27	0.083<br>p=0.31	0.138	-		
**Onions**	0.062<br>p=0.02	0.068<br>p=0.02	0.064	0.080<br>p=0.93	0.164	0.077<br>p=0.15	0.077	0.086<br>p=0.02	0.113	0.070<br>p=0.09	0.115<br>p=0.02	-	
**Potatoes**	0.039	0.058	0.047<br>p=0.76	0.060<br>p=0.78	0.160	0.072<br>p=0.06	0.064	0.066<br>p=0.01	0.108	0.052<br>p=0.02	0.121<br>p=0.01	0.045<br>p=0.88	-
**% CA energy**	0.028	0.031	0.070	0.081	0.094	0.099	0.027	0.050<br>p=0.06	0.040	0.100	0.082<br>p=0.73	0.083	0.072

Abbreviations: TF & AP = Total fat & Animal protein, Or & Mand = Oranges & Mandarins, % CA energy = % energy from carbohydrates and alcohol in the diet.

Supplementary Table 11. Comparison of CVD statistics from 1980 and 2000. Changes in CVD mortality rates between 1980 and 2000, and changes in the CHD / Stroke mortality ratio between 1980 and 2000.

	CHD mortality in 2000 (as a ratio of 1980)		Stroke mortality in 2000 (as a ratio of 1980)		Total CHD & stroke mortality (as a ratio of 1980)		CHD / Stroke mortality ratio (Men)		CHD / Stroke mortality ratio (Women)	
	Men	Women	Men	Women	Men	Women	1980	2000	1980	2000
Armenia	1.11	0.85	1.07	1.18	1.10	0.96	3.85	3.97	1.94	1.40
Austria	0.57	0.50	0.41	0.50	0.53	0.50	3.07	4.27	1.43	1.43
Azerbaijan	1.16	1.27	0.71	0.81	1.03	1.08	2.60	4.27	1.41	2.20
Belarus	1.51	1.29	1.25	1.11	1.43	1.21	2.32	2.80	1.20	1.38
Belgium	0.44	0.44	0.53	0.58	0.46	0.50	4.05	3.40	1.50	1.14
Bulgaria	1.09	1.05	1.03	0.79	1.07	0.88	1.26	1.33	0.58	0.77
Estonia	0.81	0.77	1.02	0.66	0.86	0.72	3.15	2.49	1.11	1.30
France	0.54	0.43	0.40	0.50	0.49	0.47	1.95	2.63	0.70	0.60
Georgia	0.94	0.85	0.99	0.79	0.95	0.83	2.01	1.91	1.23	1.32
Greece	0.94	0.90	0.77	0.50	0.89	0.65	2.45	3.00	0.63	1.13
Hungary	0.83	0.86	0.80	0.61	0.82	0.73	2.13	2.20	0.97	1.36
Iceland	0.33	1.08	0.25	0.77	0.32	0.92	7.31	9.75	1.00	1.40
Ireland	0.42	0.29	0.42	0.45	0.42	0.35	5.92	6.00	1.86	1.20
Italy	0.44	0.38	0.36	0.40	0.41	0.39	2.48	3.00	0.87	0.83
Latvia	0.83	0.72	0.85	0.94	0.84	0.81	2.83	2.79	1.42	1.10
Lithuania	0.92	0.70	0.73	0.62	0.87	0.66	3.07	3.87	1.27	1.44
Luxembourg	0.41	0.22	0.40	0.65	0.41	0.42	3.40	3.50	1.15	0.38
Malta	0.34	0.35	0.22	0.44	0.31	0.37	4.06	6.29	3.06	2.43
Moldova	1.09	0.89	1.19	1.58	1.12	1.13	2.37	2.16	1.92	1.08
Netherlands	0.37	0.47	0.60	0.64	0.40	0.53	6.13	3.78	1.73	1.29
Poland	0.84	0.94	1.14	1.00	0.91	0.97	3.32	2.44	1.06	1.00
Portugal	0.54	0.58	0.47	0.40	0.50	0.45	0.91	1.04	0.40	0.58
Romania	1.55	1.33	1.41	1.09	1.49	1.19	1.41	1.55	0.73	0.89
Russia	1.33	1.15	1.36	1.22	1.34	1.18	2.35	2.29	1.15	1.08
Spain	0.68	0.56	0.42	0.33	0.59	0.42	1.83	3.00	0.60	1.00
Ukraine	1.40	1.23	1.28	1.11	1.36	1.18	2.57	2.81	1.49	1.64
United Kingdom	0.38	0.40	0.48	0.47	0.40	0.43	6.22	5.00	2.00	1.71

Note: Changes in CVD mortality rates between 1980 and 2000

+41% and more	+21-40%	+1-20%	No change	-1-20%	-21-40%	-41 % and less

Source: After Nichols et al. [9].

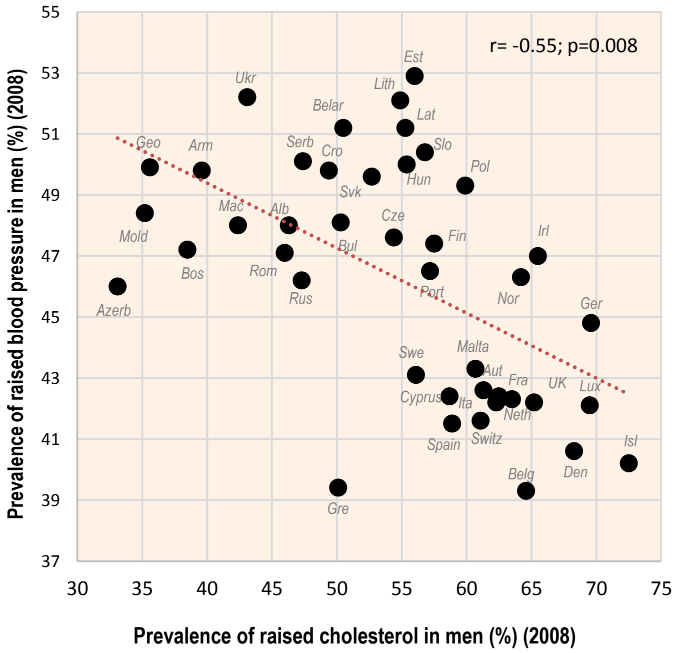

Supplementary Fig. 1. Correlation between the prevalence of raised blood pressure and the prevalence of raised cholesterol levels in men (r= -0.55; p<0.001).

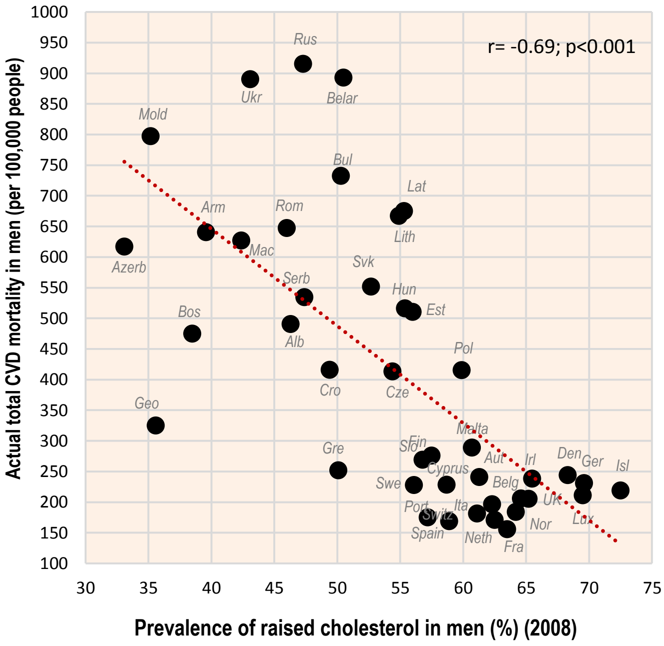

Supplementary Fig. 2. Correlation between the actual total CVD mortality and the prevalence of raised cholesterol levels in men (r= -0.69; p<0.001).

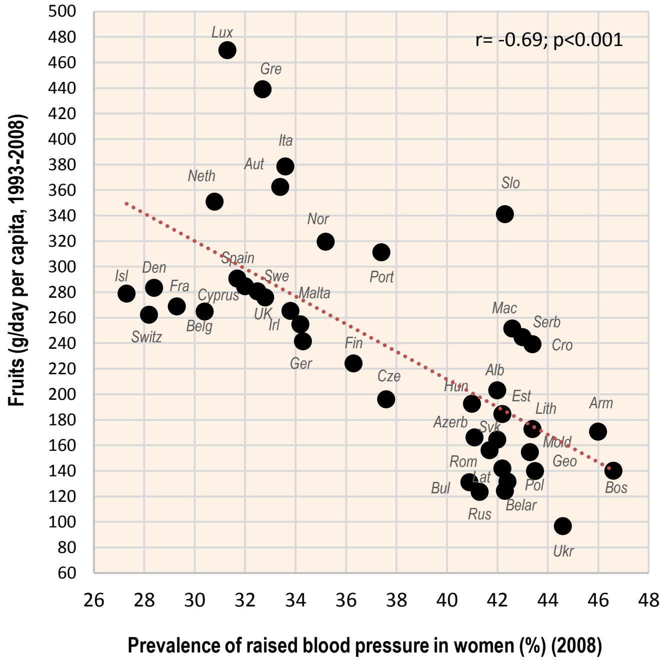

Supplementary Fig. 3. Correlation between the mean daily consumption of fruits and the prevalence of raised blood pressure in women (r= -0.69; p<0.001).

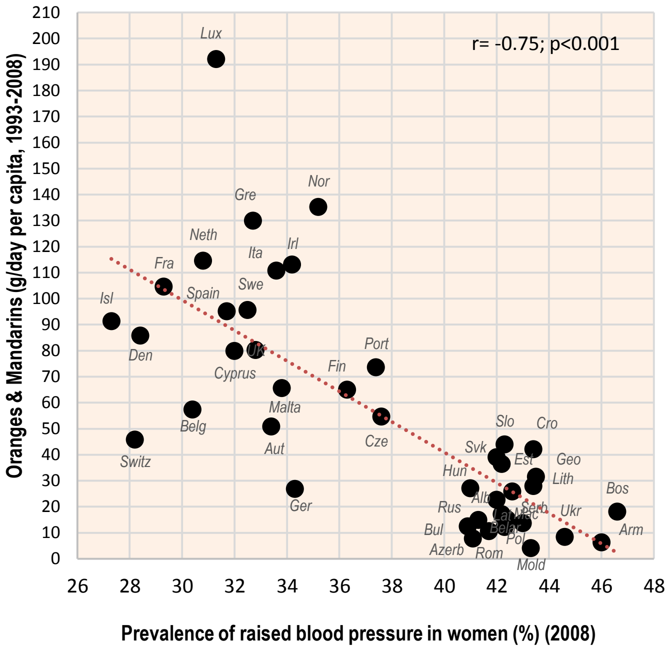

Supplementary Fig. 4. Correlation between the mean daily consumption of oranges & mandarins and the prevalence of raised blood pressure in women (r= -0.75; p<0.001).

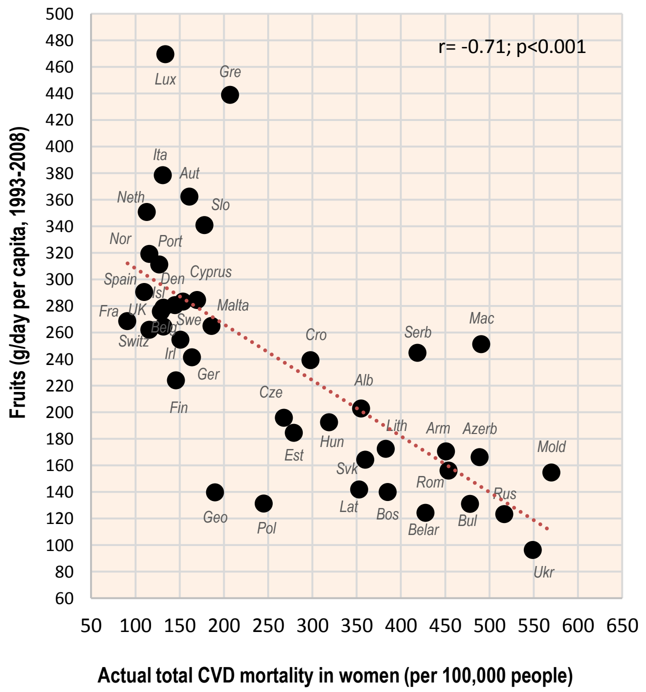

Supplementary Fig. 5. Correlation between the mean daily consumption of fruits and the actual total CVD mortality in women (r= -0.71; p<0.001).

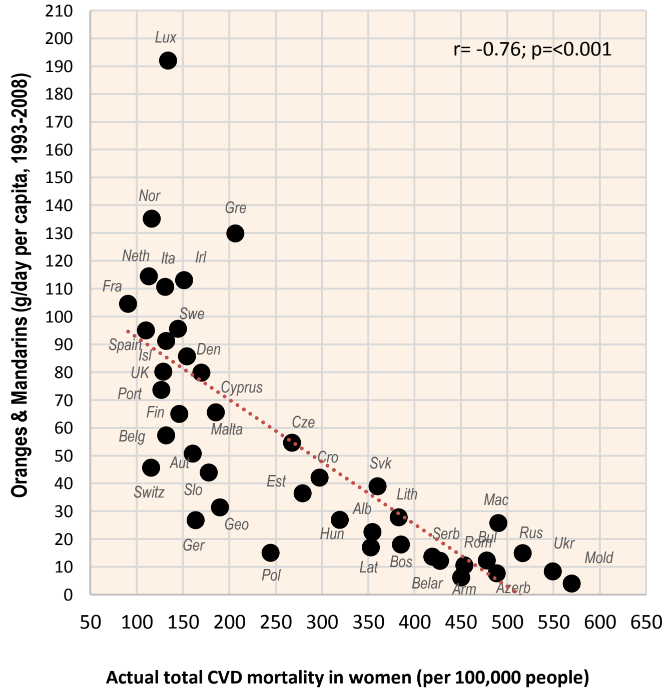

Supplementary Fig. 6. Correlation between the mean daily consumption of oranges & mandarins and the actual total CVD mortality in women (r= -0.76; p=0.001).

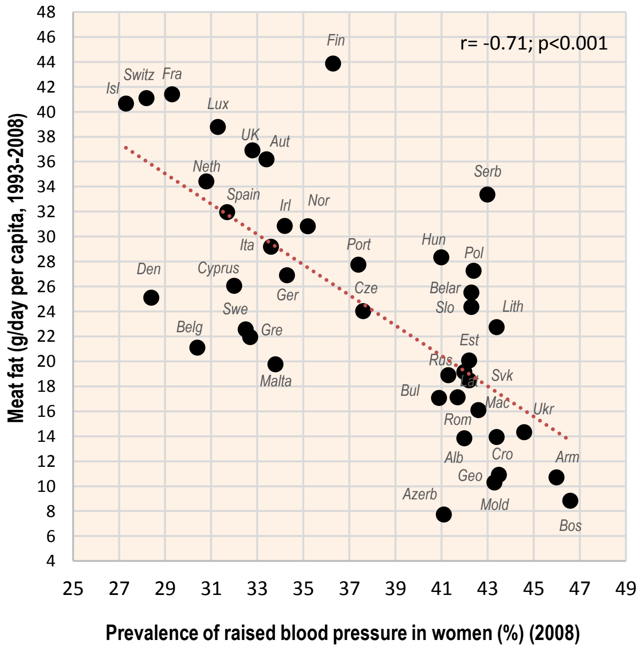

Supplementary Fig. 7. Correlation between the mean daily consumption of meat fat and the prevalence of raised blood pressure in women (r= -0.71; p=0.001).

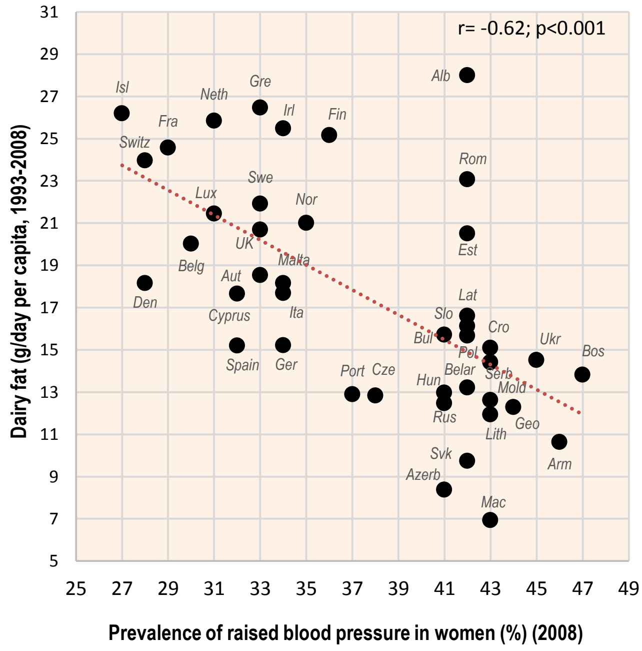

Supplementary Fig. 8. Correlation between the mean daily consumption of dairy fat and the prevalence of raised blood pressure in women (r= -0.62; p=0.001).

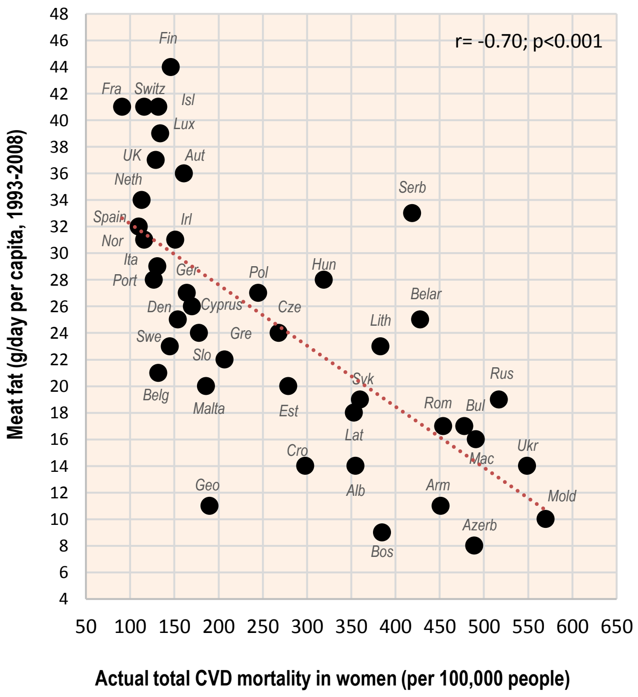

Supplementary Fig. 9. Correlation between the mean daily consumption of meat fat and the actual total CVD mortality in women (r= -0.70; p<0.001).

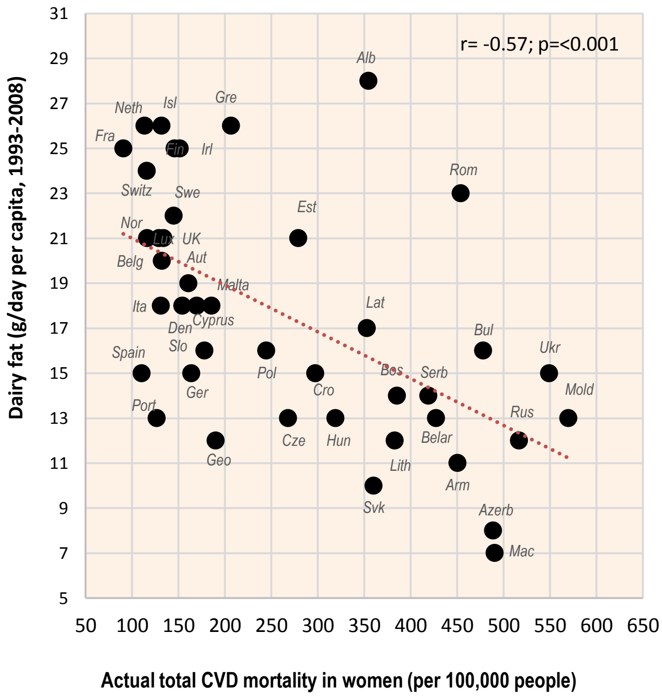

Supplementary Fig. 10. Correlation between the mean daily consumption of dairy fat and the actual total CVD mortality in women (r= -0.57; p=0.001).

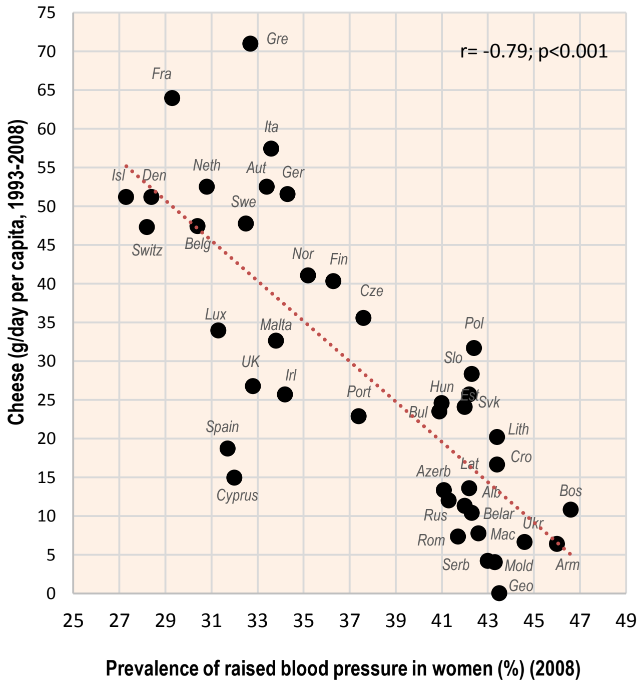

Supplementary Fig. 11. Correlation between the mean daily consumption of cheese and the prevalence of raised blood pressure in women (r= -0.79; p=0.001).

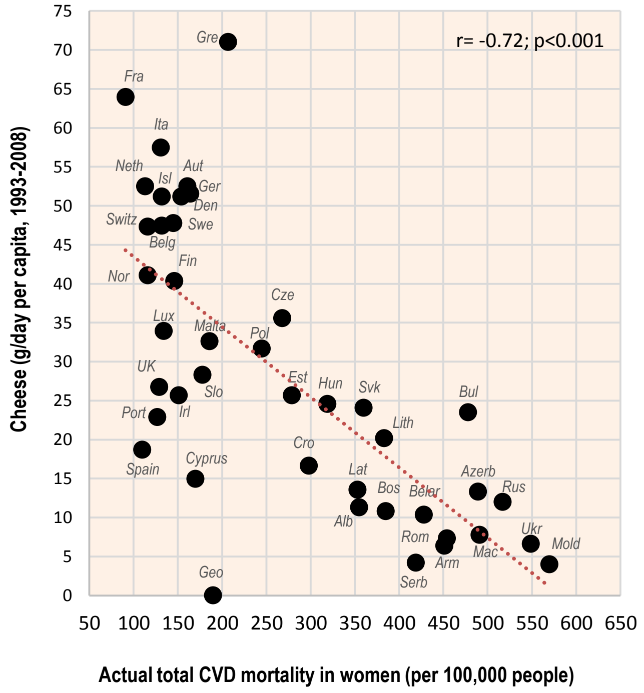

Supplementary Fig. 12. Correlation between the mean daily consumption of cheese and the actual total CVD mortality in women (r= -0.72; p<0.001).

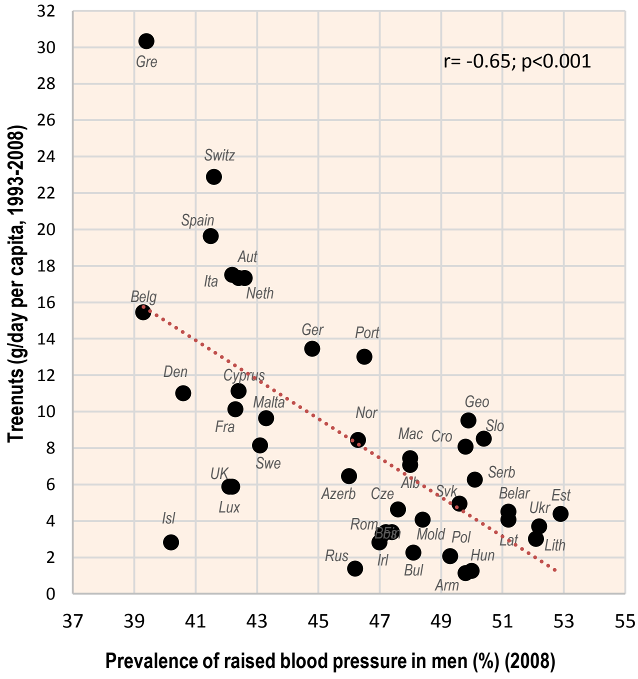

Supplementary Fig. 13. Correlation between the mean daily consumption of tree nuts and the prevalence of raised blood pressure in men(r= -0.65; p<0.001).

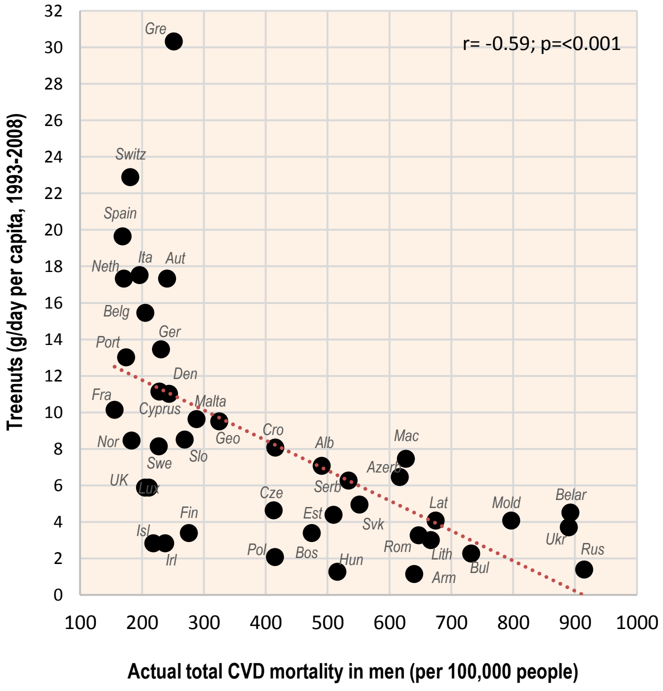

Supplementary Fig. 14. Correlation between the mean daily consumption of tree nuts and the actual total CVD mortality in men (r= -0.59; p=0.001).

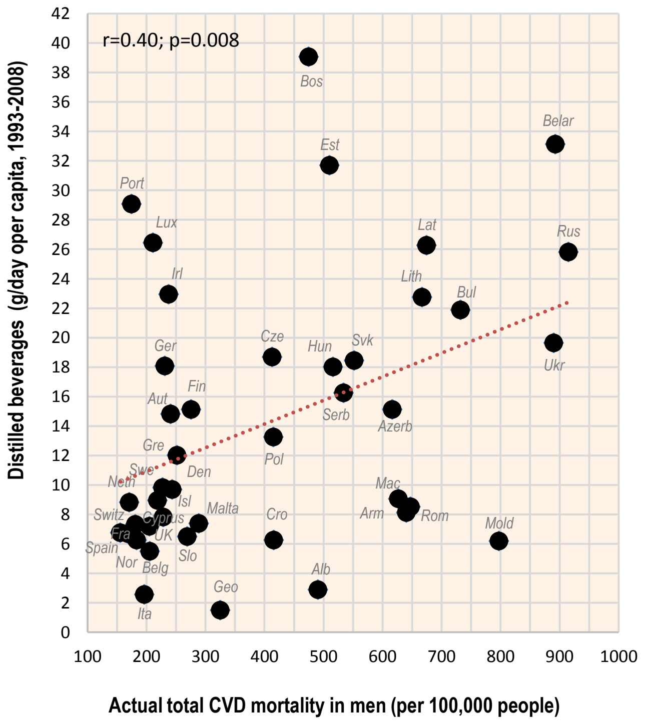

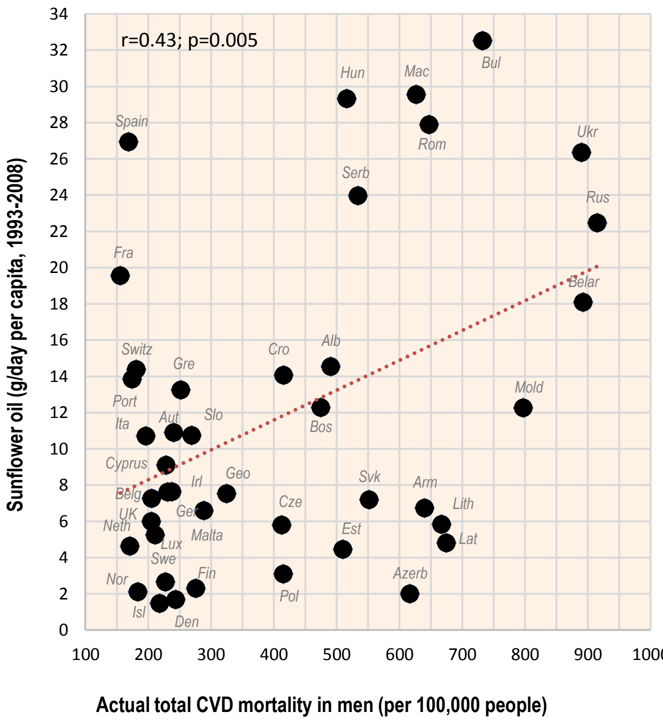

Supplementary Fig. 15. Correlation between the mean daily consumption of distilled beverages (1993-2008) and the actual total CVD mortality in men (r=0.40; p=0.008).

Supplementary Fig. 16. Correlation between the mean daily consumption of sunflower oil (1993-2008) and the actual total CVD mortality in men (r=0.43; p=0.005).

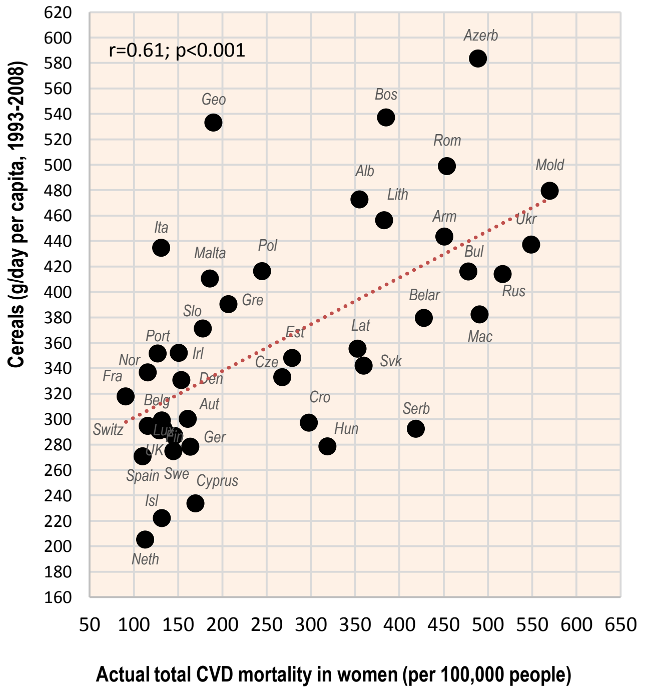

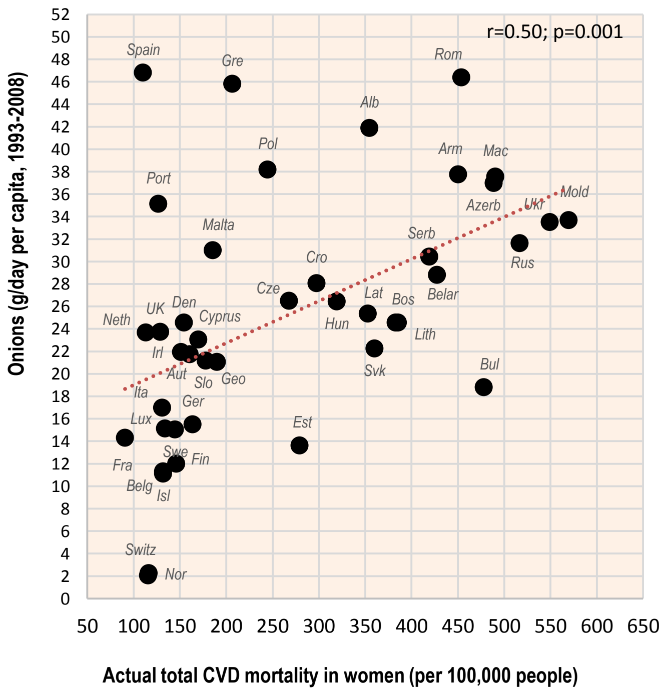

Supplementary Fig. 17. Correlation between the mean consumption of cereals (1993-2008) and the actual total CVD mortality in women (r=0.61; p<0.001).

Supplementary Fig. 18. Correlation between the mean daily consumption of onions (1993-2008) and the actual total CVD mortality in women (r=0.50; p=0.001).

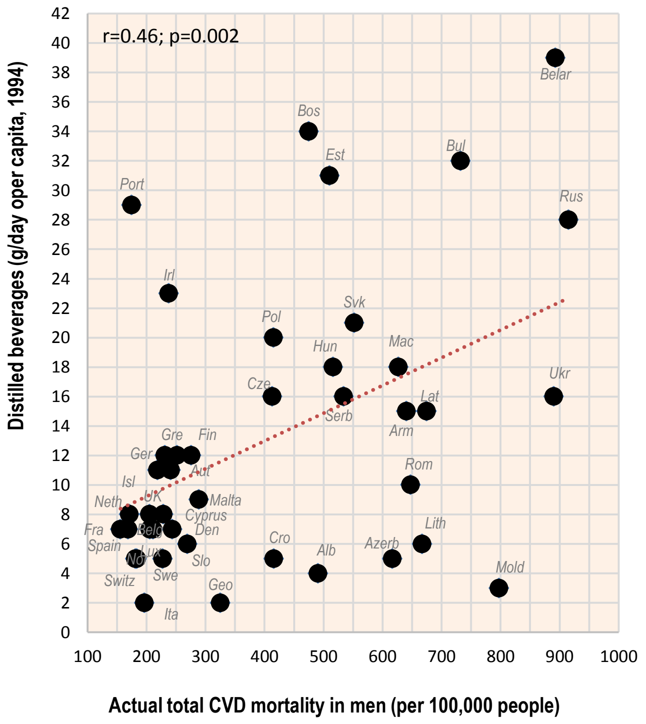

Supplementary Fig. 19. Correlation between the mean daily consumption of distilled beverages (1994) and the actual total CVD mortality in men (r=0.46; p=0.002).

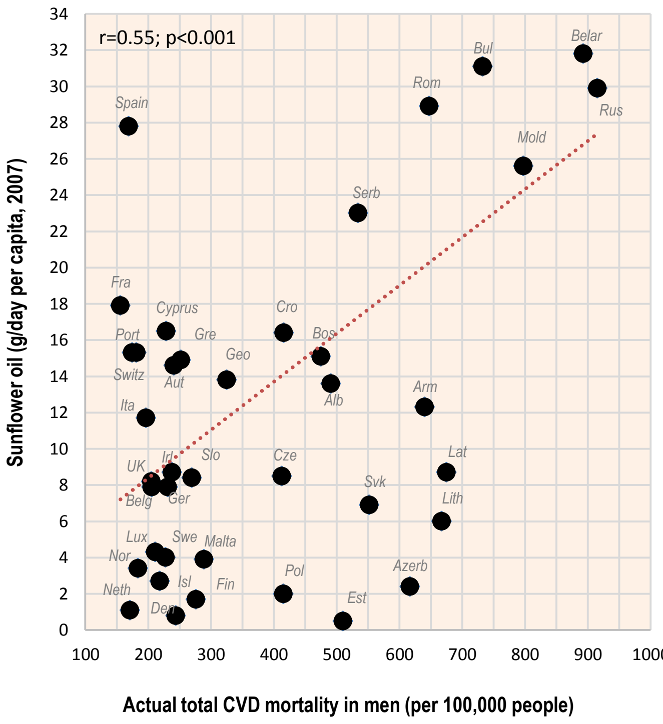

Supplementary Fig. 20. Correlation between the mean daily consumption of sunflower oil (2007) and the actual total CVD mortality in men (r=0.55; p<0.001).

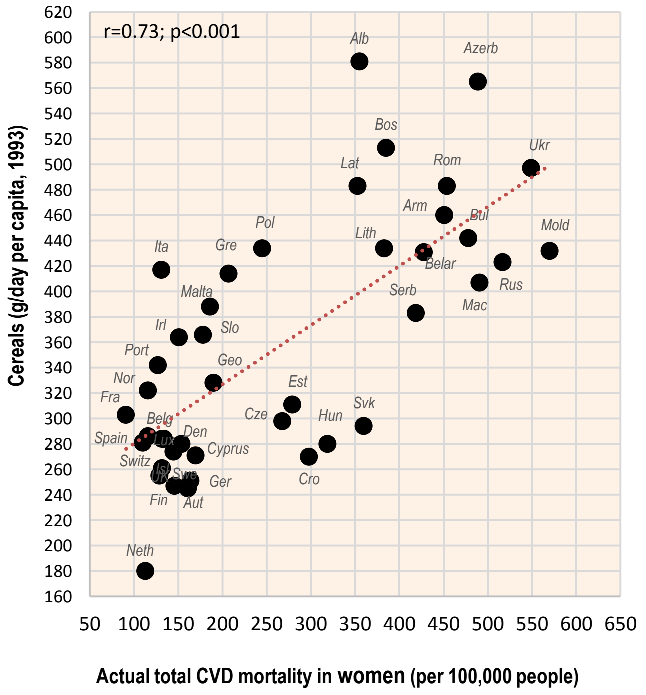

Supplementary Fig. 21. Correlation between the mean daily consumption of cereals (1993) and the actual total CVD mortality in women (r=0.73; p<0.001).

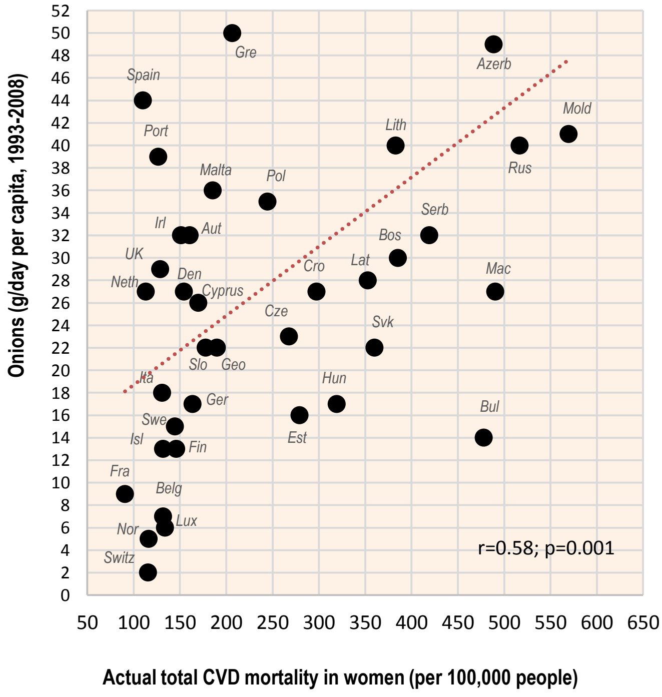

Supplementary Fig. 22. Correlation between the mean daily consumption of onions (2008) and the actual total CVD mortality in women (r=0.58; p=0.001).

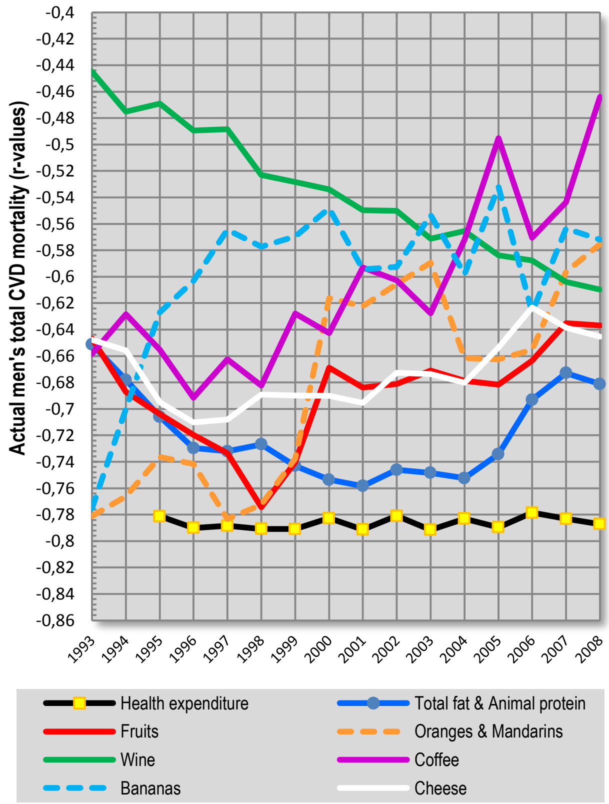

Supplementary Fig. 23. Temporal changes in the relationship among 7 negative correlates of total CVD mortality (men).

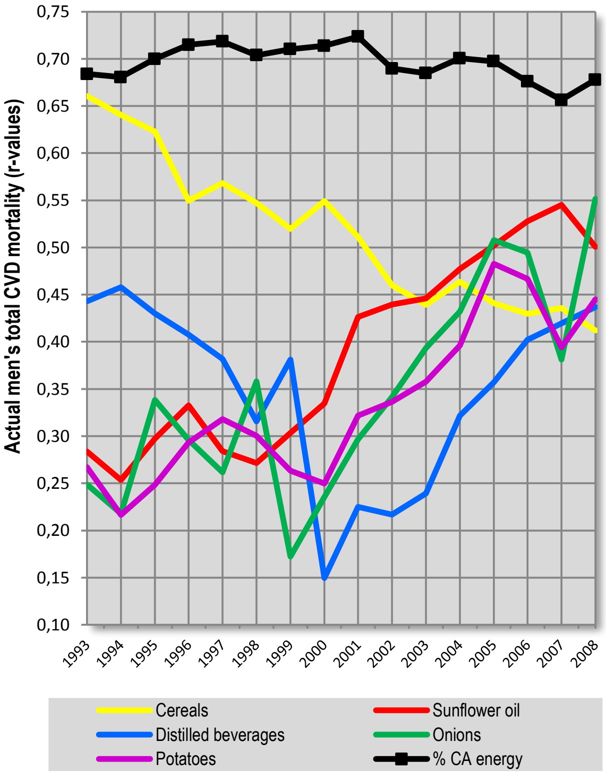

Supplementary Fig. 24. Temporal changes in the relationship among 6 positive correlates of total CVD mortality (men).

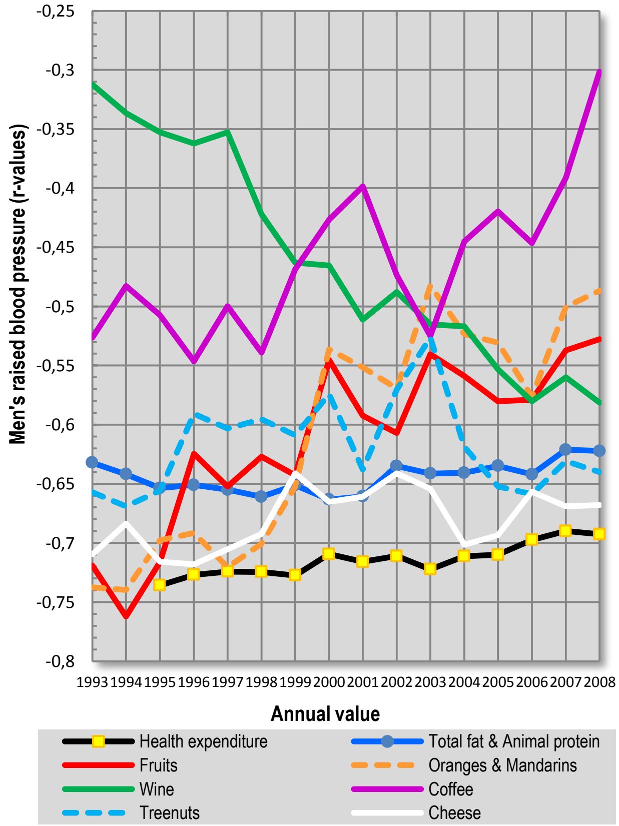

Supplementary Fig. 25. Temporal changes in the correlation among 8 negative correlates of raised blood pressure (men).

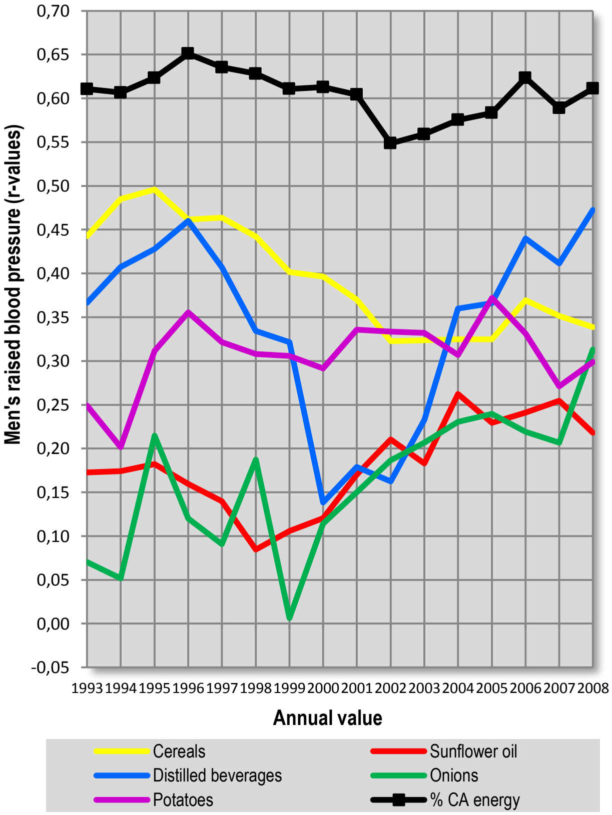

Supplementary Fig. 26. Temporal changes in the correlation among 6 positive correlates of raised blood pressure (men, 42 countries, excluding Turkey).

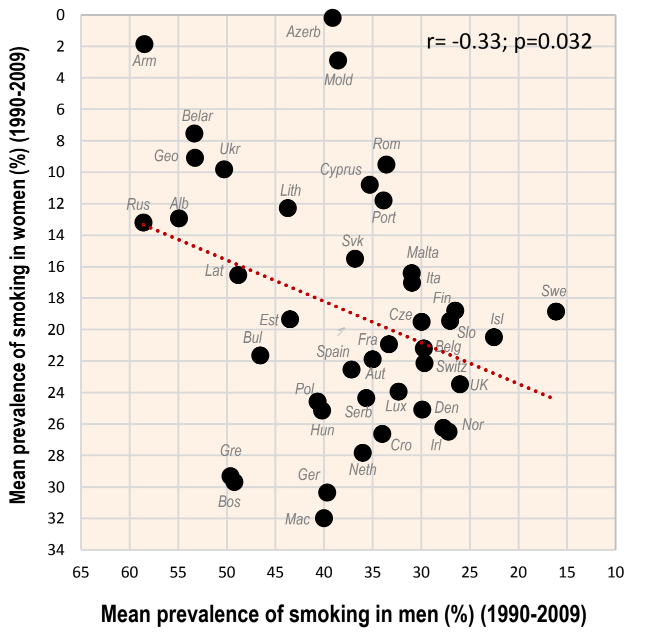

Supplementary Fig. 27. Correlation between the mean prevalence of smoking in men and women (r= -0.33; p=0.032).

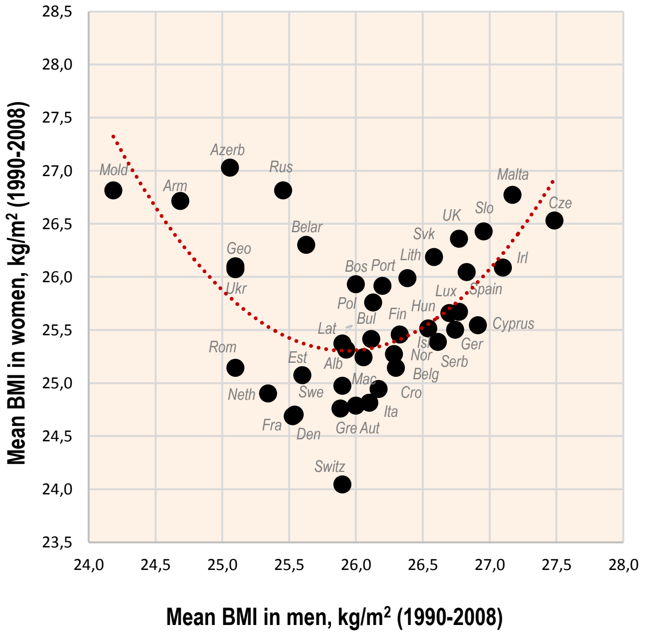

Supplementary Fig. 28. Relationship between the mean BMI in men and women.

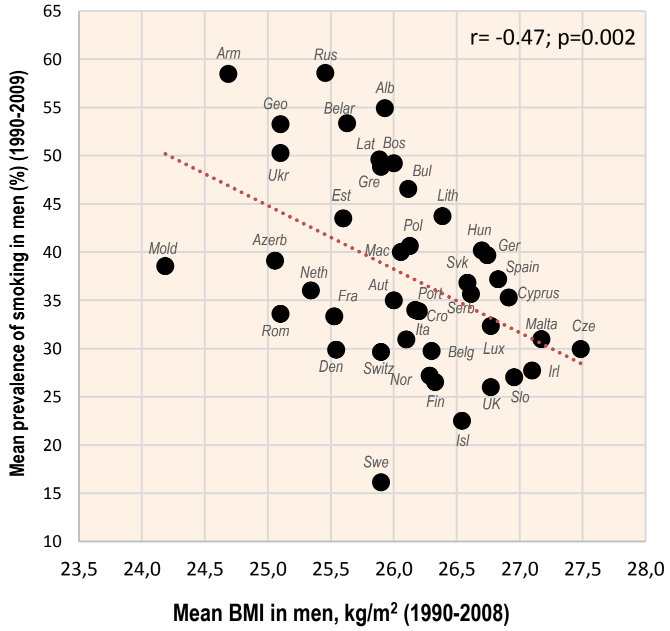

Supplementary Fig. 29. Correlation between the mean prevalence of smoking and the mean BMI in men (r= -0.47; p=0.002).

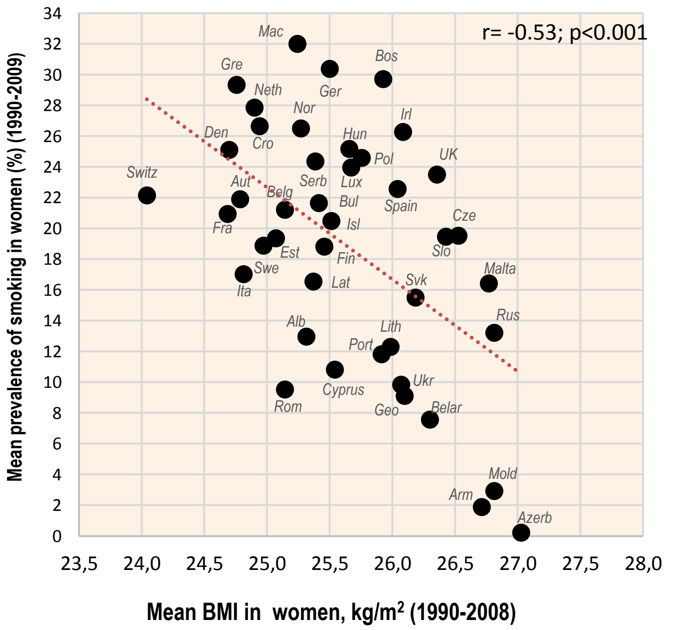

Supplementary Fig. 30. Correlation between the mean prevalence of smoking and the mean BMI in women (r= -0.53; p<0.001).

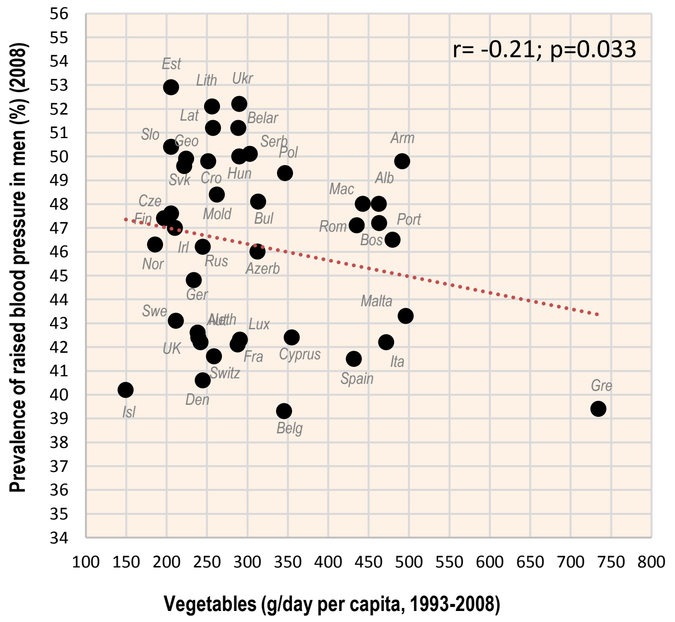

Supplementary Fig. 31. Correlation between the mean consumption of vegetables and the prevalence of raised blood pressure in men (r= -0.21; p=0.033).

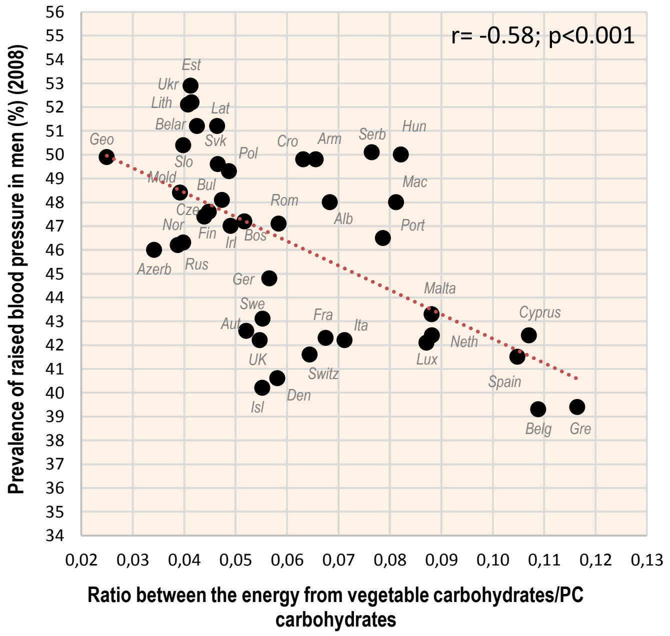

Supplementary Fig. 32. Correlation between the mean vegetable carbohydrates/PC CARB energy ratio and the prevalence of raised blood pressure in men (r= -0.58; p<0.001).

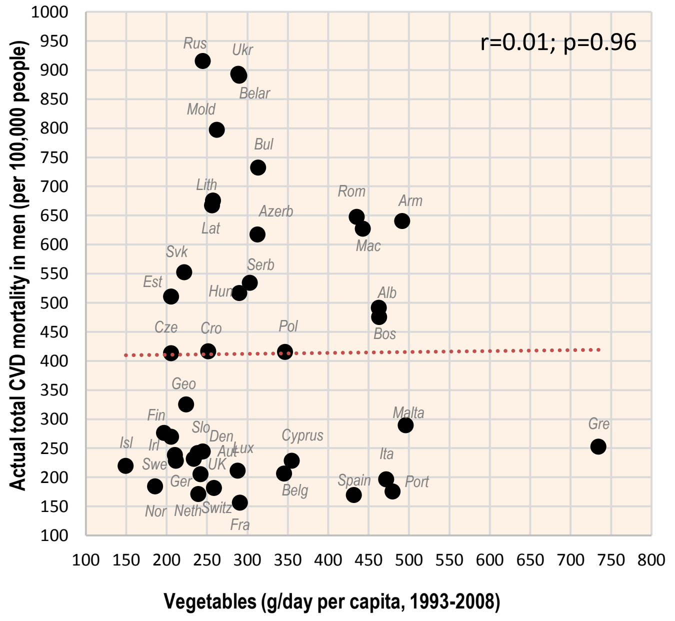

Supplementary Fig. 33. Correlation between the mean consumption of vegetables and the actual total CVD mortality in men (r=0.01; p=0.96).

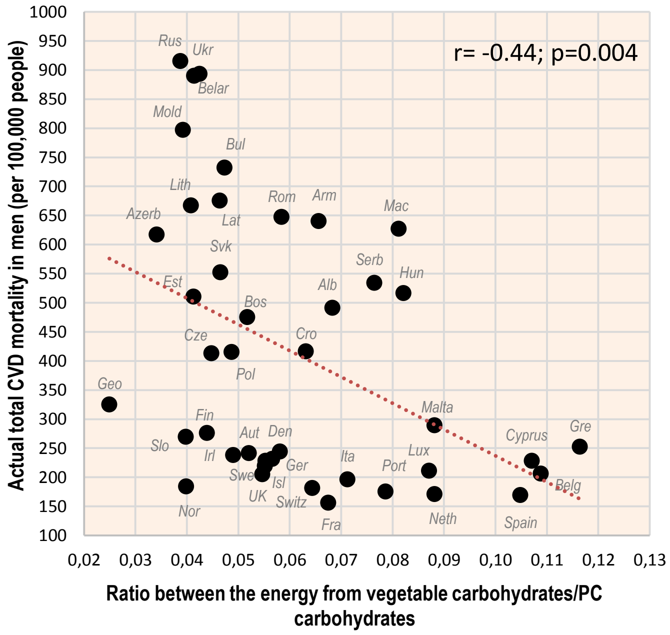

Supplementary Fig. 34. Correlation between the mean vegetable carbohydrates/PC CARB energy ratio and the actual total CVD mortality in men (r= -0.44; p=0.004).

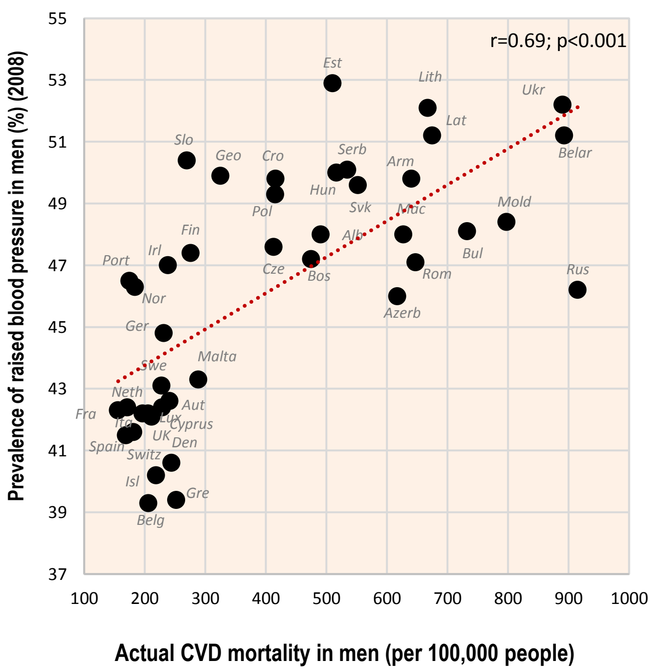

Supplementary Fig. 35. Correlation between the prevalence of raised blood pressure and the actual total CVD mortality in men (r=0.69; p<0.001).

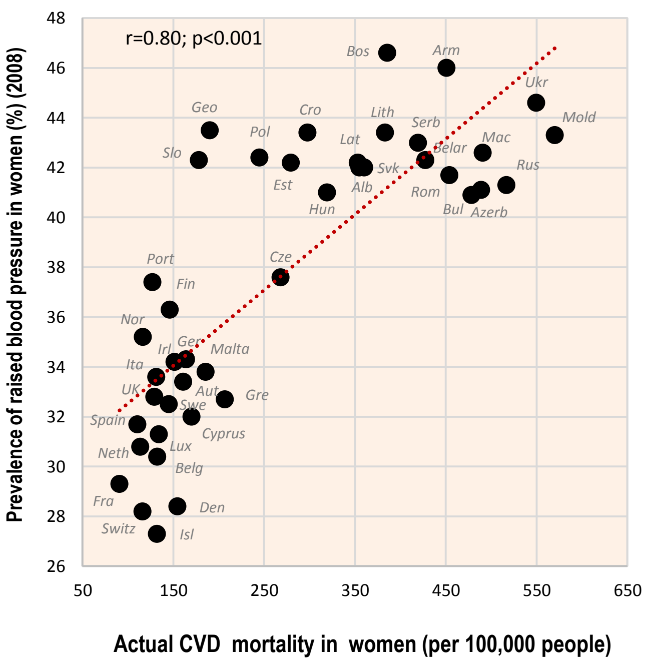

Supplementary Fig. 36. Correlation between the prevalence of raised blood pressure and the actual total CVD mortality in women (r=0.80; p<0.001).

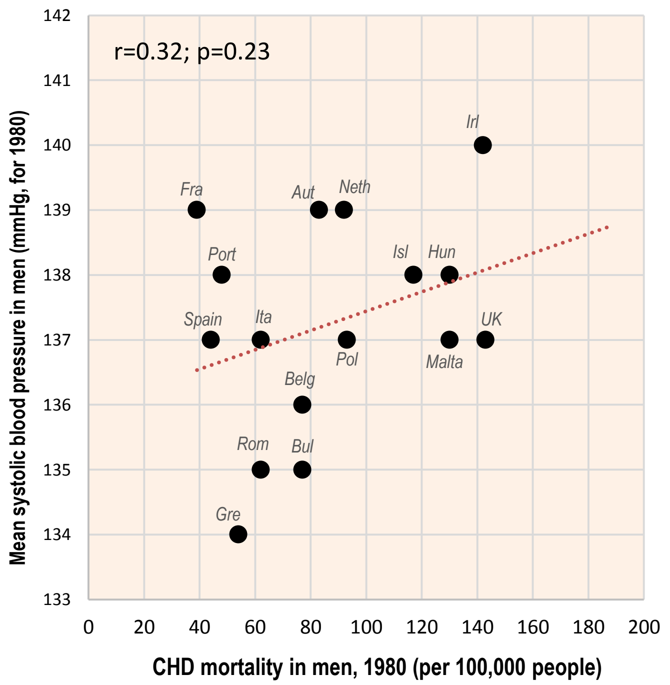

Supplementary Fig. 37. Historical correlation between the mean systolic blood pressure (for 1980) and CHD mortality in men, for 1980 (r=0.32; p=0.23).

Note: This comparison uses only the sample of 16 countries, for which all data in 1980 are available. The correlation in the total sample of 27 available countries is r=0.26 (p=0.18).

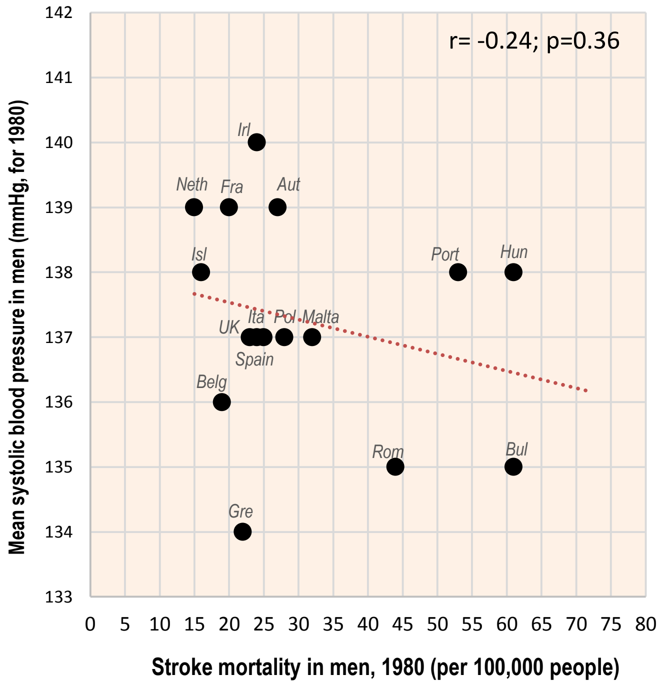

Supplementary Fig. 38. Historical correlation between the mean systolic blood pressure (for 1980) and stroke mortality in men, for 2000 (r= -0.24; p=0.36).

Note: This comparison uses only the sample of 16 countries, for which all data in 1980 are available. The correlation in the total sample of 27 available countries is r= -0.06 (p=0.76).

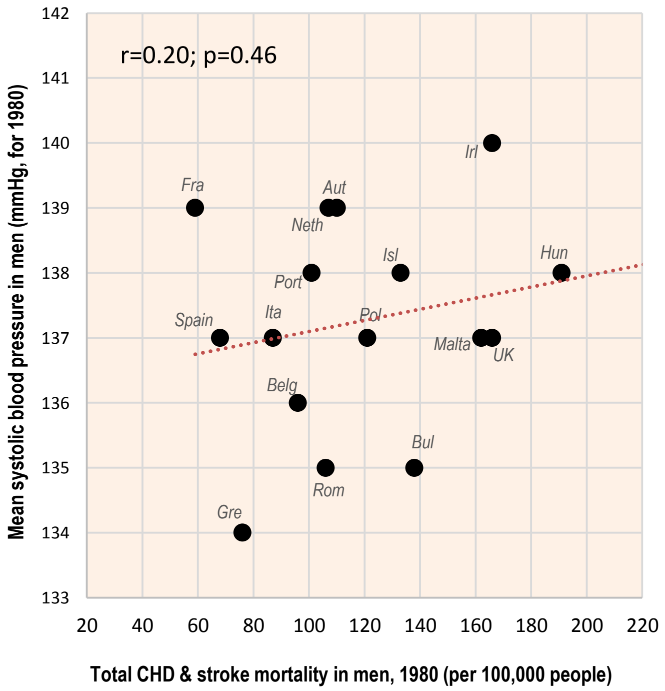

Supplementary Fig. 39. Historical correlation between the mean systolic blood pressure (for 1980) and total CHD & stroke mortality in men, for 1980 (r=0.20; p=0.46).

Note: This comparison uses only the sample of 16 countries, for which all data in 1980 are available. The correlation in the total sample of 27 available countries is r=0.18 (p=0.37).

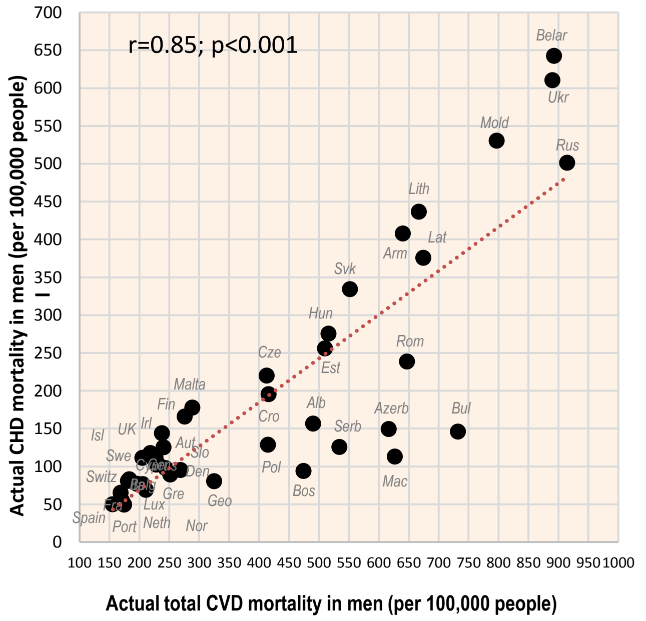

Supplementary Fig. 40. Correlation between the actual CHD mortality and the actual total CVD mortality in men (r=0.85; p<0.001).

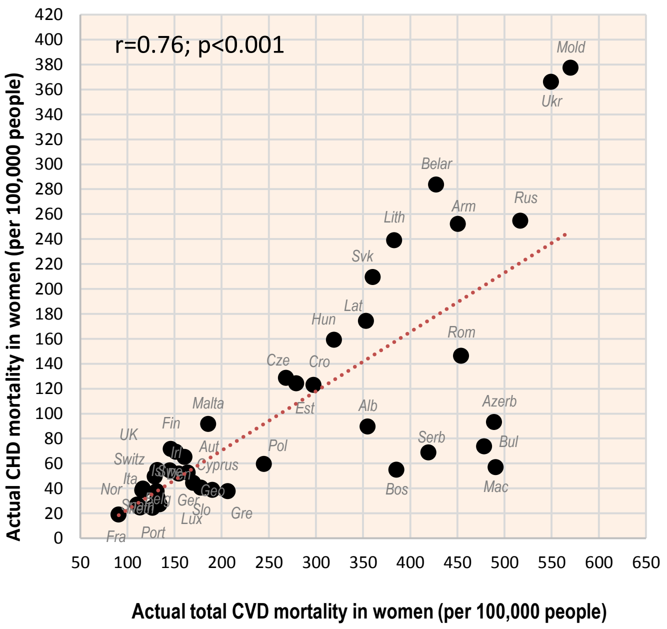

Supplementary Fig. 41. Correlation between the actual CHD mortality and the actual total CVD mortality in women (r=0.76; p<0.001).

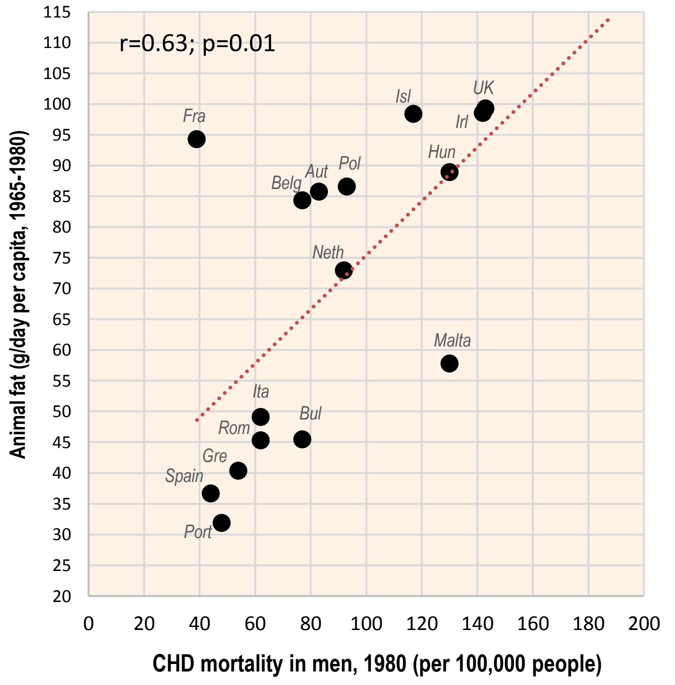

Supplementary Fig. 42. Historical correlation between the mean consumption of animal fat (1965-1980) and CHD mortality in men, for 1980 (r=0.63; p=0.01).

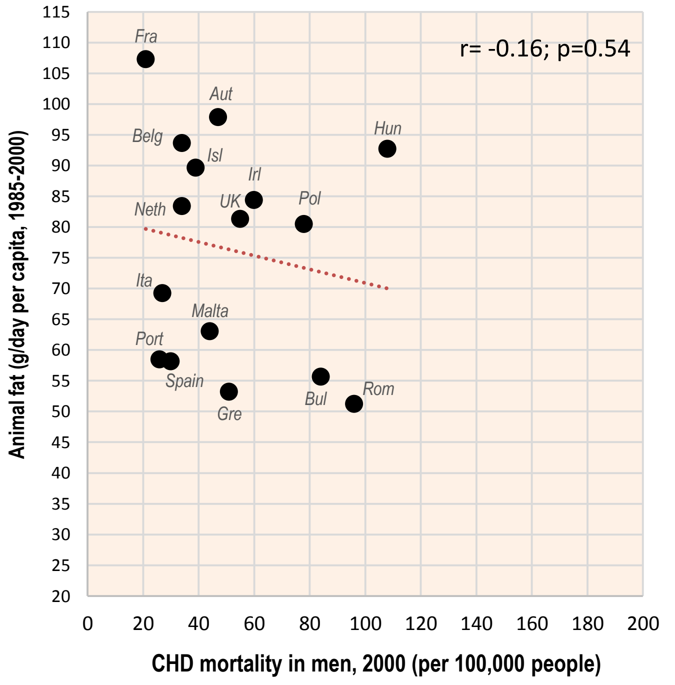

Supplementary Fig. 43. Historical correlation between the mean consumption of animal fat (1985-2000) and CHD mortality in men, for 2000 (r= -0.16; p=0.54).

Note: This comparison uses only the sample of 16 countries, for which all data in 1980 are available.

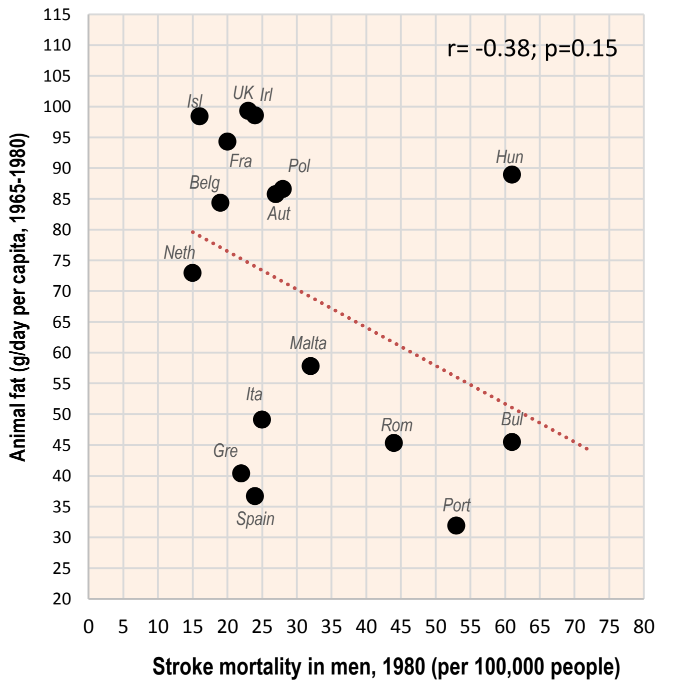

Supplementary Fig. 44. Historical correlation between the mean consumption of animal fat (1965-1980) and stroke mortality in men, for 1980 (r= -0.38; p=0.15).

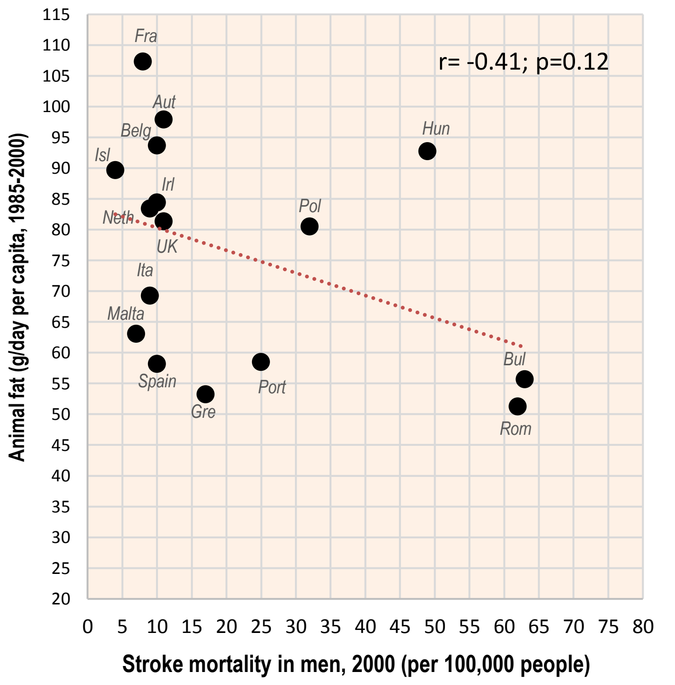

Supplementary Fig. 45. Historical correlation between the mean consumption of animal fat (1985-2000) and stroke mortality in men, for 2000 (r= -0.41; p=0.12).

Note: This comparison uses only the sample of 16 countries, for which all data in 1980 are available.

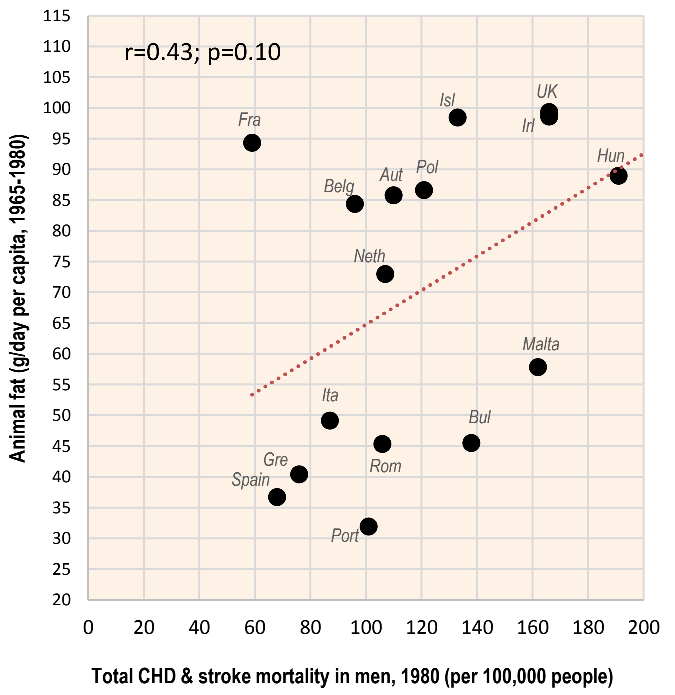

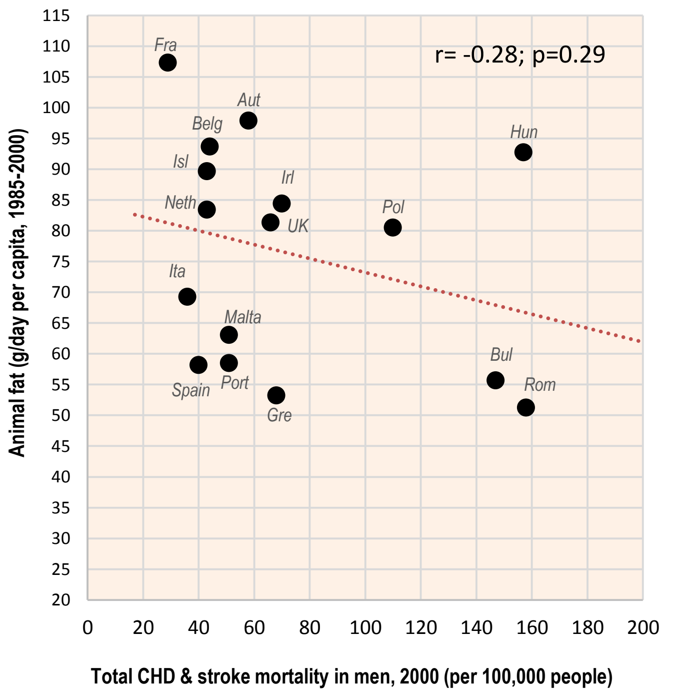

Supplementary Fig. 46. Historical correlation between the mean consumption of animal fat (1965-1980) and total CHD & stroke mortality in men, for 1980 (r=0.43; p=0.10).

Supplementary Fig. 47. Historical correlation between the mean consumption of animal fat (1985-2000) and total CHD & stroke mortality in men, for 2000 (r= -0.28; p=0.29).

Note: This comparison uses only the sample of 16 countries, for which all data in 1980 are available.
